# ﻿The Nereididae (Annelida) – diagnoses, descriptions, and a key to the genera

**DOI:** 10.3897/zookeys.1182.104258

**Published:** 2023-10-11

**Authors:** Robin S. Wilson, Christopher J. Glasby, Torkild Bakken

**Affiliations:** 1 Sciences Department, Museums Victoria Research Institute, Museums Victoria, GPO Box 666 Melbourne, Victoria 3001, Australia Sciences Department, Museums Victoria Research Institute, Museums Victoria Melbourne Australia; 2 The University of Melbourne, Melbourne, Victoria 3010, Australia The University of Melbourne Melbourne Australia; 3 Museum and Art Gallery Northern Territory, PO Box 4646, Darwin NT 0801, Australia Museum and Art Gallery Northern Territory Darwin Australia; 4 Australian Museum Research Institute, Australian Museum, 1 William Street, Sydney, NSW 2010, Australia Australian Museum Research Institute, Australian Museum Sydney Australia; 5 Norwegian University of Science and Technology, NTNU University Museum, NO-7491 Trondheim, Norway Norwegian University of Science and Technology, NTNU University Museum Trondheim Norway

**Keywords:** Computer taxonomy, diagnosis, identification tools, natural language descriptions, polychaete, taxonomic verification, Taxonomic Information System

## Abstract

Nereididae is among the most familiar of marine annelid families, common and well-studied in most marine environments but paradoxically no recent key or identification guide exists to the world’s genera. Here updated generic descriptions, a list of characters, a linear key to genera, and minimal diagnoses that distinguish each genus from all others in the family are provided. This information is generated from a Delta database of 186 morphological characters and a link is provided to downloadable software allowing the same data to be interrogated using the open-source Delta program Intkey – a nonlinear multiple entry point computerised interactive key. For each genus the recent literature is also summarised, comments on taxonomic status provided, and published keys to species cited. Nexus format matrices are provided for all 45 genera and 158 Nereididae species, representing all genera, scored for 146 multistate characters from the same character list to facilitate future phylogenetic studies.

## ﻿Introduction

The Nereididae is probably the best-known of all the families of marine annelids (“polychaetes”) – the family includes many species that are commonly encountered world-wide, intertidally, and also in diverse habitats from ephemeral freshwaters to abyssal depths (Bakken et al. 2022; Rouse et al. 2022). Nereididae are also frequent study subjects in teaching laboratories, and some species are commonly studied as laboratory animals (Fischer et al. 2010) (although now known to comprise complexes of multiple species; see Systematic account of Nereididae genera). Nereididae are almost invariably used as exemplars of marine Annelida in invertebrate zoology textbooks (Marshall and Williams 1972; Ruppert et al. 1994; Rouse and Giribet 2016). Nereididae are significant ecologically especially in intertidal flats (Compton et al. 2013; Choi et al. 2014) where they form significant components to the diet of fish and shorebirds (Iwamatsu et al. 2007; Alves et al. 2013; Duijns et al. 2013) and are aquaculture species of importance for fish bait and for aquaculture species for human consumption (Yoshida 1984; Olive 1994; Lim et al. 2021). A number of nereidid species are accidental introductions, or their introduced status is cryptic (Einfeldt et al. 2014; Villalobos-Guerrero and Carrera-Parra 2015; Tosuji et al. 2019; Kurt et al. 2021).

Taxonomic research on Nereididae is active, with nearly 100 papers published in the past decade, and the family currently comprises 45 genera and 719 species (Read and Fauchald 2023). Prominent among recent studies are investigations using molecular evidence leading to discovery of cryptic species (Glasby et al. 2013; Villalobos-Guerrero and Bakken 2018; Tosuji et al. 2019; Teixeira et al. 2022a, 2022b) and new phylogenetic hypotheses (Tosuji et al. 2018; Alves et al. 2020, 2023; Villalobos-Guerrero et al. 2022b). The past decade has also seen description of 40 new species, revision of eight genera and a review of the diversity, biology, anatomy, and ecology of the family Bakken et al. (2022).

Despite this recent taxonomic progress, identification of nereidid specimens remains a challenge, especially to non-specialists. Genus-level identification is difficult because no key to genera has been published since Fauchald (1977) and our own interactive key, now 20 years out of date (Wilson et al. 2003). Identification difficulties are compounded by the wide recognition that several of the most species-rich genera are assemblages of unrelated species (Bakken and Wilson 2005; Bakken et al. 2022; Rouse et al. 2022). It is clear that a number of genera will be revised when further molecular systematic studies are completed. But it is also clear that achieving sufficient taxon sampling in those molecular studies is a significant challenge and that the composition of many genera and identity of many species is likely to remain doubtful for a significant number of years. Yet the need for identification tools is widespread, not least by those conducting new molecular systematics studies to resolve problematic taxa. For these reasons we provide this review with the following aims:

- to provide updated descriptions of all genera; these correct one error in Bakken et al. (2022) and also include additional characters (see Systematic account of genera);
- to provide a dichotomous key to genera (see Key to genera of Nereididae) and a downloadable interactive identification tool (= Taxonomic Information System) using the Delta Intkey software (Wilson et al. 2023);
- to discriminate all genera based on minimal diagnoses (see Systematic account of genera);
- to provide a character list (see Nereididae characters) and Nexus format data matrices (Wilson et al. 2023) for inclusion in future phylogenetic studies requiring morphological evidence.

## ﻿Methods

We used the Delta (Descriptive Language for Taxonomy) suite of programs to create and manage taxonomic data to support objective and quantitative description and discrimination of Nereididae taxa (Dallwitz 1974, 1980; Dallwitz et al. 1993; Dallwitz and Paine 2015). The original implementation of the Delta software by Australia’s Commonwealth Scientific and Industrial Research Organisation (CSIRO) is still available as Windows only software via Dallwitz (2020). The Delta software was ported to Java by the Atlas of Living Australia as Open-Delta (Atlas of Living Australia 2014) as Windows/Mac OS/Linux software and this is the version we use. A third implementation of Delta, although lacking interactive identification software, is Cavalcanti (2022). The implementation of Delta is approximately identical in Dallwitz (2020) and Atlas of Living Australia (2014) so the guide of Coleman et al. (2010), although based on Windows software, is a very useful introduction for any Delta installation.

Delta comprises several separate applications: the Delta Editor manages taxon by character data and generates outputs via scripts (called Action Sets). For the purposes of this paper, principal outputs are natural language descriptions and diagnoses of taxa (see below and Systematic account of Nereididae genera), linear keys (see below and Key to genera of Nereididae), interactive keys and Nexus files (Wilson et al. 2023) and annotated character lists (see Nereididae characters, below).

For each genus we include two Delta outputs: a description and a diagnosis – terms that have been used loosely in much of the taxonomic literature, where typically “diagnoses” are merely descriptions. Our descriptions are Delta-generated natural language outputs, use all character states known for a genus based on the sources we list as interpreted against our character list. These are concatenations of character states recorded for each taxon.

We concur with Borkent (2021) that diagnoses should be minimal statements that precisely distinguish taxa – typically from other taxa of the same rank – and ours do so. Borkent (2021) did not identify tools for the non-trivial task of generating such diagnoses but the Delta system has that capability: the DiagLevel setting specifies the minimum number of characters for which the diagnostic description should differ from all the other taxa (Dallwitz 1989). The diagnoses provided below include, for all genera, ‘minimal diagnoses’ which as the name implies, are a list of those characters which alone are sufficient to distinguish the given genus from all others. These minimal diagnoses were generated using the Intkey setting DiagLevel=1. For many genera, the setting could be increased to DiagLevel=2, thus generating additional characters which, for those genera, can be used to verify a ‘minimal diagnosis’ that may have been tentatively achieved by the user (perhaps when viewing damaged specimens, or when interpreting some characters was uncertain). These additional diagnostic characters providing an additional secondary level of verification are termed ‘secondary diagnosis’ in the generic accounts below. Our intended use of the diagnosis is to verify identifications by detecting errors that may have been made while using a key (Borkent 2021; also see comment below at the beginning of Key to genera of Nereididae).

The key was generated by the Delta Confor program with the following settings: RBASE = 2.00 ABASE = 1.00 REUSE = 1.01 VARYWT = 0.80; Number of confirmatory characters = 2. Following the recommendation of Dallwitz and Paine (2015) these settings were arrived at by iterative modification: balancing length of key (RBASE), evenness of subdivisions based on abundance indices, (ABASE, not used here), minimising re-use of characters (REUSE) and treatment of variable characters (VARYWT); Dallwitz (1974) provides further details.

Characters and character states are described and illustrated in the following section. The downloadable Intkey interactive key associated with this paper includes a more comprehensive set of character state illustrations. Two Nexus files are provided as Suppl. materials 1, 2 and as part of Wilson et al. (2023). One Nexus file contains all 45 genera, the second contains 158 species representing all nereidid genera (these are the 158 species for which we have the most complete data). Delta truncates character and character state labels in Nexus outputs to 30 characters; these truncated labels were replaced with full names from original Delta text files using shell scripts provided by Buz Wilson (pers. comm. 2 September 2023) and subsequent manual editing. Both Nexus files use the same 146 unordered multistate characters; both exclude meristic characters since their coding for phylogenetic analyses requires additional data and assumptions (e.g., Lawing et al. 2008) and was beyond the scope of this project. All these information sources are generated from the same Delta database that was used for the diagnoses and descriptions.

Interactive keys are implemented in the Delta suite by the Intkey application (Dallwitz et al. 1995; Coleman et al. 2010) which requires binary files generated from the Delta Editor (Penev et al. 2009). Our Intkey files for Nereididae are available as a separate download (Wilson et al. 2023) and require prior installation of the (recommended) Open-Delta software (Atlas of Living Australia 2014) or the original Delta programs (Dallwitz 2020). This paper serves as an alternative for those unable to install the Delta software.

## ﻿Results and discussion

### ﻿Annotated characters of Nereididae

We identified 186 morphological characters to characterise nereidid taxa. Characters are given as they are described in the Delta Editor with annotations and illustrations as required, elaborating features in more detail. We have illustrated characters that we consider the most useful for identification using specimens lodged with the Museum & Art Gallery of the Northern Territory (NTM) and Museum Victoria (NMV), or derived from the literature as credited in the figure captions. Some characters are included even though their potential to inform higher level relationships are not yet tested, for example: palpophore surface (character 4), and prostomium longitudinal groove (character 9). We have also included some characters useful for distinguishing species (principally counts of papillae and paragnaths on the eversible pharynx). Meristic characters, in particular paragnath counts, are thus far rarely used to characterise nereidid genera – typically such characters require data from large numbers of specimens if they are to be the basis of robust taxonomic conclusions (Wilson 1993; Wilson and Glasby 1993).

This character list is also the basis of the Nexus format files provided by Wilson et al. (2023). That Nexus file excludes meristic characters for the reasons set out above, thus the 186 characters listed below are reduced to 146 multistate characters in Nexus outputs. Nexus file labels of character descriptions in some are abbreviated for convenience in phylogenetic software; all Nexus character labels are provided below in square brackets.

#### ﻿Prostomium, pharynx, and ventrum – characters 1–20 (Fig. 1A–P)

1. Antennae [NEXUS: antennae]

1. present.
2. absent.

All Nereididae have a pair of antennae excepting *Unanereis* Day, 1962 in which a single antenna is present as illustrated in Day (1962: fig. 3a) (*Unanereismacgregori*) and Ben Amor (1980: fig. A) (*Unanereiszghali*). Bakken et al. (2022) discussed the possibility that presence of a single antenna is a developmental anomaly seen occasionally in Nereididae specimens, in which case both species of *Unanereis* may be referable to *Ceratonereis* or *Solomononereis*.

2. Palps [NEXUS: palp orientation]

1. anteriorly directed.
2. ventrally directed.

In most nereidids the palps are anteriorly directed and both palpophore and palpostyles are typically easily seen in dorsal view e.g., Villalobos-Guerrero et al. (2022b: fig. 5b, c) for *Nereisagulhana*. However, in some taxa, e.g., *Micronereis* and some *Kainonereis* and *Platynereis* species, the palps are distinctly ventrally directed and not fully visible in dorsal view. Examples of ventrally directed palps are Paxton (1983: fig. 15) for *Micronereisbansei*, Conde-Vela et al. (2018: fig. 3b, c) for *Kainonereisalata* and Read (2007: fig. 6a, b) for *Platynereisaustralis*. Caution is required utilising this character alone for identification since, at least in *Kainonereis* and *Platynereis*, this condition seems to be expressed in epitokes and not always confirmed for atokes. Furthermore, it is plausible that palps which may have been ventrally directed in live epitokes may be distorted into an anteriorly directed palps orientation as an artefact caused by preservation with pharynx extended (Read 2007: figs 2a, 3a); Read (2007) did not make use of this character to separate New Zealand species of *Platynereis*.

**Figure 1.**
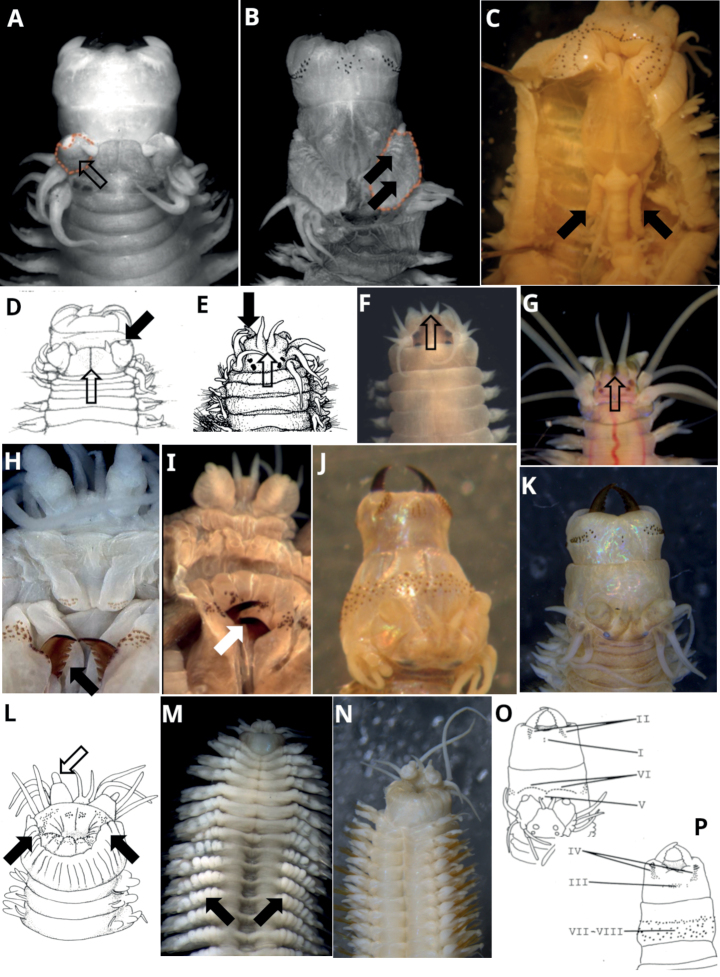
Prostomium, pharynx **A** barrel-shaped palps (orange dashed outline) and palpophore surface with a single transverse groove (open arrow) *Namalycastisabiuma* LNG M16Q3 **B** subconical palps (orange dashed outline) and palpophore surface with several oblique grooves (filled arrows) *Neanthesglandicincta* MD165 anterior **C** caecal glands (filled arrows) in ventral dissection of *Perinereisvallata*NMV F108784 **D** spherical palpostyles (filled arrow) and prostomium with longitudinal groove (open arrow) *Namalycastisabiuma***E** acutely conical palpostyles (filled arrow) and indented prostomium (open arrow) *Gymnonereisminyami***F** prostomium anterior margin entire *Nereis* sp. **G** prostomium anterior margin indented (open arrow) *Ceratonereis* sp. **H** dentate jaws (filled arrow) *Platynereisbicanaliculata*NTM W17252 **I** smooth/crenulate jaws (white arrow) *Leonnatescrinitus*NTM W3330 **J** everted pharynx a truncate cone with greatest width at margin of tentacular belt (= “frustrum-shaped”) *Alitta* sp. NMV F94547 **K** everted pharynx cylindrical *Simplisetiaaequisetis*NMV F94248 **L** ventral peristomial flap (filled arrows) and palpostyles subconical to oval-shaped (open arrow) *Cheilonereisperistomialis***M** ventrum of anterior chaetigers with rows of tubercles extending to the base of each neuropodium (filled arrows) *Australonereisehlersi***N** ventrum of anterior chaetigers smooth *Neanthes* sp. NMV F182608 **O** dorsal view pharynx with numbering following Kinberg *Perinereisvallata***P** ventral view pharynx with numbering following Kinberg *Perinereisvallata*. Sources: **A, B, F, H, I, M** C. Glasby photographs **C, J, K, N** R. Wilson photographs **D** modified after C. Glasby (1999)**E** modified after Hutchings and Reid (1990)**G** Leon Altoff photograph **L, O, P** R. Wilson drawings. Not to scale; body widths of these example specimens are in the range 2–5 mm wide excluding parapodia.

3. Palpophore [NEXUS: palpophore form]

1. barrel-shaped, approximately equal width from base to palpostyle (not overly large compared with palpostyle; Fig. 1A).
2. massive subconical, flattened palpostyle (minute by comparison; Fig. 1B).

Although palpophore shape has been considered in recent revisionary works at the generic and species group levels, there has been no convincing arguments identifying particular shapes. At least four characteristic shapes have been documented: oval (*Perinereis* species complex) sub-ovoid (*Composetia*, *Leonnates*), obtusely conical (*Leonnates*, *Parasetia*, *Potamonereis*), and subconical (*Parasetia*, *Neanthes*) (Villalobos-Guerrero 2019; Villalobos-Guerrero et al. 2021, 2022a, b). However, some genera were found to be polymorphic for the states recognised and the states themselves can be difficult to distinguish, especially if specimens are preserved in a distorted condition. Other authors have distinguished palpophores on the basis of size: e.g., the palpophores of *Potamonereis* have been referred to as massive (as in *Potamonereiskumensenis* (Sato, 2020)). The present 2-state system is an attempt to describe more effectively the variation in the family. Thus, barrel-shaped palpophores (most genera) have an approximately equal width from base to palpostyle. They are not overly large compared to the size of the palpostyle, and in some cases may be of similar length and usually they have a transverse groove (Fig. 1A). Variability in length of barrel-shaped palpophores may be an indication that this character needs to be further divided into additional states. Subconical, dorso-ventrally flattened palpophores (*Alitta*, *Hediste*, *Leonnates*, *Neanthesmicromma*, *Neanthesglandicincta*; Fig. 1B) tend to be massive compared to the size of the palpostyle, have a maximum width at mid to mid-end of palp, and usually have longitudinal striae. *Neanthes* is polymorphic for this character and *Dendronereis* is uncertain.

4. Palpophore surface [NEXUS: palpophore surface]

1. without grooves or striae (palps short, compact; Fig. 1D).
2. with a single transverse groove (palpophores well developed (Fig. 1A).
3. with several oblique grooves or striae (palpophores well developed; Fig. 1B).

The presence of a transverse groove (Fig. 1A, open arrow) or multiple striae (Fig. 1B, filled arrows) on the palpophore, as noted by Villalobos-Guerrero and Idris (2021) is present in many nereid genera with biarticulated palps. The depth of the groove is variable depending on how extended the palps were on fixation. Sometimes many finer transverse grooves (striae) are visible although they may be faint if the palps are extended. In general, we observed that barrel-shaped forms have a single transverse groove which is perpendicular to the long axis of the palpophore, and that subconical, flattened forms possess multiple striae which are at an oblique angle.

5. Palpostyles [NEXUS: palpostyles]

1. present.
2. absent (palps undivided, minute).

A biarticulate palp with a distinct distal palpostyle is present in all Nereididae except members of the genus *Micronereis* which have an undivided roughly spherical palp that is also ventrally located on the anterior prostomium; see Paxton (1983: fig. 15; *Micronereisbansei*).

6. Palpostyles for m [NEXUS: palpostyles form]

1. spherical.
2. subconical.
3. acutely conical.

Palpostyles (present in all genera except *Micronereis*) are recognised as having three shapes: spherical, subconical (in most nereidids; could also be referred to as oval-shaped), and acutely conical. The palpostyles are spherical in members of the Namanereidinae, as illustrated by Glasby (1999: fig. 10a; *Namalycastisabiuma*). Some members of the genera *Ceratocephale* and *Gymnonereis* have palpostyles that are acutely conical e.g., Hutchings and Reid (1990: fig. 6a; *Ceratocephaleaureola*), Hutchings and Reid (1990: fig. 9a; *Gymnonereisminyami*), Wilson and Glasby (1993: fig. 8a; *Perinereiscaeruleis*). This character is also useful for species separation across several unrelated genera of Nereididae.

7. Eyes [NEXUS: eyes]

1. present (Fig. 1E).
2. absent (Fig. 1D).

Two pairs of eyes are present in most Nereididae but they are absent in a number of species found at bathyal and abyssal depths and subterranean and cave-dwelling Namanereidinae. Examples of the former are illustrated by Hartman and Fauchald (1971: pl. 4 fig. a; *Ceratocephaleabyssorum*, as *Pisionuraabyssorum*) and Hartmann-Schröder (1975: fig. 22; *Neanthesbioculata*).

8. Prostomium anterior margin [NEXUS: prostomium anterior margin]

1. entire (Fig. 1F).
2. indented (Fig. 1E, G, open arrows).

The prostomium in Nereididae is usually entire on the anterior margin e.g., in *Composetiamarmorata* (Glasby 2015: fig. 1G, H) but in 13 genera there is a conspicuous indentation between the antennae, as illustrated by Hutchings and Reid (1990: fig. 10a; *Gymnonereisyurieli*), Glasby (1999: fig. 12a; *Namalycastisborealis*) and Glasby (2015: fig. 1A–E; *Ceratonereis* spp.).

9. Prostomium longitudinal groove [NEXUS: prostomium longitudinal groove]

1. present (Fig. 1D open arrow).
2. absent (Fig. 1E, F).

10. Tentacular belt length [NEXUS: tentacular belt length]

1. equal to or less than length of chaetiger 1.
2. greater than length of chaetiger 1.

Terminology for characters 10 and 11 follows Villalobos-Guerrero et al. (2022a) who as part of a revision of *Composetia* showed that the body part referred to widely in the literature as “tentacular segment”, “achaetigerous segment” and “apodous segment” comprises two segments plus the peristomium. While some genera are polymorphic for this character, others consistently have one or the other state. A short tentacular belt (state 1) distinguishes *Namalycastis* and *Namanereis* from most other nereidids.

11. Tentacular belt [NEXUS: tentacular belt divided]

1. fused, separate segments not recognisable.
2. represented by two distinct segments each carrying a pair of tentacular cirri.

12. Tentacular cirri comprising [NEXUS: tentacular cirri number]

1. four pairs.
2. three pairs.

13. Tentacular cirrophores [NEXUS: tentacular cirrophores]

1. present.

2. absent (cirri undivided).

14. Tentacular cirri extending to chaetiger (number)

Small variations in the length of these cirri on the tentacular belt is often not a useful statistic, but may assist in recognising taxa which have very long tentacular cirri (e.g., *Ceratonereis* spp. and *Platynereis* spp.). Generally the posterodorsal pair is the longest.

15. Ventral peristomial flap [NEXUS: ventral peristomial flap]

1. present (Fig. 1L, filled arrows).
2. absent.

16. Ventrum of anterior chaetigers [NEXUS: ventrum anterior chaetigers]

1. smooth (Fig. 1N).
2. with rows of tubercles extending to the base of each neuropodium (Fig. 1M).

17. Oesophageal caeca [NEXUS: oesophageal caeca]

1. present (Fig. 1C).
2. absent.

Oesophageal caeca (equivalent terms are caecal glands, oesophageal pouches) are a pair of organs that are prominent and easily visible if present, but only by dissection. The oesophageal caeca are located immediately posterior to the muscular pharynx. at the start of the oesophagus. Their utility in taxonomy was first suggested by Savigny (1822) but Khlebovich (2001) was the first to use these structures in the taxonomy of Nereididae.

18. Jaws [NEXUS: jaw dentition]

1. with smooth or slightly crenulate cutting edge (Fig. 1I).
2. with dentate cutting edge (Fig. 1H).

Variation in jaw morphology is as yet not well understood. Some taxa have jaws with smooth or faintly crenulate cutting edge, while others have distinctly or indistinctly toothed jaws. In some taxa the jaws are robust and dark (e.g., *Neanthes* spp., *Perinereis* spp.), while others have finer, translucent jaws (e.g., *Ceratocephale* spp., some *Simplisetia* spp.). However, many taxa are intermediate between these conditions, and abrasion may falsely result in the appearance of “smooth” jaws. Differences in the chemical composition of nereidid jaws may offer the best opportunity to distinguish taxa, but these studies have yet to be undertaken systematically.

19. Everted pharynx shape [NEXUS: everted pharynx form]

1. cylindrical (Fig. 1K).
2. a truncate cone, tapering, greatest width at margin of tentacular segment (Fig. 1J).

This character was introduced by Villalobos-Guerrero et al. (2021) using the term “frustrum-shaped” however we prefer simpler language (and frustrum can also refer to a truncate pyramid).

20. Maxillary ring of pharynx [NEXUS: maxillary ring of pharynx]

1. divided into discrete Areas (Fig. 1O, P).
2. undivided.

#### ﻿Pharyngeal papillae and paragnaths – characters 21–82 (Fig. 2A–G)

21. Maxillary ring of pharynx with papillae [NEXUS: maxillary ring papillae]

1. present.
2. absent.

22. Maxillary ring of pharynx with papillae [NEXUS: maxillary papillae arrangement]

1. solitary.
2. in tufts.

23. Maxillary ring of pharynx with papillae number

A count of the total number of papillae on the maxillary ring helps to discriminate taxa and is practical even when the arrangement in discrete Areas is unclear, as is often the case in Gymnonereidinae.

24. Undivided maxillary ring – total number of paragnaths present

*Micronereis* is the only nereidid genus with paragnaths present on an undivided maxillary ring. In this genus the pharynx is not fully eversible and specimens are small – the number of paragnaths present can only be recorded as a single number, if it can be determined at all.

**Figure 2.**
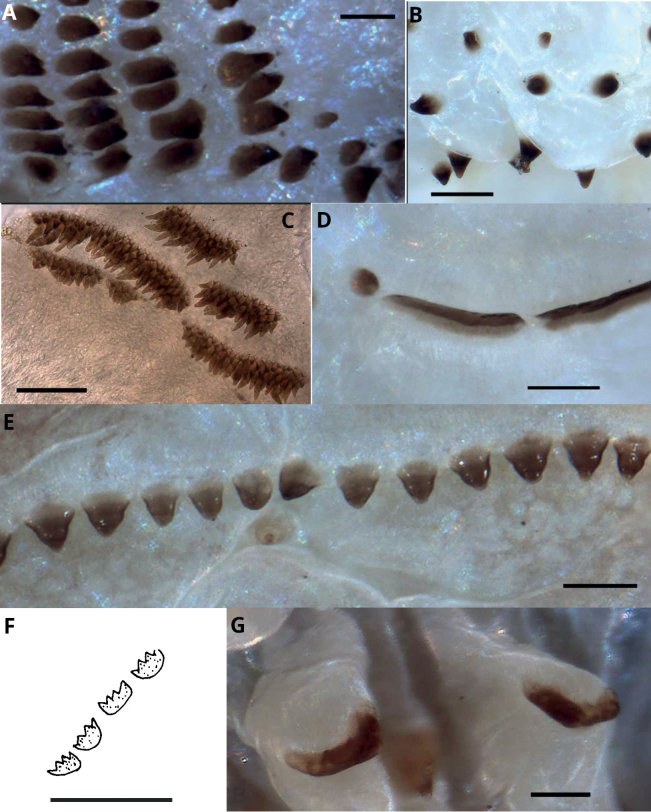
Paragnaths **A** P-bar paragnaths *Pseudonereisanomala***B** conical paragnaths *Pseudonereistrimaculata***C** rod-like paragnaths *Platynereispolyscalma***D** smooth bar paragnaths *Perinereisvancaurica***E** pyramidal paragnaths *Perinereisakuna***F** crown paragnaths *Micronereispiccola***G** shield-shaped paragnaths *Pseudonereistrimaculata*. Sources: **A–E, G** emended from Bakken et al. (2009: figs 2–5) **F** emended from Paxton (1983: fig. 4). Scale bars: 0.1 mm (**A–E, G**); 200 μm (**F**).

25. Maxillary ring paragnaths [NEXUS: maxillary ring paragnaths]

1. present.
2. absent.

26. Maxillary ring of pharynx with P-bar paragnaths (Fig. 2A) [NEXUS: maxillary ring Pbars]

1. present, usually in regular comb-like rows.
2. absent.

27. Area I conical paragnaths (Fig. 2B) [NEXUS: Area I conical paragnaths]

1. present.
2. absent.

28. Area I conical paragnaths: number

29. Area II conical paragnaths [NEXUS: Area II conical paragnaths]

1. present.
2. absent.

30. Area II conical paragnaths: number

31. Area II rod-like paragnaths (Fig. 2C) [NEXUS: Area II rodlike paragnaths]

1. present.
2. absent.

32. Area II rod-like paragnaths: number

33. Area III conical paragnaths [NEXUS: Area III conical paragnaths]

1. present.
2. absent.

34. Area III conical paragnaths: number

35. Area III conical paragnaths: isolated lateral groups [NEXUS: Area III lateral groups]

1. present.
2. absent.

In many taxa Area III paragnaths include a few paragnaths positioned as distinct groups on each side of the main group.

36. Area III rod-like paragnaths [NEXUS: Area III rodlike paragnaths]

1. present.
2. absent.

37. Area III rod-like paragnaths: number

38. Area IV paragnaths [NEXUS: Area IV paragnaths]

1. present.
2. absent.

39. Area IV conical paragnaths [NEXUS: Area IV conical paragnaths]

1. present.
2. absent.

40. Area IV conical paragnaths: number

41. Area IV smooth bar-like paragnaths (Fig. 2D) [NEXUS: Area IV smooth bars]

1. present.
2. absent.

These are the smooth bar paragnaths of Bakken et al. (2009) and are not formed by fusion of separate conical paragnaths but are apparently present throughout development in the form of smooth bars in all those taxa in which they occur. When they occur on Area IV, cones are also usually present (sometimes lacking, see Tosuji et al. 2019), as illustrated in Hutchings et al. (1991: fig. 3B; *Perinereisamblyodonta*) and Villalobos-Guerrero et al. (2022b: fig. 4B; *Neanthescapensis*).

Thus, bar-like paragnaths are distinct from “melted paragnaths” described and illustrated by Bakken et al. (2009: fig. 2c) which are formed by partial fusion of distinct paragnaths and occur most often in epitokes and are not considered to have taxonomic value. Glasby et al. (2011) clarified that the term “melted” should apply only to conical paragnaths mounted on a plate-like basement while Conde-Vela and Salazar-Vallejo (2015) introduced the term “merged paragnaths” for forms where a basement is not present. We have not used merged or melted paragnaths here since their intra-specific variability is incompletely understood.

42. Area IV smooth bar-like paragnaths: number

Paragnaths on Area IV are typically roughly conical in shape, though variations range from flattened domes of irregular shape to tooth-like paragnaths. In some taxa, particularly species of *Neanthes* and *Perinereis*, in addition to cones a separate patch of bar-shaped paragnaths occurs at the maxillary end of Area IV; these bars are counted separately.

43. Area IV rod-like paragnaths [NEXUS: Area IV rodlike paragnaths]

1. present.
2. absent.

44. Area IV rod-like paragnaths: number

45. Oral ring papillae [NEXUS: oral ring papillae]

1. present.
2. absent.

46. Oral ring papillae: number

47. Oral ring papillae arrangement [NEXUS: oral ring papillae arrangement]

1. solitary.
2. arranged in tufts.

48. Area V papillae [NEXUS: Area V papillae]

1. present.
2. absent.

49. Area V papillae: number

In *Ceratocephale* spp., Area V and VI contain up to three papillae in total; these are here interpreted as all occurring in Area V, with VI = 0,0.

50. Area VI papillae [NEXUS: Area VI papillae]

1. present.
2. absent.

51. Area VI papillae: number

52. Areas VII-VIII papillae [NEXUS: Areas VIIVIII papillae]

1. present.
2. absent.

53. Areas VII-VIII papillae: number

54. Areas VII-VIII papillae arranged [NEXUS: Areas VIIVIII papillae rows]

1. in a single row.
2. in a double row.

Where a double row of papillae is present, the 2^nd^ (posterior) row may be hard to see unless the pharynx is completely everted.

55. Oral ring paragnaths [NEXUS: oral ring paragnaths]

1. present.
2. absent.

56. Oral ring paragnaths (discrete or continuous) [NEXUS: oral ring paragnaths arranged]

1. with Areas V, VI and VII-VIII discrete.
2. comprising a continuous ring dorsally and ventrally, discrete groups not recognisable.

57. Oral ring paragnaths on Areas V and VI (discrete or continuous) [NEXUS: Areas VVI paragnaths]

1. form discrete groups.
2. continuous, not recognisably distinct.

58. Oral ring pyramidal paragnaths (Fig. 2E) [NEXUS: oral ring pyramidal paragnaths]

1. present.
2. absent.

Pyramidal paragnaths have a quadrangular base and taper to a pointed apex (Bakken et al. 2009: 309).

59. Crown-shaped oral ring paragnaths (Fig. 2F) [NEXUS: oral ring crown paragnaths]

1. present.
2. absent.

60. Crown-shaped oral ring paragnaths: number

61. Area V conical paragnaths [NEXUS: Area V conical paragnaths]

1. present.
2. absent.

62. Area V conical paragnaths: number

63. Area V conical paragnaths arranged [NEXUS: Area V cones arranged]

1. in a triangle.
2. in a longitudinal line.
3. irregularly.

64. Area VI paragnaths [NEXUS: Area VI paragnaths]

1. present.
2. absent.

65. Area VI paragnaths arranged [NEXUS: Area VI paragnaths arranged]

1. in a roughly circular group.
2. in lines or arcs.

Area VI paragnaths are usually arranged in a circular or irregular compact group (sometimes of only one or two paragnaths). In some species of *Neanthes*, an alternative arrangement of cones occurs: a distinct line or arc.

66. Area VI conical paragnaths [NEXUS: Area VI conical paragnaths]

1. present.
2. absent.

67. Area VI conical paragnaths: number

68. Area VI smooth bars (Fig. 2D) [NEXUS: Area VI smooth bars]

1. present.
2. absent.

Tosuji et al. (2019) demonstrated that in some species of *Perinereis*, long smooth bars in Area VI may shorten in length with growth of the worm to the extent that Area VI paragnaths in mature forms show a mixture of short bars and cones, so care must be exercised in using this character.

69. Area VI smooth bars: number

70. Area VI shield-shaped bars (Fig. 2G) [NEXUS: Area VI shield-shaped bars]

1. present.
2. absent.

Shield-shaped bars are laterally compressed and have a pointed or rounded apex (Bakken et al. 2009: 311).

71. Area VI shield-shaped bars: number

72. Area VI rod-shaped paragnaths [NEXUS: Area VI rod paragnaths]

1. present.

2. absent.

73. Area VI rod-shaped paragnaths: number of rows

74. Areas VII-VIII paragnaths [NEXUS: Areas VIIVIII paragnaths]

1. present.
2. absent.

75. Areas VII-VIII conical paragnaths [NEXUS: Areas VIIVIII cone paragnaths]

1. present.
2. absent.

76. Areas VII-VIII conical paragnaths: number

Areas VII-VIII typically forms a continuous ventro-lateral band of paragnaths and is recorded as such. In a few taxa the Areas VII-VIII band of paragnaths is extended through the dorsal region and encircles the oral ring of the pharynx; in this case even though the band nominally extends through the dorsal Areas V and VI, they are indistinguishable and the count is recorded for Areas VII-VIII.

77. Areas VII-VIII conical paragnaths arranged [NEXUS: Areas VIIVIII cones arranged]

1. in isolated patches.
2. in one or more irregular lines forming a continuous band.

In a few nereidid species, e.g., *Cheilonereisperistomialis* Benham, 1916, the paragnaths on Areas VII-VIII are arranged in distinct isolated patches. In other nereidids the arrangement is an irregular but continuous band made up one or more rows deep.

78. Areas VII-VIII conical paragnaths (size distribution) [NEXUS: Areas VIIVIII cones sizes]

1. similar in size, or irregular mix of large and small paragnaths in a single band.
2. differentiated, with a separate band of minute paragnaths also present.

Typically the paragnaths on Areas VII-VIII comprise a variety of sizes irregularly arranged. However, in some taxa there is differentiation into an anterior band of paragnaths similar in size to elsewhere on the proboscis, and a separate band of minute paragnaths.

79. Areas VII-VIII P-bar paragnaths (Fig. 2A) [NEXUS: Areas VIIVIII Pbars]

1. present.
2. absent.

80. Areas VII-VIII P-bar paragnaths (interspersed/discrete) [NEXUS: Areas VIIVIII Pbar arrangement]

1. interspersed with conical paragnaths.
2. forming a separate band.

81. Areas VII-VIII rod-shaped paragnaths (Fig. 2C) [NEXUS: Areas VIIVIII rod paragnaths]

1. present.
2. absent.

82. Areas VII-VIII rod-shaped paragnaths: number of rows

#### ﻿Dorsal lamellae and parapodia – characters 83–128 (Fig. 3A–G)

**Figure 3.**
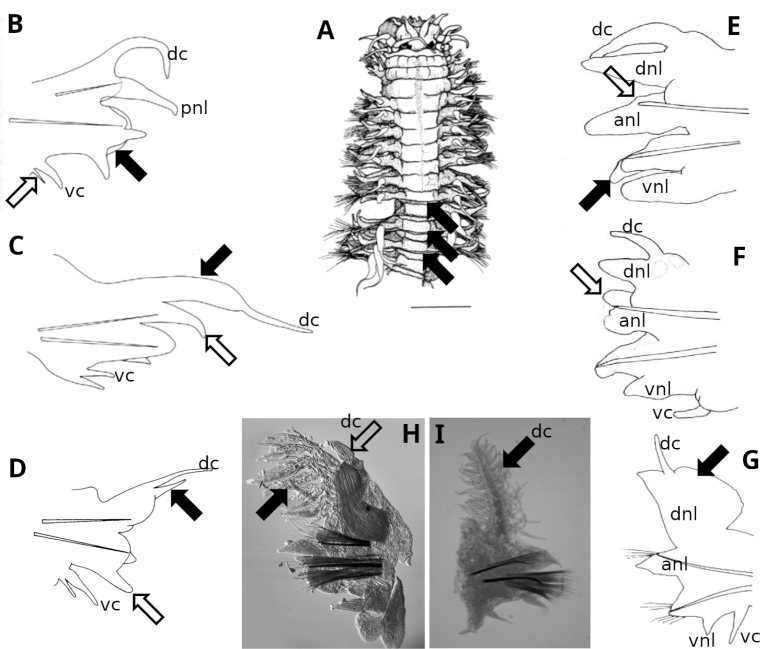
Parapodia **A** transverse dorsal lamellae (filled arrows) *Ceratocephalesetosa***B** flattened post-chaetal neuropodial lobe (filled arrow), accessory ventral cirrus (open arrow) anterior parapodium anterior view *Ceratocephalesetosa***C** cirrophore of dorsal cirrus enlarged and vascularised (filled arrow), acicular notopodial ligule present (open arrow) posterior parapodium anterior view *Ceratocephalesetosa***D** dorsal notopodial ligule (= accessory dorsal cirrus of some authors) (filled arrow) ventral neuropodial ligule (open arrow) *Gymnonereisminyami* chaetiger 34 anterior view **E** notopodial acicular process (open arrow) digitiform neuropodial postchaetal lobe (filled arrow) *Neanthestasmani* chaetiger 30 anterior view (ventral cirrus missing) **F** notopodial prechaetal lobe (open arrow) digitiform neuropodial postchaetal lobe (filled arrow) *Neanthestasmani* chaetiger 30 anterior view **G** dorsal notopodial ligule markedly broader on posterior chaetigers (filled arrow) chaetiger 78 *Alittasuccinea* USNM 27799 **H** dorsal notopodial ligule divided into branchiae (filled arrow) dorsal cirrus (open arrow) *Dendronereidesheteropoda* chaetiger 19 anterior view **I** dorsal cirrus divided into branchial filaments (filled arrow) *Dendronereis* sp chaetiger 14 anterior view. Sources: **A–D**Hutchings and Reid (1990)**E, F** modified after Bakken (2002)**G** R. Wilson drawing **H, I** C. Glasby photographs. Abbreviations: anl, acicular notopodial ligule; dc, dorsal cirrus; dnl, dorsal notopodial ligule; pnl, prechaetal notopodial ligule; vnl, ventral neuropodial ligule; vc, ventral cirrus. Not to scale; maximum body width excluding parapodia of 3A specimen ~ 1.2 mm; for remaining figures acicula lengths in the range 0.1–0.4 mm.

83. Transverse dorsal lamellae (Fig. 3A) [NEXUS: transverse dorsal lamellae]

1. present.
2. absent.

84. Transverse dorsal lamellae, commencing chaetiger

85. Transverse dorsal lamellae, last present chaetiger

86. Transverse dorsal lamellae, mid-dorsal papilla [NEXUS: middorsal papilla]

1. present.
2. absent.

It has been shown by Blake (1985) and Hilbig (1997) (for *Ceratocephaleloveni*) and by Hylleberg and Nateewathana (1988) (for *Ceratocephaleandaman*) that presence/absence of mid-dorsal papilla is variable and likely to be related to size or sexual maturity. However, the description of *Ceratocephalepapillata* de León-González & Góngora-Garza, 1992 is based on 155 specimens, all of which have mid-dorsal papilla. In other species of *Ceratocephale*, observations on mid-dorsal papillae should be interpreted with caution.

87. Transverse dorsal lamellae mid-dorsal papilla commencing chaetiger

88. Notopodium [NEXUS: notopodium development]

1. with at least one distinct ligule or lobe.
2. strongly reduced, without distinct lobes or ligules.

According to nautical wisdom, boats are defined as vessels able to be carried on ships. Parapodial lobes and ligules are distinguished according to a similar logic: lobes can be carried on ligules, but not vice versa. In general, ligules are larger and flatter than the smaller, conical lobes.

89. Dorsal notopodial ligule (Fig. 3E) [NEXUS: dorsal notopodial ligule]

1. present.
2. absent.

90. Dorsal notopodial ligule, first present [NEXUS: dorsal notopodial ligule first]

1. chaetiger 1.
2. chaetiger 3.
3. chaetiger 4.
4. chaetiger 5.

91. Dorsal notopodial ligule, length on anterior chaetigers [NEXUS: dorsal notopodial ligule anterior]

1. markedly elongate.
2. not markedly elongate.
3. markedly reduced.

92. Dorsal notopodial ligule, length on posterior chaetigers [NEXUS: dorsal notopodial ligule posterior]

1. markedly elongate (Fig. 3D).
2. not markedly elongate.

93. Dorsal notopodial ligule, breadth on posterior chaetigers [NEXUS: dorsal notopodial ligule posterior width]

1. markedly broader (Fig. 3G).
2. not markedly broader.

94. Dorsal notopodial ligule, reduction on posterior chaetigers [NEXUS: dorsal notopodial ligule posterior size]

1. absent (Fig. 3B).
2. markedly reduced.
3. not markedly reduced (Fig. 3F).

95. Dorsal notopodial ligule (divided into branchiae or not) [NEXUS: dorsal notopodial branchiae]

1. divided into numerous branchial filaments (Fig. 3H).
2. not divided into numerous branchial filaments.

Only in *Dendronereides* is the dorsal notopodial ligule divided into numerous branchial filaments. The branchial structures of *Dendronereides* and *Dendronereis* are therefore not homologous.

96. Prechaetal notopodial lobe (Fig. 3B, F) [NEXUS: prechaetal notopodial lobe]

1. present.
2. absent.

The prechaetal notopodial lobe is here defined as a digitiform process that is anterior to the acicular notopodial lobe and is not supported by the notopodial acicula (see character 100 Notopodial acicular process).

97. Prechaetal notopodial lobe, development [NEXUS: prechaetal notopodial lobe size]

1. smaller than dorsal notopodial ligule on anterior chaetigers, usually reduced or absent posteriorly.
2. approximately equal to length of dorsal notopodial ligule at least on anterior chaetigers (thus notopodium of three similar sized ligules/lobes).

98. Prechaetal notopodial lobe distribution [NEXUS: prechaetal noto lobe location]

1. present on all chaetigers (may be reduced in size on posterior chaetigers).
2. restricted to a limited number of anterior chaetigers.

99. Prechaetal notopodial lobe, reducing in size posteriorly, last present at approx. chaetiger

100. Notopodial acicular process (Fig. 3E open arrow) [NEXUS: notopodial acicular process]

1. present.
2. absent.

The notopodial acicular process, if present, is a small digitiform process formed around the tip of the acicula and is located between the acicular and ventral notopodial ligules (see character 97 Prechaetal notopodial lobe development).

101. Notopodial acicular process reducing in size posteriorly, last present on chaetiger

102. Notopodial acicular ligule (Fig. 3E–G) [NEXUS: acicular notopodial ligule]

1. present.
2. absent.

The acicular notopodial ligule is here considered to be that fleshy ligule ventral to the acicula in the notopodium. It is present in Nereidinae, but absent in *Ceratocephale*, *Gymnonereis*, *Micronereis* and *Stenoninereis*. Males of some species of *Micronereis* have a process on the ventral side of the notopodial acicular lobe; this dimorphic character is here considered not homologous with the ventral notopodial ligule of most nereidids.

103. Acicular notopodial ligule development [NEXUS: acicular notopodial ligule form]

1. similar to or shorter than neuropodial acicular ligule.
2. prolonged, distinctly longer than neuropodial acicular ligule.
3. reduced, much shorter than neuropodial acicular ligule.

104. Dorsal cirrus (divided into branchiae or not) [NEXUS: dorsal cirrus branchiae]

1. divided into numerous branchial filaments (Fig. 3I).
2. not divided into numerous branchial filaments.

Only in *Dendronereis* does the dorsal cirrus form numerous branchial filaments. The branchial structures of *Dendronereides* and *Dendronereis* are therefore not homologous.

105. Dorsal cirrus (Fig. 3B, F) length on chaetiger 10–20 relative to length of acicular notopodial ligule

106. Dorsal cirrus: sub-terminally attached to dorsal notopodial ligule on posterior chaetigers, or not [NEXUS: dorsal cirrus subterminal]

1. sub-terminally attached to dorsal margin of dorsal notopodial ligule on posterior chaetigers (Fig. 3G).
2. not sub-terminally attached to dorsal notopodial ligule on posterior chaetigers.

107. Dorsal cirrus terminally attached to dorsal notopodial ligule on posterior chaetigers, or not [NEXUS: dorsal cirrus terminal]

1. terminally attached to dorsal notopodial ligule on posterior chaetigers.
2. not terminally attached to dorsal notopodial ligule on posterior chaetigers.

108. Dorsal cirrus terminally attached, or not [NEXUS: dorsal cirrus terminal all]

1. terminally attached throughout, so that dorsal notopodial ligule has appearance of a cirrophore for the dorsal cirrus.
2. not terminally attached throughout all chaetigers.

109. Dorsal cirrus (with/without cirrophore) [NEXUS: dorsal cirrophore]

1. simple, lacking basal cirrophore (Fig. 3C, filled arrow).
2. arising from basal cirrophore.

110. Cirrophore of dorsal cirrus length [NEXUS: dorsal cirrophore development]

1. short, at most as long as ventral notopodial ligule.
2. much longer than ventral notopodial ligule (Fig. 3D).

111. Cirrophore of dorsal cirrus enlargement [NEXUS: dorsal cirrophore vascular]

1. enlarged and vascularised (Fig. 3C, filled arrow).
2. not enlarged and vascularised.

112. Cirrophore of dorsal cirrus (expanded and leaflike, or cylindrical) [NEXUS: dorsal cirrophore expanded]

1. expanded and leaflike (Fig. 3C, filled arrow).
2. cylindrical throughout.

113. Cirrophore of dorsal cirrus expanded commencing approx. chaetiger

114. Neuropodial prechaetal lobe [NEXUS: neuropodial prechaetal lobe]

1. present.
2. absent.

Terminology after Hylleberg and Nateewathana (1988); characteristically present in the gymnonereids *Ceratocephale* and *Gymnonereis*. A structure of the same name is described as being present in descriptions (mostly prior to 1988), for example in some species of *Ceratonereis* but we contend that these are misinterpretations.

115. Neuropodial prechaetal lobe present on chaetigers

116. Neuropodial prechaetal lobe development [NEXUS: neuropodial prechaetal lobe form]

1. projecting beyond postchaetal lobe (at least in anterior chaetigers).
2. not projecting beyond the postchaetal lobe.

117. Neuropodial postchaetal lobe (Fig. 3E, filled arrow) [NEXUS: neuropodial postchaetal lobe]

1. present.
2. absent.

118. Neuropodial postchaetal lobe [NEXUS: neuropodial postchaetal lobe length]

1. projecting beyond end of the acicular ligule (Fig. 3E, filled arrow).
2. not projecting beyond end of the acicular ligule.

119. Neuropodial postchaetal lobe distribution [NEXUS: neuropodial postchaetal lobe distribution]

1. present throughout all chaetigers.
2. restricted to anterior chaetigers.

120. Neuropodial postchaetal lobe form [NEXUS: neuropodial postchaetal lobe form]

1. digitiform (Fig. 3E filled arrow).
2. flattened.

121. Neuropodial postchaetal lobe reducing posteriorly, last present on chaetigers

122. Ventral neuropodial ligule of anterior chaetigers [NEXUS: ventral neuropodial ligule anterior]

1. present (Fig. 3E).
2. absent.

123. Ventral neuropodial ligule of anterior chaetigers development [NEXUS: ventral neuropodial ligule anterior length]

1. approx. as long as acicular neuropodial ligule (Fig. 3E).
2. short, up to half length of acicular neuropodial ligule.

124. Ventral neuropodial ligule on posterior chaetigers [NEXUS: ventral neuropodial ligule posterior]

1. present.
2. absent.

125. Ventral neuropodial ligule on posterior chaetigers development [NEXUS: ventral neuropodial ligule posterior length]

1. similar to length of acicular neuropodial ligule.
2. longer than acicular neuropodial ligule.
3. short, up to half length of acicular neuropodial ligule (Fig. 3G).

126. Accessory ventral cirrus (Fig. 3B, open arrow) [NEXUS: accessory ventral cirrus]

1. present (i.e., double ventral cirri).
2. absent.

127. Accessory ventral cirrus commencing chaetiger

128. Relative length of paired ventral cirri [NEXUS: accessory ventral cirri length]

1. superior ventral cirrus of chaetigers 10–20 longer than inferior cirrus (Fig. 3B, D).
2. superior ventral cirrus of chaetigers 10–20 and inferior cirrus similar in length (Fig. 3C).
3. superior ventral cirrus of chaetigers 10–20 shorter than inferior cirrus.

In most *Ceratocephale* the superior cirrus is always the longer of the pair, especially on the first few chaetigers. However, in at least one species, *Ceratocephalepapillata*, the superior cirrus is shorter than the inferior cirrus on anterior-most 10–20 chaetigers.

#### ﻿Aciculae and chaetae – characters 129–178 (Fig. 4A–Q)

129. Notoaciculae on chaetigers 1 and 2 [NEXUS: notoaciculae chaetigers 1 2]

1. present.
2. absent.

Presence of notoaciculae in chaetigers 1 and 2 is difficult to observe and failure to mention this character in published descriptions cannot be taken as evidence of absence. It is necessary to manipulate the parapodia with transmitted light, or, preferably in small specimens, to remove and mount parapodia on slides. In *Ceratonereismirabilis* and related species, notoaciculae of chaetigers 1 and 2 are present, but are short and translucent even though those of subsequent chaetigers are dark and extend to the tip of the acicular ligule. *Namalycastis* and *Namanereis* have notoacicula in chaetigers 1 and 2, although like notoaciculae in remaining chaetigers, they sit just above the neuroaciculae in the upper part of the neuropodium.

130. Notochaetae of chaetigers 3 and 4 [NEXUS: notochaetae chaetigers 3 4]

1. present.
2. absent.

131. Notochaetae: heterogomph spinigers [NEXUS: notochaetae heterogomph spinigers]

1. present.
2. absent.

**Figure 4.**
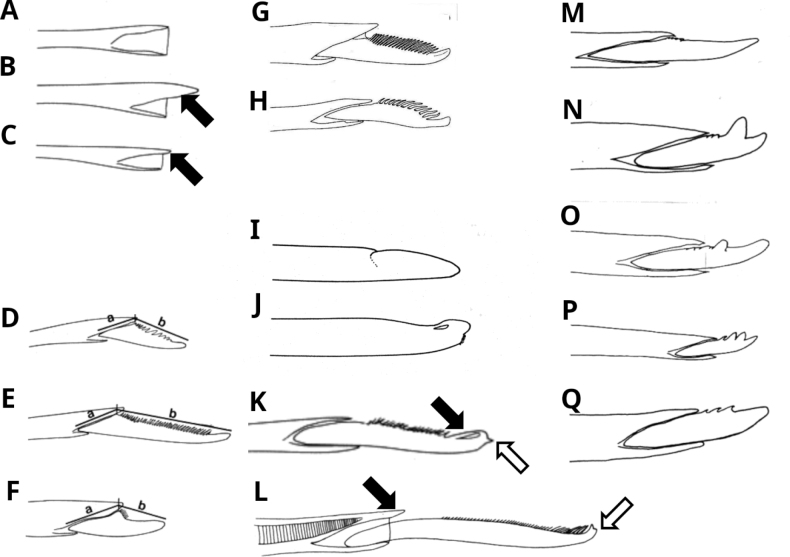
Chaetae **A** chaetal shaft homogomph articulation *Perinereisvallata* spiniger NMV F53971 **B** chaetal shaft heterogomph articulation *Perinereisvallata* falciger NMV F53971 (= long-bossed heterogomph sensu Conde-Vela (2021) filled arrow points to boss **C** chaetal shaft sesquigomph articulation *Ceratonereismirabilis* (= short-bossed heterogomph sensu Conde-Vela (2021) filled arrow points to boss **D** heterogomph falcigers with long blades (a < b; = Type 0 of Bakken and Wilson (2005)) **E** heterogomph falcigers with extra-long blades (2× a < b; = Type 1 of Bakken and Wilson (2005)) **F** heterogomph falcigers with short blades (a ≥ b; = Type 2 of Bakken and Wilson (2005)) **G** heterogomph falciger blade with straight margin **H** heterogomph falciger blade with bowed margin **I** fused heterogomph falciger chaetiger 70 *Hedistediversicolor* complex AHF, Gilleleje, Denmark **J** fused falciger chaetiger 40 *Simplisetiaaequisetis*NMV F53970 **K** homogomph falciger with terminal tendon (filled arrow) and with terminal secondary tooth (open arrow; = bifid) *Platynereisantipoda* notopodial falciger chaetiger 66 NMV F50116 **L** sesquigomph falciger with terminal tendon absent but bifid with secondary terminal tooth (open arrow) and boss (filled arrow) *Ceratonereismirabilis* median chaetiger **M** homogomph falciger with smooth blade *Nereiscirriseta* chaetiger 74 **N** homogomph falcigers with bidentate blade and large adjacent terminal and subterminal teeth *Nereisbifida* chaetiger 71 **O** homogomph falciger with bidentate blade and large widely-separated terminal and subterminal teeth *Nereistriangularis* chaetiger 24 **P** homogomph falciger with multidentate blade with ≥ 2 large lateral teeth, first lateral tooth subequal to terminal tooth, subsequent teeth decreasing in size *Nereisdenhamensis* anterior chaetiger **Q** homogomph falciger with multidentate blade with ≥ 2 small lateral teeth, first and subsequent lateral teeth much smaller than terminal tooth *Nereisapalie* chaetiger 74. Sources: **A–F, I–K, M–J** R. Wilson drawings **G, H** edited after Villalobos-Guerrero et al. (2021: fig. 1e, f) **L** after Perkins (1980: fig. 1d) **M–P** redrawn after Hutchings and Turvey (1982)**Q** after Wilson (1985: fig. 1E). Not to scale; maximum widths of chaetal shafts (at articulation) are in the range 0.01–0.03 mm.

Chaetal shaft with heterogomph articulation is illustrated in Fig. 4B. Equivalent to long-bossed heterogomph sensu Conde-Vela (2021); see character 133 Notochaetae: sesquigomph spinigers. The chaetal shaft boss is the structure indicated with a filled arrow on Fig. 4B, C, L.

132. Notochaetae: homogomph spinigers [NEXUS: notochaetae homogomph spinigers]

1. present.
2. absent.

Chaetal shaft with homogomph articulation is illustrated in Fig. 4A. Equivalent to short-bossed heterogomph sensu Conde-Vela (2021).

133. Notochaetae: sesquigomph spinigers [NEXUS: notochaetae sesquigomph spinigers]

1. present.
2. absent.

Chaetal shaft with sesquigomph articulation is illustrated in Fig. 4C. Despite the advance in quantifying chaetal articulation by Conde-Vela (2021) we retain the terms homogomph/ heterogomph/ sesquigomph due to their near-universal usage in the literature over the near-equivalents proposed by Conde-Vela (2021). It would also be desirable to compare inter- and intra-specific variation between verified Nereididae species before adopting new terms and a revised assessment of their taxonomic significance.

134. Notochaetae: homogomph falcigers (Fig. 4K–Q) [NEXUS: notochaetae homogomph falcigers]

1. present.
2. absent.

135. Notochaetae: homogomph falcigers with terminal tendon (Fig. 4K, filled arrow) [NEXUS: notochaetae homogomph falcigers tendon]

1. present.
2. absent.

136. Notochaetae: homogomph falcigers first present at chaetiger

137. Notochaetae: homogomph falcigers articulation [NEXUS: notochaetae homogomph falcigers articulation]

1. fused on some chaetigers (present as a simple chaeta).
2. with blade free throughout.

138. Notochaetae: homogomph falcigers with smooth blade (Fig. 4M) [NEXUS: notochaetae homogomph falcigers blade smooth]

1. present.
2. absent.

139. Notochaetae: homogomph falcigers with bidentate blade and large adjacent terminal and subterminal teeth (Fig. 4N) [NEXUS: notochaetae homogomph falcigers bidentate]

1. present.
2. absent.

140. Notochaetae: homogomph falcigers with bidentate blade and large widely-separated terminal and subterminal teeth (Fig. 4O) [NEXUS: notochaetae homogomph falcigers bidentate gap]

1. present.
2. absent.

141. Notochaetae: homogomph falcigers with multidentate blade with ≥ 2 large lateral teeth, first lateral tooth subequal to terminal tooth, subsequent teeth usually decreasing in size (Fig. 4P) [NEXUS: notochaetae homogomph falcigers multidentate large]

1. present.
2. absent.

142. Notochaetae: homogomph falcigers with multidentate blade with ≥ 2 small lateral teeth, first and subsequent lateral teeth much smaller than terminal tooth (Fig. 4Q) [NEXUS: notochaetae homogomph falcigers multidentate small]

1. present.
2. absent.

143. Notochaetae: sesquigomph falcigers (Fig. 4L) [NEXUS: notochaetae sesquigomph falcigers]

1. present.
2. absent.

144. Notochaetae: sesquigomph falcigers from chaetiger

145. Notochaetae: sesquigomph falcigers blade form [NEXUS: notochaetae sesquigomph falcigers bifid]

1. distally bifid (Fig. 4K, L open arrows).
2. with a single distal tooth/.

146. Neurochaetae dorsal fascicle: heterogomph spinigers [NEXUS: neurochaetae dorsal fascicle heterogomph spinigers]

1. present.
2. absent.

147. Neurochaetae dorsal fascicle: homogomph spinigers [NEXUS: neurochaetae dorsal fascicle homogomph spinigers]

1. present.
2. absent.

148. Neurochaetae dorsal fascicle: sesquigomph spinigers [NEXUS: neurochaetae dorsal fascicle sesquigomph spinigers]

1. present.
2. absent.

149. Neurochaetae dorsal fascicle: sesquigomph falcigers [NEXUS: neurochaetae dorsal fascicle sesquigomph falcigers]

1. present.
2. absent.

150. Neurochaetae dorsal fascicle: sesquigomph falcigers blades [NEXUS: neurochaetae dorsal fascicle sesquigomph falcigers smooth]

1. serrated.
2. smooth.

151. Neurochaetae dorsal fascicle: heterogomph falcigers in anterior chaetigers [NEXUS: neurochaetae dorsal fascicle heterogomph falcigers anterior]

1. present.
2. absent.

152. Neurochaetae dorsal fascicle: heterogomph falcigers on posterior chaetigers [NEXUS: neurochaetae dorsal fascicle heterogomph falcigers posterior]

1. present.
2. absent.

153. Neurochaetae dorsal fascicle: heterogomph falcigers blades [NEXUS: neurochaetae dorsal fascicle heterogomph falcigers smooth]

1. smooth/
2. serrated/

154. Neurochaetae dorsal fascicle: heterogomph falcigers blades with teeth [NEXUS: neurochaetae dorsal fascicle heterogomph falcigers teeth]

1. only slightly longer proximally than distally.
2. much longer proximally than distally.

155. Neurochaetae dorsal fascicle: heterogomph falcigers blades with number of teeth

156. Neurochaetae dorsal fascicle: simple chaetae (fused falcigers) (Fig. 4I, J) [NEXUS: neurochaetae dorsal fascicle falcigers fused]

1. present.
2. absent.

157. Neurochaetae dorsal fascicle: simple chaetae (fused falcigers) present from chaetiger

158. Neurochaetae dorsal fascicle: homogomph falcigers in anterior chaetigers [NEXUS: neurochaetae dorsal fascicle homogomph falcigers anterior]

1. present.
2. absent.

159. Neurochaetae dorsal fascicle: homogomph falcigers on posterior chaetigers [NEXUS: neurochaetae dorsal fascicle homogomph falcigers posterior]

1. present.
2. absent.

160. Neurochaetae ventral fascicle: sesquigomph falcigers [NEXUS: neurochaetae ventral fascicle sesquigomph falcigers]

1. present.
2. absent.

161. Neurochaetae ventral fascicle: sesquigomph falcigers blade [NEXUS: neurochaetae ventral fascicle sesquigomph falcigers bifid]

1. distally bifid.
2. with a single distal tooth.

162. Neurochaetae ventral fascicle: heterogomph spinigers [NEXUS: neurochaetae ventral fascicle heterogomph spinigers]

1. present.
2. absent.

163. Neurochaetae ventral fascicle: heterogomph spinigers in anterior chaetigers with blades [NEXUS: neurochaetae ventral fascicle heterogomph spinigers anterior serrated]

1. evenly serrated throughout.
2. coarsely serrated proximally.

164. Neurochaetae ventral fascicle: heterogomph spinigers on posterior chaetigers with blades [NEXUS: neurochaetae ventral fascicle heterogomph spinigers posterior serrated] /

1. finely serrated proximally.
2. coarsely serrated proximally.

165. Neurochaetae ventral fascicle: homogomph spinigers [NEXUS: neurochaetae ventral fascicle homogomph spinigers]

1. present.
2. absent.

166. Neurochaetae ventral fascicle: sesquigomph spinigers [NEXUS: neurochaetae ventral fascicle sesquigomph spinigers]

1. present.
2. absent.

167. Neurochaetae ventral fascicle: heterogomph falcigers [NEXUS: neurochaetae ventral fascicle heterogomph falcigers]

1. present.
2. absent.

168. Neurochaetae ventral fascicle: heterogomph falcigers blade [NEXUS: neurochaetae ventral fascicle heterogomph falcigers bowed]

1. tapering, with straight margin (Fig. 4G).
2. bowed, with convex margin (Fig. 4H).

This character was introduced by Villalobos-Guerrero et al. (2022a: fig. 1e, f) and is valuable for distinguishing *Composetia* and similar taxa. Other Nereididae, for example many species illustrated in Pettibone (1971) appear intermediate between bowed and straight-bladed forms or appear to be variable depending on which chaetiger is examined and are difficult to score.

169. Neurochaetae ventral fascicle: anterior chaetigers heterogomph falcigers with long blades (Fig. 4D) [NEXUS: neurochaetae ventral fascicle heterogomph falcigers anterior long]

1. present.
2. absent.

Definitions of blade length of falcigers were introduced by Bakken and Wilson (2005) depending on length of the free margin of the blade relative to the part within the articulation (Fig. 4D–F) but has not been widely adopted. Here we use simpler terms; “long blades” is equivalent to “Type 0” of Bakken and Wilson (2005).

170. Neurochaetae ventral fascicle: anterior chaetigers heterogomph falcigers with extra-long blades (Fig. 4E) [NEXUS: neurochaetae ventral fascicle heterogomph falcigers anterior xlong]

1. present.
2. absent.

The term “extra-long blades” is equivalent to “Type 1” of Bakken and Wilson (2005).

171. Neurochaetae ventral fascicle: anterior chaetigers heterogomph falcigers with short blades (Fig. 4F) [NEXUS: neurochaetae ventral fascicle heterogomph falcigers anterior short]

1. present.
2. absent.

The term “short blades” is equivalent to “Type 2” of Bakken and Wilson (2005).

172. Neurochaetae ventral fascicle: posterior chaetigers heterogomph falcigers with long blades [NEXUS: neurochaetae ventral fascicle heterogomph falcigers posterior long]

1. present.
2. absent.

173. Neurochaetae ventral fascicle: posterior chaetigers heterogomph falcigers with extra-long blades [NEXUS: neurochaetae ventral fascicle heterogomph falcigers posterior xlong]

1. present.
2. absent.

174. Neurochaetae ventral fascicle: posterior chaetigers heterogomph falcigers with short blades [NEXUS: neurochaetae ventral fascicle heterogomph falcigers posterior short]

1. present.
2. absent.

175. Neurochaetae ventral fascicle: heterogomph falcigers blade [NEXUS: neurochaetae ventral fascicle heterogomph falcigers tendon]

1. with recurved terminal tooth and distinct tendon.
2. lacking distinct tendon on terminal tooth.

176. Neurochaetae ventral fascicle: heterogomph falcigers blade [NEXUS: neurochaetae ventral fascicle heterogomph falcigers bifid]

1. terminally bifid.
2. with a single terminal tooth.

177. Neurochaetae ventral fascicle: homogomph falcigers in anterior chaetigers [NEXUS: neurochaetae ventral fascicle homogomph falcigers anterior]

1. present.
2. absent.

178. Neurochaetae ventral fascicle: homogomph falcigers on posterior chaetigers [NEXUS: neurochaetae ventral fascicle homogomph falcigers posterior]

1. present.
2. absent.

#### ﻿Pygidium and appendages – character 179

A trilobate pygidium is present in *Namanereis* while other Namanereidinae, and some *Nicon* species, have a bilobate pygidium. Other nereidids are commonly described as having a funnel-shaped pygidium, which may be crenulated or multi-incised (perhaps indicating specimens approaching epitoky). For the majority of Nereididae the form of the pygidium is unknown, often because specimens were incomplete posteriorly. Thus, we have not included a character describing the form of the pygidium.

179. Anal cirri form [NEXUS: anal cirri form]

1. cirriform or conical.
2. short, stout and appearing as an extension of the pygidium.
3. flattened, resembling posterior dorsal cirri.

#### ﻿Epitokal modifications and reproduction – characters 180–186

Although some epitokal features may be diagnostic at the genus level (Pamungkas and Glasby 2015), they are too poorly known across the family to be used in the present keys. Read (2007), Pamungkas and Glasby (2015) and Conde-Vela et al. (2018) demonstrated their utility in discriminating species across several genera. The seven characters presented below represent the basic characters for documenting epitokal reproductive forms.

180. Dorsal cirrophores of chaetigers 5–7 of epitokes [NEXUS: dorsal cirrophores chaetigers 5 7]

1. unmodified.
2. modified into flattened elytriform discs.
3. modified into spherical globular structures.

181. Natatory region in males commences chaetiger

182. Natatory region in males comprises number of chaetigers

183. Natatory region in females commences chaetiger

184. Natatory region in females comprises number of chaetigers

185. Pygidium of male epitokes [NEXUS: pygidium of male epitokes]

1. unmetamorphosed.
2. with pygidial rosette.

Male epitokes may have the pygidium modified to form a pygidial rosette with multiple rows of short papillae. The unmodified form is illustrated by Villalobos-Guerrero and Bakken (2018: figs 6E, 17L) and the pygidial rosette is illustrated in Villalobos-Guerrero and Bakken (2018: fig. 14C, D).

186. Oocyte shape [NEXUS: oocyte form]

1. spherical.
2. ovoid.

Oocytes are typically spherical in Nereididae but in many *Namanereis* species they are ovoid.

### ﻿Key to genera of Nereididae

It is easy to reach an incorrect identification using a linear (usually dichotomous) key – one always reaches a name, irrespective of errors that may have been made. Thus, it is wise to doubt, and some form of verification is highly desirable. Our recommendation is that after reaching a genus determination using the key below, the next step should be to compare the specimen at hand with the diagnosis of the genus that has been tentatively identified; if specimen and diagnosis match, the user can have increased confidence in the identification. See Methods above for further discussion. As discussed in the Introduction, several Nereididae genera are widely recognised as likely para- or polyphyletic groups. They are polymorphic for characters which distinguish other nereidid genera and therefore key out in more than one couplet.

**Table:** 

1(0)	Maxillary ring paragnaths present (Fig. 1B)	**2**
–	Maxillary ring paragnaths absent (Fig. 1A)	**24**
2(1)	Oral ring paragnaths present (Fig. 1J)	**3**
–	Oral ring paragnaths absent (Fig. 1A)	**13**
3(2)	Dorsal notopodial ligule markedly broader on posterior chaetigers (Fig. 3G)	**4**
–	Dorsal notopodial ligule not markedly broader on posterior chaetigers	**7**
4(3)	Palpophore barrel-shaped, approximately equal width from base to palpostyle (not overly large compared with palpostyle) (Fig. 1A); maxillary ring of pharynx with P-bar paragnaths present, usually in regular comb-like rows (Fig. 2A); Areas VI shield-shaped bars present (Fig. 2G)	***Pseudonereis* Kinberg, 1865**
–	Palpophore massive subconical, flattened (palpostyle is minute by comparison) (Fig. 1B); maxillary ring of pharynx with P-bar paragnaths absent; Area VI shield-shaped bars absent	**5**
5(4)	Ventral peristomial flap present (Fig. 1L); Areas VII-VIII conical paragnaths differentiated, with a separate band of minute paragnaths also present; prechaetal notopodial lobe (Fig. 3B) restricted to a limited number of anterior chaetigers	***Cheilonereis* Benham, 1916**
–	Ventral peristomial flap absent; Areas VII-VIII conical paragnaths similar in size, or irregular mix of large and small paragnaths in a single band; prechaetal notopodial lobe (Fig. 3B) present on all chaetigers	**6**
6(5)	Notochaetae sesquigomph (Fig. 4C) spinigers present; neurochaetae dorsal fascicle heterogomph (Fig. 4B) spinigers present; neurochaetae dorsal fascicle sesquigomph (Fig. 4C) spinigers present	***Nectoneanthes* Imajima, 1972**
–	Notochaetae sesquigomph (Fig. 4C) spinigers absent; neurochaetae dorsal fascicle heterogomph (Fig. 4B) spinigers absent; neurochaetae dorsal fascicle sesquigomph (Fig. 4C) spinigers absent.	***Alitta* Kinberg, 1865**
7(3)	Antennae present; palpostyles present; maxillary ring of pharynx divided into discrete Areas (Fig. 1O, P)	**8**
–	Antennae absent palpostyles absent (palps undivided, minute) maxillary ring of pharynx undivided	***Micronereis* Claparède, 1863**
8(7)	Notochaetae homogomph falcigers (fig. 4M–Q) present	**9**
–	Notochaetae homogomph falcigers absent	**10**
9(8)	Area II rod-like paragnaths present (Fig. 2C); Area III rod-like paragnaths present (Fig. 2C); Area IV conical paragnaths absent	***Platynereis* Kinberg, 1865**
–	Area II rod-like paragnaths absent; Area III rod-like paragnaths absent; Area IV conical paragnaths present (Fig. 2B)	***Nereis* Linnaeus, 1758**
10(8)	Oral ring papillae present; neurochaetae dorsal fascicle heterogomph falcigers in anterior chaetigers absent	***Imajimainereis* de León-González & Solis-Weiss, 2000**
–	Oral ring papillae absent; neurochaetae dorsal fascicle heterogomph falcigers (Fig. 4D–F) in anterior chaetigers present	**11**
11(10)	Area VI smooth bars present	***Perinereis* Kinberg, 1865**
–	Area VI smooth bars absent	**12**
12(11)	Neurochaetae dorsal fascicle simple chaetae (fused falcigers) (Fig. 4I) present	***Hediste* Malmgren, 1867**
–	Neurochaetae dorsal fascicle simple chaetae (fused falcigers) absent	***Neanthes* Kinberg, 1865**
13(2)	Oral ring papillae present	**14**
–	Oral ring papillae absent	**16**
14(13)	Ventral neuropodial ligule on posterior chaetigers similar to length of acicular neuropodial ligule (Fig. 3E)	**15**
–	Ventral neuropodial ligule on posterior chaetigers short, up to half length of acicular neuropodial ligule (Fig. 3F, G)	***Wuinereis* Khlebovich, 1996**
15(14)	Neurochaetae dorsal fascicle homogomph (Fig. 4A) spinigers present	***Leonnates* Kinberg, 1865**
–	Neurochaetae dorsal fascicle homogomph spinigers absent	***Paraleonnates* Khlebovich & Wu, 1962**
16(13)	Notochaetae sesquigomph (Fig. 4C) spinigers present; neurochaetae dorsal fascicle homogomph (Fig. 4A) spinigers absent; prostomium anterior margin indented (Fig. 1G)	**17**
–	Notochaetae sesquigomph spinigers absent; neurochaetae dorsal fascicle homogomph (Fig. 4A) spinigers present; prostomium anterior margin entire (Fig. 1G)	**18**
17(16)	Area II rod-like paragnaths (Fig. 4C) present; notochaetae homogomph (Fig. 4A) falcigers present; Area I conical paragnaths present (Fig. 2B)	***Solomononereis* Gibbs, 1971**
–	Area II rod-like paragnaths absent; notochaetae homogomph falcigers absent; Area I conical paragnaths absent	***Ceratonereis* Kinberg, 1865**
18(16)	Notochaetae homogomph falcigers present	***Nereis* Linnaeus, 1758**
–	Notochaetae homogomph falcigers absent	**9**
19(18)	Neurochaetae dorsal fascicle heterogomph falcigers in anterior chaetigers present (Fig. 4M–Q)	**20**
–	Neurochaetae dorsal fascicle heterogomph falcigers in anterior chaetigers absent	**23**
20(19)	Neurochaetae dorsal fascicle simple chaetae (fused falcigers) (Fig. 4J) present	***Simplisetia* Hartmann-Schröder, 1985**
–	Neurochaetae dorsal fascicle simple chaetae (fused falcigers) absent	**21**
21(20)	Dorsal cirrus terminally attached to dorsal notopodial ligule (Fig. 3C) on posterior chaetigers; neuropodial prechaetal lobe (Fig. 3B) present; neuropodial postchaetal lobe flattened (Fig. 3B)	***Unanereis* Day, 1962**
–	Dorsal cirrus not terminally attached to dorsal notopodial ligule on posterior chaetigers; neuropodial prechaetal lobe absent; neuropodial postchaetal lobe digitiform (Fig. 3F)	**22**
22(21)	Notoaciculae on chaetigers 1 and 2 present	***Potamonereis* Villalobos-Guerrero, Conde-Vela & Sato, 2022**
–	Notoaciculae on chaetigers 1 and 2 absent	***Neanthes* Kinberg, 1865**
23(19)	Palpophore barrel-shaped, approximately equal width from base to palpostyle (not overly large compared with palpostyle) (Fig. 1A); oesophageal caeca present (Fig. 1C); jaws with dentate cutting edge (Fig. 1H)	***Composetia* Hartmann-Schröder, 1985**
–	Palpophore massive subconical, flattened (palpostyle is minute by comparison) (Fig. 1B); oesophageal caeca absent; jaws with smooth or slightly crenulate cutting edge (Fig. 1I)	***Parasetia* Villalobos-Guerrero, Conde-Vela & Sato, 2022**
24(1)	Prostomium anterior margin entire (Fig. 1F)	**25**
–	Prostomium anterior margin indented (Fig. 1G)	**41**
25(24)	Maxillary ring of pharynx with papillae present	**26**
–	Maxillary ring of pharynx with papillae absent	**29**
26(25)	Neurochaetae ventral fascicle sesquigomph (Fig. 4C) spinigers present	**27**
–	Neurochaetae ventral fascicle sesquigomph spinigers absent	**28**
27(26)	Oral ring papillae present; tentacular cirri 3 pairs ventrum of anterior chaetigers smooth (Fig. 1N)	***Lycastonereis* Rao, 1981**
–	Oral ring papillae absent; tentacular cirri 4 pairs; ventrum of anterior chaetigers with rows of tubercles extending to the base of each neuropodium (Fig. 1M)	***Australonereis* Hartman, 1954**
28(26)	Area V papillae present; dorsal notopodial ligule divided into numerous branchial filaments (Fig. 3H); ventral neuropodial ligule of anterior chaetigers absent	***Dendronereides* Southern, 1921**
–	Area V papillae absent dorsal notopodial ligule not divided into numerous branchial filaments; ventral neuropodial ligule (Fig. 3E–G) of anterior chaetigers present	***Olganereis* Hartmann-Schröder, 1977**
29(25)	Dorsal notopodial ligule (Fig. 3E–G) commences chaetiger 1	***Leptonereis* Kinberg, 1865**
–	Dorsal notopodial ligule (Fig. 3E–G) commences chaetiger 3	**30**
–	Dorsal notopodial ligule (Fig. 3E–G) commences chaetiger 4	**38**
–	Dorsal notopodial ligule (Fig. 3E–G) commences chaetiger 5	**40**
30(29)	Notochaetae homogomph falcigers (Fig. 4M–Q) present	**31**
–	Notochaetae homogomph falcigers absent	**33**
31(30)	Oral ring paragnaths (Fig. 1O, P) present	***Eunereis* Malmgren, 1865**
–	Oral ring paragnaths absent	**32**
32(31)	Dorsal notopodial ligule (Fig. 3E) markedly reduced on posterior chaetigers; neurochaetae ventral fascicle heterogomph (Fig. 4B) spinigers absent	***Rullierinereis* Pettibone, 1971**
–	Dorsal notopodial ligule (Fig. 3E) not markedly reduced on posterior chaetigers neurochaetae ventral fascicle heterogomph (Fig. 4B) spinigers present	***Kainonereis* Chamberlin, 1919**
33(30)	Oral ring paragnaths (Fig. 1O, P) present	***Eunereis* Malmgren, 1865**
–	Oral ring paragnaths (Fig. 1O, P) absent	**34**
34(33)	Neuropodial postchaetal lobe (Fig. 3B, E) present	**35**
–	Neuropodial postchaetal lobe absent	**36**
35(34)	Oral ring papillae present	***Websterinereis* Pettibone, 1971**
–	Oral ring papillae absent	***Nicon* Kinberg, 1865**
36(34)	Dorsal notopodial ligule markedly broader on posterior chaetigers (Fig. 3G); dorsal cirrus terminally attached to dorsal notopodial ligule on posterior chaetigers (Fig. 3C); dorsal notopodial ligule markedly elongate on posterior chaetigers (Fig. 3C)	***Leptonereis* Kinberg, 1865**
–	Dorsal notopodial ligule not markedly broader on posterior chaetigers dorsal cirrus not terminally attached to dorsal notopodial ligule on posterior chaetigers dorsal notopodial ligule not markedly elongate on posterior chaetigers	**37**
37(36)	Neurochaetae dorsal fascicle homogomph spinigers (Fig. 4A) present; neurochaetae ventral fascicle sesquigomph (Fig. 4C) spinigers present; palpophore surface with a single transverse groove (palpophores well developed) (Fig. 1A)	***Micronereides* Day, 1963**
–	Neurochaetae dorsal fascicle homogomph (Fig. 4A) spinigers absent; neurochaetae ventral fascicle sesquigomph spinigers (Fig. 4C) absent; palpophore surface without grooves or striae (palps short, compact) (Fig. 4D)	***Namanereis* Chamberlin, 1919**
38(29)	Dorsal notopodial ligule markedly broader on posterior chaetigers (Fig. 3G); dorsal cirrus terminally attached (Fig. 3G) to dorsal notopodial ligule on posterior chaetigers; neuropodial postchaetal lobe absent	***Leptonereis* Kinberg, 1865**
–	Dorsal notopodial ligule not markedly broader on posterior chaetigers (Fig. 3E); dorsal cirrus not terminally attached to dorsal notopodial ligule on posterior chaetigers; neuropodial postchaetal lobe present	**39**
39(38)	Notochaetae homogomph falcigers (Fig. 4A) present; neurochaetae ventral fascicle falcigers blade bowed, with convex margin (Fig. 4H)	***Kainonereis* Chamberlin, 1919**
–	Notochaetae homogomph falcigers absent; neurochaetae ventral fascicle falcigers blade tapering, with straight margin (Fig. 4G)	***Sinonereis* Wu & Sun, 1979**
40(29)	Dorsal notopodial ligule markedly broader on posterior chaetigers (Fig. 3E); dorsal notopodial ligule not markedly reduced on posterior chaetigers; dorsal cirrus terminally attached to dorsal notopodial ligule on posterior chaetigers (Fig. 3C)	***Leptonereis* Kinberg, 1865**
–	Dorsal notopodial ligule not markedly broader on posterior chaetigers; dorsal notopodial ligule markedly reduced on posterior chaetigers; dorsal cirrus not terminally attached to dorsal notopodial ligule on posterior chaetigers	***Typhlonereis* Hansen, 1879**
41(24)	Dorsal cirrus simple, lacking basal cirrophore	**42**
–	Dorsal cirrus arising from basal cirrophore	**46**
42(41)	Ventral neuropodial ligule on posterior chaetigers present (Fig. 3E–G)	**43**
–	Ventral neuropodial ligule on posterior chaetigers absent	**45**
43(42)	Maxillary ring of pharynx with papillae present; dorsal notopodial ligule not markedly reduced on posterior chaetigers (Fig. 3E, F); notoaciculae on chaetigers 1 and 2 absent	**44**
–	Maxillary ring of pharynx with papillae absent; dorsal notopodial ligule markedly reduced on posterior chaetigers notoaciculae on chaetigers 1 and 2 present	***Kinberginereis* Pettibone, 1971**
44(43)	Dorsal notopodial ligule commences chaetiger 1; dorsal notopodial ligule not markedly broader on posterior chaetigers (Fig. 3E, F); maxillary ring of pharynx with papillae in tufts	***Laeonereis* Hartman, 1945**
–	Dorsal notopodial ligule commences chaetiger 3; dorsal notopodial ligule markedly broader on posterior chaetigers (Fig. 3G); maxillary ring of pharynx with papillae solitary	***Tylonereis* Fauvel, 1911**
45(42)	Palpophore barrel-shaped, approximately equal width from base to palpostyle (not overly large compared with palpostyle) (Fig. 1A); notochaetae sesquigomph (Fig. 4C) spinigers present; neurochaetae dorsal fascicle homogomph spinigers absent	***Tylorrhynchus* Grube, 1866**
–	Palpophore massive subconical, flattened (palpostyle is minute by comparison) (Fig. 1B); notochaetae sesquigomph spinigers absent; neurochaetae dorsal fascicle homogomph (Fig. 4A) spinigers present	***Dendronereis* Peters, 1854**
46(41)	Oral ring papillae present notoaciculae on chaetigers 1 and 2 absent; ventral neuropodial ligule of anterior chaetigers present (Fig. 3E–G)	**47**
–	Oral ring papillae absent notoaciculae on chaetigers 1 and 2 present; ventral neuropodial ligule of anterior chaetigers absent	**49**
47(46)	Dorsal notopodial ligule (Fig. 3E) present, commences chaetiger 1	**48**
–	dorsal notopodial ligule absent	***Ceratocephale* Malmgren, 1867**
48(47)	Notochaetae sesquigomph (Fig. 4C) spinigers present; neurochaetae ventral fascicle sesquigomph (Fig. 4C) spinigers present; neurochaetae dorsal fascicle sesquigomph (Fig. 4C) spinigers present	***Gymnonereis* Horst, 1919**
–	Notochaetae sesquigomph spinigers absent; neurochaetae ventral fascicle sesquigomph spinigers absent; neurochaetae dorsal fascicle sesquigomph spinigers absent	***Tambalagamia* Pillai, 1961**
49(46)	Neuropodial postchaetal lobe (Fig. 3B, F) present; antennae form cirriform (usually extending to or past palpophore) (Fig. 1G); palpophore surface with a single transverse groove (palpophores well developed) (Fig. 1A)	***Stenoninereis* Wesenberg-Lund, 1958**
–	Neuropodial postchaetal lobe absent antennae form subconical (usually shorter than palpophore) (Fig. 1F); palpophore surface without grooves or striae (palps short, compact) (Fig. 1D)	***Namalycastis* Hartman, 1959**

## ﻿Systematic account of Nereididae genera

### ﻿ Alitta

Taxon classificationAnimaliaPhyllodocidaNereididae

Kinberg, 1865

4014296E-D3B4-5F7C-AE98-5B931F1C5C2D

Nereis (Alitta) auctt.

#### Type species.

*Nereisvirens* Sars, 1835.

#### WoRMS URL.

https://www.marinespecies.org/polychaeta/aphia.php?p=taxdetails&id=234848.

#### Sources.

Villalobos-Guerrero and Carrera-Parra (2015); Villalobos-Guerrero and Bakken (2018).

#### Diagnosis.

Dorsal notopodial ligule markedly broader on posterior chaetigers; palpophore massive subconical, flattened (palpostyle is minute by comparison); ventral peristomial flap absent; notochaetae sesquigomph spinigers absent (minimal diagnosis; secondary diagnosis not attained).

#### Description.

Palpophore massive subconical, flattened (palpostyle is minute by comparison). Palpophore surface with a single transverse groove (palpophores well developed) or with several oblique grooves or striae (palpophores well developed); palpostyles subconical. Prostomium anterior margin entire. Tentacular belt greater than length of chaetiger 1. Tentacular cirri cirrophores present.

Jaws with dentate cutting edge.

Maxillary ring of pharynx with papillae absent. Maxillary ring paragnaths present. Area I conical paragnaths present (absent occasionally in some specimens of *A.virens* species complex); II conical paragnaths present; III conical paragnaths present; III rod-like paragnaths absent; IV paragnaths present; IV conical paragnaths present; IV smooth bar-like paragnaths absent. Oral ring papillae absent. Oral ring paragnaths present; with Areas V, VI, and VII-VIII discrete. Oral ring pyramidal paragnaths absent, or present. Area V conical paragnaths present, or absent; arranged in a longitudinal line, or irregularly. Area VI paragnaths present; paragnaths arranged in a roughly circular group, or in lines or arcs; conical paragnaths present. Areas VII-VIII paragnaths present; conical paragnaths present; P-bar paragnaths absent, or present.

Dorsal notopodial ligule markedly elongate on posterior chaetigers, or not markedly elongate on posterior chaetigers; markedly broader on posterior chaetigers; not markedly reduced on posterior chaetigers. Prechaetal notopodial lobe present; smaller than dorsal notopodial ligule on anterior chaetigers, usually reduced or absent posteriorly, or approximately equal to length of dorsal notopodial ligule at least on anterior chaetigers (thus notopodium of three similar sized ligules/lobes); present on all chaetigers. Notopodial acicular process absent. Dorsal cirrus sub-terminally attached to dorsal margin of dorsal notopodial ligule on posterior chaetigers; not terminally attached to dorsal notopodial ligule on posterior chaetigers.

Neuropodial prechaetal lobe absent. Neuropodial postchaetal lobe present; projecting beyond end of the acicular ligule; present throughout all chaetigers; digitiform. Ventral neuropodial ligule of anterior chaetigers present. Ventral neuropodial ligule of anterior chaetigers approx. as long as acicular neuropodial ligule. Ventral neuropodial ligule on posterior chaetigers present. Ventral neuropodial ligule on posterior chaetigers similar to length of acicular neuropodial ligule, or short, up to half length of acicular neuropodial ligule.

Notoaciculae on chaetigers 1 and 2 present, or absent. Notochaetae of chaetigers 3 and 4 present. Notochaetae: homogomph spinigers present. Neurochaetae dorsal fascicle: heterogomph spinigers absent; homogomph spinigers present; dorsal fascicle heterogomph falcigers in anterior chaetigers present; on posterior chaetigers present, or absent. Neurochaetae ventral fascicle: heterogomph spinigers present; spinigers in anterior chaetigers with blades evenly serrated throughout; on posterior chaetigers with blades finely serrated proximally; heterogomph falcigers present; anterior chaetigers heterogomph falcigers with long blades absent; anterior chaetigers heterogomph falcigers with extra-long blades present; anterior chaetigers heterogomph falcigers with short blades absent; posterior chaetigers heterogomph falcigers with long blades absent; posterior chaetigers heterogomph falcigers with extra-long blades present; posterior chaetigers heterogomph falcigers with short blades absent; ventral fascicle heterogomph falcigers blade with recurved terminal tooth and distinct tendon, or lacking distinct tendon on terminal tooth; ventral fascicle heterogomph falcigers blade with a single terminal tooth.

Anal cirri form cirriform or conical.

#### Remarks.

The modern concept of *Alitta* is due to Khlebovich (1996) but the generic description and the species included have been emended by Villalobos-Guerrero and Carrera-Parra (2015) and Villalobos-Guerrero and Bakken (2018); the description here is based on that of the latter two studies. *Alitta* now contains eight species all occurring in either the North Pacific or North Atlantic Oceans. *Alittasuccinea* (Leuckart, 1847) has been reported as a supposed introduced species from numerous cosmopolitan localities but as summarised by Villalobos-Guerrero and Carrera-Parra (2015: 165–166) many of these represent misidentifications of related species.

There is no identification guide for all species of *Alitta* but the four species in the *A.virens* species complex can be identified using the keys to atokes and to epitokes in Villalobos-Guerrero and Bakken (2018).

### ﻿ Australonereis

Taxon classificationAnimaliaPhyllodocidaNereididae

Hartman, 1954

8974DE78-DD2F-5644-B6AF-320764DC6F52

#### Type species.

Nereis (Leonnates) ehlersi Augener, 1913.

#### WoRMS URL.

https://www.marinespecies.org/polychaeta/aphia.php?p=taxdetails&id=324844.

#### Sources.

Hutchings and Reid (1990).

#### Diagnosis.

Ventrum of anterior chaetigers with rows of tubercles extending to the base of each neuropodium (minimal diagnosis). Dorsal notopodial ligule commences chaetiger 1; prostomium anterior margin entire; maxillary ring of pharynx with papillae present (secondary diagnosis).

#### Description.

Palpophore barrel-shaped, approximately equal width from base to palpostyle (not overly large compared with palpostyle). Ventrum of anterior chaetigers with rows of tubercles extending to the base of each neuropodium.

Maxillary ring of pharynx with papillae present (sometimes with horny tips); solitary; 50–110 papillae in total. Maxillary ring paragnaths absent. Oral ring papillae absent. Oral ring paragnaths absent.

Dorsal notopodial ligule present; commences chaetiger 1; not markedly elongate on posterior chaetigers; not markedly broader on posterior chaetigers; not markedly reduced on posterior chaetigers. Prechaetal notopodial lobe present; smaller than dorsal notopodial ligule on anterior chaetigers, usually reduced or absent posteriorly; restricted to a limited number of anterior chaetigers. Notopodial acicular process absent. Dorsal cirrus not sub-terminally attached to dorsal notopodial ligule on posterior chaetigers; not terminally attached to dorsal notopodial ligule on posterior chaetigers; not terminally attached throughout all chaetigers.

Neuropodial prechaetal lobe absent. Neuropodial postchaetal lobe present; projecting beyond end of the acicular ligule; digitiform. Ventral neuropodial ligule of anterior chaetigers present.

Notoaciculae on chaetigers 1 and 2 absent. Notochaetae: homogomph spinigers present. Neurochaetae dorsal fascicle: heterogomph spinigers absent; homogomph spinigers present; sesquigomph spinigers present. Neurochaetae dorsal fascicle: sesquigomph falcigers present; blades serrated; heterogomph falcigers in anterior chaetigers absent; on posterior chaetigers absent. Neurochaetae ventral fascicle: sesquigomph falcigers present; heterogomph spinigers absent; homogomph spinigers absent; sesquigomph spinigers present; heterogomph falcigers absent.

Anal cirri form cirriform or conical.

#### Remarks.

*Australonereis* is a monotypic genus. The single species *A.ehlersi* (Augener, 1913) occurs only in southern Australian estuaries where these large and often locally abundant worms are instantly recognisable by the conspicuous rows of tuberculae on the ventral surface; living specimens are also much more fragile than those belonging to other genera of Nereididae and readily fragment in the field.

### ﻿ Ceratocephale

Taxon classificationAnimaliaPhyllodocidaNereididae

Malmgren, 1867

749B279B-4CAE-58B9-B050-FACE11B61DB7

Chaunorhychus Chamberlin, 1919.
Pisionura Hartman & Fauchald, 1971.

#### Type species.

*Ceratocephaleloveni* Malmgren, 1867.

#### WoRMS URL.

https://www.marinespecies.org/polychaeta/aphia.php?p=taxdetails&id=129371.

#### Sources.

Hylleberg and Nateewathana (1988); Hutchings and Reid (1990: table 9); Santos (2007).

#### Diagnosis.

Transverse dorsal lamellae present; dorsal notopodial ligule absent (minimal diagnosis). Dorsal cirrus arising from basal cirrophore; dorsal notopodial ligule commences chaetiger 3 (secondary diagnosis).

#### Description.

Palpophore barrel-shaped, approximately equal width from base to palpostyle (not overly large compared with palpostyle). Palpostyles subconical, or acutely conical. Eyes present, or absent. Prostomium anterior margin indented. Tentacular belt equal to or less than length of chaetiger 1.

Jaws with dentate cutting edge.

Maxillary ring of pharynx with papillae absent. Maxillary ring paragnaths absent. Oral ring papillae present. Oral ring papillae arrangement solitary. Area V three papillae present (close together); VI papillae present, or absent; VII-VIII seven papillae present, arranged in a single row. Oral ring paragnaths absent. In *Ceratocephale* spp., Areas V and VI contain up to three papillae in total; these are here interpreted as all occurring in Area V, with VI = 0,0.

Transverse dorsal lamellae present (in all species except *C.abyssorum*); commencing chaetiger 4–10.

Dorsal notopodial ligule absent. Prechaetal notopodial lobe present; present on all chaetigers. Notopodial acicular process absent. Dorsal cirrus arising from basal cirrophore; cirrophore of dorsal cirrus enlarged and vascularised; cirrophore of dorsal cirrus expanded and leaflike.

Neuropodial postchaetal lobe present; projecting beyond end of the acicular ligule; present throughout all chaetigers; digitiform. Ventral neuropodial ligule of anterior chaetigers present. Ventral neuropodial ligule of anterior chaetigers approx. as long as acicular neuropodial ligule, or short, up to half length of acicular neuropodial ligule. Ventral neuropodial ligule on posterior chaetigers present. Ventral neuropodial ligule on posterior chaetigers short, up to half length of acicular neuropodial ligule. Accessory ventral cirrus present; commencing chaetiger 1–3. Conspicuous neuropodial prechaetal ligule present.

Notoaciculae on chaetigers 1 and 2 absent. Notochaetae: homogomph spinigers present; sesquigomph spinigers present, or absent. Neurochaetae dorsal fascicle: heterogomph spinigers absent; homogomph spinigers present; sesquigomph spinigers present; heterogomph falcigers in anterior chaetigers absent; on posterior chaetigers absent. Neurochaetae ventral fascicle: heterogomph spinigers absent; homogomph spinigers present, or absent; heterogomph falcigers absent.

Anal cirri form cirriform or conical.

#### Remarks.

The definition of *Ceratocephale* used here follows Santos (2007). Bakken et al. (2022) noted the possible synonymy of *Tambalagamia* Pillai, 1961 and suggested that a revision was required. Further evidence of morphological diversity of key characters also suggests that *Ceratocephale* as presently constituted may not be a natural group: according to Santos et al. (2005), in *Ceratocephale* the tentacular belt is shorter than the subsequent chaetigers however this is not so for all species: e.g. *Ceratocephalepapillata* de León-González & Góngora-Garza, 1992, nor in *C.loveni* Malmgren, 1867 or *C.pacifica* (Hartman, 1960) based on Hilbig (1997); and *C.bansei* Khlebovich, 1966 may be the only species of *Ceratocephale* with papillae on Area VI of the pharynx.

*Ceratocephale* currently includes 12 accepted species recorded from all oceans except the Arctic Ocean and from intertidal to abyssal depths (Read and Fauchald 2023). There is no identification guide to all species although Hylleberg and Nateewathana (1988) have a key to the six species then known. Hutchings and Reid (1990) allow identification of the three Australian species currently described although we know of two additional undescribed Australian *Ceratocephale* species.

### ﻿ Ceratonereis

Taxon classificationAnimaliaPhyllodocidaNereididae

Kinberg, 1865

2396D0E1-7485-59B7-A92E-5BAD58B35438

Ceratonereis (Ceratonereis) auctt.

#### Type species.

*Ceratonereismirabilis* Kinberg, 1865.

#### WoRMS URL.

https://www.marinespecies.org/polychaeta/aphia.php?p=taxdetails&id=129372.

#### Sources.

Hartmann-Schröder (1985).

#### Diagnosis.

Notochaetae: sesquigomph falcigers present; dorsal cirrus sub-terminally attached to dorsal margin of dorsal notopodial ligule on posterior chaetigers (minimal diagnosis). Neurochaetae dorsal fascicle: sesquigomph falcigers present; palpophore barrel-shaped, approximately equal width from base to palpostyle (not overly large compared with palpostyle) (secondary diagnosis).

#### Description.

Palpophore barrel-shaped, approximately equal width from base to palpostyle (not overly large compared with palpostyle) (elongate). Palpophore surface with a single transverse groove (palpophores well developed). Prostomium anterior margin indented.

Oesophageal caeca absent.

Jaws with dentate cutting edge.

Maxillary ring of pharynx with papillae absent. Area I conical paragnaths absent; II conical paragnaths present; III conical paragnaths present; IV paragnaths present; IV conical paragnaths present. Oral ring papillae absent. Oral ring paragnaths absent.

Dorsal notopodial ligule not markedly elongate on posterior chaetigers; not markedly broader on posterior chaetigers; markedly reduced on posterior chaetigers, or not markedly reduced on posterior chaetigers. Prechaetal notopodial lobe absent. Notopodial acicular process absent. Dorsal cirrus sub-terminally attached to dorsal margin of dorsal notopodial ligule on posterior chaetigers; arising from basal cirrophore; cirrophore of dorsal cirrus short, at most as long as ventral notopodial ligule, or much longer than ventral notopodial ligule; cirrophore of dorsal cirrus not enlarged and vascularised; cirrophore of dorsal cirrus cylindrical throughout.

Neuropodial postchaetal lobe present; projecting beyond end of the acicular ligule; restricted to anterior chaetigers; digitiform or flattened. Ventral neuropodial ligule of anterior chaetigers present. Ventral neuropodial ligule of anterior chaetigers approx. as long as acicular neuropodial ligule, or short, up to half length of acicular neuropodial ligule. Ventral neuropodial ligule on posterior chaetigers present. Ventral neuropodial ligule on posterior chaetigers similar to length of acicular neuropodial ligule, or short, up to half length of acicular neuropodial ligule.

Notoaciculae on chaetigers 1 and 2 present. Notochaetae: homogomph spinigers absent; sesquigomph spinigers present; sesquigomph falcigers present; blade distally bifid, or with a single distal tooth. Neurochaetae dorsal fascicle: heterogomph spinigers absent; homogomph spinigers absent; sesquigomph spinigers present; sesquigomph falcigers present; heterogomph falcigers in anterior chaetigers present. Neurochaetae ventral fascicle: heterogomph spinigers present; homogomph spinigers absent; heterogomph falcigers present; anterior chaetigers heterogomph falcigers with short blades absent; heterogomph falcigers blade lacking distinct tendon on terminal tooth; heterogomph falcigers blade terminally bifid, or with a single terminal tooth.

Anal cirri form cirriform or conical.

#### Remarks.

*Ceratonereis* as currently defined follows the concept of Perkins (1980) and Hartmann-Schröder (1985). Previously the genus (“*Ceratonereis* sensu lato”) had included unrelated nereidid species with superficially similar paragnath configuration. Those disparate species (none of which have the indented prostomium characteristic of *Ceratonereis* sensu stricto) are now moved to genera *Composetia*Hartmann-Schröder (1985) and *Simplisetia*Hartmann-Schröder (1985). This restricted definition of *Ceratonereis* is probably monophyletic (Bakken and Wilson 2005) and comprises 43 species which are known from all oceans (Read and Fauchald 2023).

There is no identification guide to the species of *Ceratonereis* and many nominal species are poorly known and some still may belong to other genera. Distinguishing species relies heavily on differences in chaetae and in parapodial structures; pigmentation pattern in living specimens is often distinctive and would probably be most helpful.

Conde-Vela (2021) includes a key to American species of *Ceratonereis*. Glasby (2015) provides a key that includes four species of *Ceratonereis* known from tropical Australia.

### ﻿ Cheilonereis

Taxon classificationAnimaliaPhyllodocidaNereididae

Benham, 1916

16CE8BC8-12DD-5269-A64D-F44A30B15F32

#### Type species.

*Nereiscyclurus* Harrington, 1897.

#### WoRMS URL.

https://www.marinespecies.org/polychaeta/aphia.php?p=taxdetails&id=156851.

#### Sources.

Bakken et al. (2022).

#### Diagnosis.

Ventral peristomial flap present (minimal diagnosis). Areas VII-VIII conical paragnaths differentiated, with a separate band of minute paragnaths also present; dorsal notopodial ligule markedly broader on posterior chaetigers (secondary diagnosis).

#### Description.

Palpophore massive subconical, flattened (palpostyle is minute by comparison). Tentacular belt greater than length of chaetiger 1. Ventral peristomial flap present.

Oesophageal caeca present.

Jaws with dentate cutting edge.

Maxillary ring of pharynx with papillae absent. Maxillary ring paragnaths present. Area I conical paragnaths present; II conical paragnaths present; IV paragnaths present; IV conical paragnaths present. Oral ring papillae absent. Oral ring paragnaths present; with Areas V, VI and VII-VIII discrete; on Area V and VI form distinct groups. Area V conical paragnaths absent. Area VI paragnaths present; paragnaths arranged in a roughly circular group; conical paragnaths present; smooth bars absent. Areas VII-VIII paragnaths present; conical; arranged in isolated patches, or in one or more irregular lines forming a continuous band; conical paragnaths differentiated, with a separate band of minute paragnaths also present (present as patches in *C.peristomialis*).

Dorsal notopodial ligule markedly elongate on posterior chaetigers; markedly broader on posterior chaetigers; not markedly reduced on posterior chaetigers. Prechaetal notopodial lobe present; smaller than dorsal notopodial ligule on anterior chaetigers, usually reduced or absent posteriorly; restricted to a limited number of anterior chaetigers. Dorsal cirrus sub-terminally attached to dorsal margin of dorsal notopodial ligule on posterior chaetigers.

Neuropodial postchaetal lobe absent, or present; projecting beyond end of the acicular ligule; present throughout all chaetigers; flattened. Ventral neuropodial ligule of anterior chaetigers present. Ventral neuropodial ligule of anterior chaetigers approx. as long as acicular neuropodial ligule. Ventral neuropodial ligule on posterior chaetigers present. Ventral neuropodial ligule on posterior chaetigers similar to length of acicular neuropodial ligule.

Notoaciculae on chaetigers 1 and 2 absent. Notochaetae: homogomph spinigers present; homogomph falcigers present; homogomph falcigers with multidentate blade with two or more small lateral teeth, first and subsequent lateral teeth much smaller than terminal tooth present. Neurochaetae dorsal fascicle: heterogomph spinigers absent; homogomph spinigers present; heterogomph falcigers in anterior chaetigers present; on posterior chaetigers present; blades serrated. Neurochaetae ventral fascicle: heterogomph spinigers present; homogomph spinigers absent; heterogomph falcigers present; anterior chaetigers heterogomph falcigers with long blades absent; anterior chaetigers heterogomph falcigers with extra-long blades present; anterior chaetigers heterogomph falcigers with short blades absent; posterior chaetigers heterogomph falcigers with long blades present; posterior chaetigers heterogomph falcigers with extra-long blades absent; posterior chaetigers heterogomph falcigers with short blades absent; heterogomph falcigers blade lacking distinct tendon on terminal tooth.

Anal cirri form cirriform or conical.

#### Remarks.

The genus *Cheilonereis* has two species, *C.cyclurus* (Harrington, 1897) from the NW Pacific and *C.peristomialis* Benham, 1916 from the SE Pacific; both species are commensals of hermit crabs. *Cheilonereis* species are characterised by the presence of a ventral collar or flap that partly covers the ventral paragnaths of the oral ring when the pharynx is extended (see photo by Dave Cowles, Walla Walla University: https://inverts.wallawalla.edu/Annelida/Nereidae/Cheilonereis_cyclurus_DLC2018-13.jpg also included as part of Wilson et al. (2023)); this structure is unknown in other Nereididae and it is plausible that the ventral flap is adaptive in some way for their commensal life style.

### ﻿ Composetia

Taxon classificationAnimaliaPhyllodocidaNereididae

Hartmann-Schröder, 1985

9E81B4FF-710B-5DCB-8D28-40F423FDF928

Ceratonereis (Composetia) Hartmann-Schröder, 1985.

#### Type species.

*Nereiscostae* Grube, 1840.

#### WoRMS URL.

https://www.marinespecies.org/polychaeta/aphia.php?p=taxdetails&id=324848.

#### Sources.

Villalobos-Guerrero et al. (2022a).

#### Diagnosis.

Neurochaetae ventral fascicle homogomph spinigers present; maxillary ring paragnaths present; oral ring paragnaths absent; Neurochaetae dorsal fascicle: heterogomph falcigers in anterior chaetigers absent; oesophageal caeca present (minimal diagnosis; secondary diagnosis not attained).

#### Description.

Palpophore barrel-shaped, approximately equal width from base to palpostyle (not overly large compared with palpostyle). Palpophore surface with a single transverse groove (palpophores well developed). Prostomium longitudinal groove present; anterior region entire, sub-quadrangular, longitudinal groove present; prostomial posterior region subequal to or longer than anterior region. Tentacular belt greater than length of chaetiger 1.

Oesophageal caeca present.

Jaws with dentate cutting edge.

Maxillary ring of pharynx with papillae absent. Maxillary ring paragnaths present. Area I conical paragnaths present; II conical paragnaths present; III conical paragnaths present; IV paragnaths present; IV conical paragnaths present. Oral ring papillae absent. Oral ring paragnaths absent.

Dorsal notopodial ligule not markedly broader on posterior chaetigers; not markedly reduced on posterior chaetigers. Prechaetal notopodial lobe present; smaller than dorsal notopodial ligule on anterior chaetigers, usually reduced or absent posteriorly; present on all chaetigers, or restricted to a limited number of anterior chaetigers. Notopodial acicular process absent. Dorsal cirrus not sub-terminally attached to dorsal notopodial ligule on posterior chaetigers.

Neuropodial postchaetal lobe present; projecting beyond end of the acicular ligule; restricted to anterior chaetigers. Ventral neuropodial ligule of anterior chaetigers present. Ventral neuropodial ligule of anterior chaetigers approx. as long as acicular neuropodial ligule. Ventral neuropodial ligule on posterior chaetigers present. Ventral neuropodial ligule on posterior chaetigers similar to length of acicular neuropodial ligule.

Notoaciculae on chaetigers 1 and 2 absent. Notochaetae: homogomph spinigers present. Neurochaetae dorsal fascicle: heterogomph spinigers absent; homogomph spinigers present; heterogomph falcigers in anterior chaetigers absent; on posterior chaetigers absent. Neurochaetae ventral fascicle: heterogomph spinigers absent; homogomph spinigers present; heterogomph falcigers present; falcigers blade tapering, with straight margin; anterior chaetigers heterogomph falcigers with long blades absent; anterior chaetigers heterogomph falcigers with extra-long blades present; anterior chaetigers heterogomph falcigers with short blades absent; posterior chaetigers heterogomph falcigers with long blades absent; posterior chaetigers heterogomph falcigers with extra-long blades present; posterior chaetigers heterogomph falcigers with short blades absent; heterogomph falcigers blade lacking distinct tendon on terminal tooth; homogomph falcigers on posterior chaetigers present, or absent.

Anal cirri form cirriform or conical.

#### Remarks.

The description here follows the revised concept of *Composetia* of Villalobos-Guerrero et al. (2022a) who removed several former species of *Composetia* sensu Hartmann-Schröder, 1985 to two new genera: *Parasetia* and *Potamonereis*. *Composetia* currently includes 11 species which collectively are widely distributed around the globe; however, many species have not yet been re-evaluated against the revised concept of Villalobos-Guerrero et al. (2022a) the genus remains as an assemblage of dissimilar species Villalobos-Guerrero et al. (2022a).

The only identification guide is the tabular comparisons of Villalobos-Guerrero et al. (2022a).

### ﻿ Dendronereides

Taxon classificationAnimaliaPhyllodocidaNereididae

Southern, 1921

DF13E0A3-B540-593F-9BB4-3AAC46C1AD2E

#### Type species.

*Dendronereidesheteropoda* Southern, 1921.

#### WoRMS URL.

https://www.marinespecies.org/polychaeta/aphia.php?p=taxdetails&id=206894.

#### Sources.

Southern (1921); Hutchings and Reid (1990).

#### Diagnosis.

Dorsal notopodial ligule divided into numerous branchial filaments (minimal diagnosis).

Ventral neuropodial ligule of anterior chaetigers absent; maxillary ring of pharynx with papillae present; prostomium anterior margin entire (secondary diagnosis).

#### Description.

Palpophore barrel-shaped, approximately equal width from base to palpostyle (not overly large compared with palpostyle). Palpophore surface with a single transverse groove (palpophores well developed).

Jaws with dentate cutting edge.

Maxillary ring of pharynx with papillae present; solitary. Maxillary ring paragnaths absent. Oral ring papillae present. Oral ring papillae arrangement solitary. Area V papillae present; VI papillae present; VII-VIII papillae present. Oral ring paragnaths absent.

Dorsal notopodial ligule present. Dorsal notopodial ligule divided into numerous branchial filaments. Dorsal cirrus not sub-terminally attached to dorsal notopodial ligule on posterior chaetigers; not terminally attached throughout all chaetigers.

Ventral neuropodial ligule of anterior chaetigers absent. Ventral neuropodial ligule on posterior chaetigers absent.

Notochaetae: homogomph spinigers present; sesquigomph spinigers present, or absent. Neurochaetae dorsal fascicle: heterogomph spinigers present, or absent; homogomph spinigers present; sesquigomph spinigers present, or absent; heterogomph falcigers in anterior chaetigers present, or absent; homogomph falcigers in anterior chaetigers present, or absent. Neurochaetae ventral fascicle: heterogomph spinigers present, or absent; homogomph spinigers present, or absent; heterogomph falcigers present, or absent; heterogomph falcigers blade lacking distinct tendon on terminal tooth; homogomph falcigers on posterior chaetigers present, or absent.

#### Remarks.

*Dendronereides* is the only nereidid genus in which the dorsal notopodial ligule is transformed into “branchial” filaments; the genus contains three species which occur in tropical estuaries of the Indo-Pacific. There is no taxonomic review or identification guide for the species of *Dendronereides*.

### ﻿ Dendronereis

Taxon classificationAnimaliaPhyllodocidaNereididae

Peters, 1854

1A17B1DC-9FC4-5F1C-87C5-87884ACDA302

#### Type species.

*Dendronereisarborifera* Peters, 1854.

#### WoRMS URL.

https://www.marinespecies.org/polychaeta/aphia.php?p=taxdetails&id=206700.

#### Sources.

Hsueh (2019b).

#### Diagnosis.

Dorsal cirrus divided into numerous branchial filaments (minimal diagnosis). Ventral neuropodial ligule on posterior chaetigers absent; palpophore massive subconical, flattened (palpostyle is minute by comparison); prostomium anterior margin indented (secondary diagnosis).

#### Description.

Palpophore massive subconical, flattened (palpostyle is minute by comparison). Prostomium anterior margin indented.

Maxillary ring of pharynx with papillae present, or absent. Maxillary ring paragnaths absent. Oral ring papillae present. Oral ring paragnaths absent.

Dorsal notopodial ligule not markedly elongate on posterior chaetigers; not markedly broader on posterior chaetigers; not markedly reduced on posterior chaetigers. Prechaetal notopodial lobe present; smaller than dorsal notopodial ligule on anterior chaetigers, usually reduced or absent posteriorly. Dorsal cirrus divided into numerous branchial filaments. Dorsal cirrus not sub-terminally attached to dorsal notopodial ligule on posterior chaetigers; not terminally attached to dorsal notopodial ligule on posterior chaetigers; not terminally attached throughout all chaetigers.

Ventral neuropodial ligule of anterior chaetigers present. Ventral neuropodial ligule of anterior chaetigers approx. as long as acicular neuropodial ligule. Ventral neuropodial ligule on posterior chaetigers absent. Neuropodia of branchial chaetigers divided into five lobes, plus ventral cirrus, plus two smaller cirri on ventral neuropodial lobe (not arising from same location as ventral cirri). Posteriorly becoming simpler, eventually with a single neuropodial lobe and ventral cirrus.

Notochaetae: homogomph spinigers present. Neurochaetae dorsal fascicle: heterogomph spinigers absent; homogomph spinigers present; heterogomph falcigers in anterior chaetigers absent; on posterior chaetigers absent. Neurochaetae ventral fascicle: heterogomph spinigers absent; homogomph spinigers present; heterogomph falcigers absent.

#### Remarks.

Species belonging to *Dendronereis* are easily recognised as this is the only genus of Nereididae in which the dorsal cirrus is transformed into branchial filaments. Bakken et al. (2022) incorrectly stated that maxillary ring paragnaths may be present or absent; they are always absent as per the corrected description and diagnosis above.

Five species of *Dendronereis* are known, all occurring in shallow waters of the Indo-Pacific. Hsueh (2019b) provides a key to all known species.

### ﻿ Eunereis

Taxon classificationAnimaliaPhyllodocidaNereididae

Malmgren, 1865

2D3814AB-6658-59B9-99B3-E5B5DDB85B41

Nereis (Eunereis) Malmgren, 1865.

#### Type species.

*Nereislongissima* Johnston, 1840.

#### WoRMS URL.

https://www.marinespecies.org/polychaeta/aphia.php?p=taxdetails&id=129373.

#### Sources.

Hsueh (2018); Bakken et al. (2022).

#### Diagnosis.

Oral ring paragnaths present; maxillary ring paragnaths absent (minimal diagnosis; secondary diagnosis not attained).

#### Description.

Palpophore barrel-shaped, approximately equal width from base to palpostyle (not overly large compared with palpostyle). Palpophore surface with a single transverse groove (palpophores well developed). Tentacular belt greater than length of chaetiger 1.

Jaws with dentate cutting edge.

Maxillary ring of pharynx with papillae absent. Maxillary ring paragnaths absent. Oral ring papillae present, or absent. Oral ring paragnaths present; with Areas V, VI, and VII-VIII discrete, or comprising a continuous ring dorsally and ventrally, discrete groups not recognisable; on Areas V and VI form distinct groups. Area V conical paragnaths present, or absent. Area VI paragnaths present; arranged in a roughly circular group; conical paragnaths present, or absent; smooth bars present, or absent. Areas VII-VIII paragnaths present, or absent; conical paragnaths present; conical paragnaths arranged in isolated patches, or in one or more irregular lines forming a continuous band; conical paragnaths similar in size, or irregular mix of large and small paragnaths in a single band; rod-shaped paragnaths absent.

Dorsal notopodial ligule not markedly elongate on posterior chaetigers; not markedly broader on posterior chaetigers; markedly reduced on posterior chaetigers, or not markedly reduced on posterior chaetigers. Prechaetal notopodial lobe present, or absent; smaller than dorsal notopodial ligule on anterior chaetigers, usually reduced or absent posteriorly; restricted to a limited number of anterior chaetigers. Notopodial acicular process present, or absent. Dorsal cirrus not sub-terminally attached to dorsal notopodial ligule on posterior chaetigers; not terminally attached to dorsal notopodial ligule on posterior chaetigers; not terminally attached throughout all chaetigers; simple, lacking basal cirrophore.

Neuropodial prechaetal lobe absent. Neuropodial postchaetal lobe absent, or present; projecting beyond end of the acicular ligule; restricted to anterior chaetigers; digitiform. Ventral neuropodial ligule of anterior chaetigers present. Ventral neuropodial ligule of anterior chaetigers approx. as long as acicular neuropodial ligule. Ventral neuropodial ligule on posterior chaetigers present. Ventral neuropodial ligule on posterior chaetigers similar to length of acicular neuropodial ligule.

Notoaciculae on chaetigers 1 and 2 absent. Notochaetae: homogomph spinigers present; homogomph falcigers present, or absent. Neurochaetae dorsal fascicle: heterogomph spinigers absent; homogomph spinigers present; heterogomph falcigers in anterior chaetigers present; on posterior chaetigers present; blades serrated. Neurochaetae ventral fascicle: heterogomph spinigers present; homogomph spinigers absent; heterogomph falcigers present; falcigers blade tapering, with straight margin; anterior chaetigers heterogomph falcigers with long blades absent; anterior chaetigers heterogomph falcigers with extra-long blades present; anterior chaetigers heterogomph falcigers with short blades absent; posterior chaetigers heterogomph falcigers with long blades present, or absent; posterior chaetigers heterogomph falcigers with extra-long blades present, or absent; posterior chaetigers heterogomph falcigers with short blades absent; heterogomph falcigers blade lacking distinct tendon on terminal tooth.

Anal cirri form cirriform or conical.

#### Remarks.

The description here follows Hsueh (2018) with additional information on the straight/bowed blade falcigers character introduced (for *Composetia*) by Villalobos-Guerrero et al. (2022a).

*Eunereis* is the only Nereididae genus with paragnaths present only on the oral ring. *Eunereis* includes species which in other respects, especially chaetal characters, are similar to either of the genera *Neanthes* or *Nereis* suggesting a review is necessary.

*Eunereis* includes ten accepted species which encompass wide geographic and bathymetric distributions. There is no published identification guide to all species.

### ﻿ Gymnonereis

Taxon classificationAnimaliaPhyllodocidaNereididae

Horst, 1919

3018967B-9FEF-5D04-B384-F9791C2CB15B

Gymnorhynchus Horst, 1919 (replaced homonym).

#### Type species.

*Gymnorhynchussibogae* Horst, 1918.

#### WoRMS URL.

https://www.marinespecies.org/polychaeta/aphia.php?p=taxdetails&id=324851.

#### Sources.

Pettibone (1970); Hutchings and Reid (1990).

#### Diagnosis.

Accessory ventral cirrus present; neurochaetae ventral fascicle sesquigomph spinigers present (minimal diagnosis). Notochaetae sesquigomph spinigers present; dorsal notopodial ligule present (secondary diagnosis).

#### Description.

Palpophore barrel-shaped, approximately equal width from base to palpostyle (not overly large compared with palpostyle). Palpophore surface with a single transverse groove (palpophores well developed); palpostyles subconical, or acutely conical. Prostomium anterior margin indented.

Jaws with smooth or slightly crenulate cutting edge or with dentate cutting edge.

Maxillary ring of pharynx with papillae absent. Maxillary ring paragnaths absent. Oral ring papillae present. Oral ring papillae arrangement solitary. Area V papillae present; VI papillae present; VII-VIII papillae present. Oral ring paragnaths absent.

Transverse dorsal lamellae absent, or present.

Dorsal notopodial ligule present; commences chaetiger 1; not markedly elongate on posterior chaetigers; not markedly broader on posterior chaetigers; markedly reduced on posterior chaetigers (but cirrophore of dorsal cirrus is expanded and looks like an expanded notopodial lobe unless progressive change is noted over many chaetigers). Prechaetal notopodial lobe present; smaller than dorsal notopodial ligule on anterior chaetigers, usually reduced or absent posteriorly; present on all chaetigers. Notopodial acicular process present. Dorsal cirrus not sub-terminally attached to dorsal notopodial ligule on posterior chaetigers; not terminally attached to dorsal notopodial ligule on posterior chaetigers; not terminally attached throughout all chaetigers; arising from basal cirrophore.

Neuropodial prechaetal lobe present; extending beyond postchaetal lobe (at least in anterior chaetigers). Neuropodial postchaetal lobe present; not projecting beyond end of the acicular ligule; present throughout all chaetigers; flattened. Ventral neuropodial ligule of anterior chaetigers present. Ventral neuropodial ligule of anterior chaetigers approx. as long as acicular neuropodial ligule. Ventral neuropodial ligule on posterior chaetigers present. Ventral neuropodial ligule on posterior chaetigers similar to length of acicular neuropodial ligule, or short, up to half length of acicular neuropodial ligule. Accessory ventral cirrus present.

Notoaciculae on chaetigers 1 and 2 absent. Notochaetae: homogomph spinigers present, or absent; sesquigomph spinigers present. Neurochaetae dorsal fascicle: heterogomph spinigers absent; homogomph spinigers present, or absent; sesquigomph spinigers present. Neurochaetae dorsal fascicle: sesquigomph falcigers present, or absent; blades serrated; heterogomph falcigers in anterior chaetigers absent; on posterior chaetigers absent. Neurochaetae ventral fascicle: sesquigomph falcigers present, or absent; heterogomph spinigers absent; homogomph spinigers present, or absent; sesquigomph spinigers present; heterogomph falcigers absent.

Anal cirri form cirriform or conical.

#### Remarks.

The genus *Gymnonereis* is diagnosed by the presence of accessory dorsal and ventral cirri and notochaetae comprising sesquigomph spinigers (although as discussed by Darbyshire 2014 it is not clear that these chaetae are alike in all species). *Gymnonereis* is most similar to *Tambalagamia* and the two have been combined by some authors, *Gymnonereis* being the senior synonym (Pettibone 1970; Hylleberg and Nateewathana 1988).

*Gymnonereis* is a genus of seven species, predominantly occurring in the southern hemisphere and the tropical Indo-Pacific and from shallow water (~ 60 m or less). There is no published key to all species although several regional keys exist (Hylleberg and Nateewathana 1988; Hutchings and Reid 1990).

### ﻿ Hediste

Taxon classificationAnimaliaPhyllodocidaNereididae

Malmgren, 1867

744BCCC8-1842-5F0C-BC2C-B7C3B5B79A7A

Nereis (Hediste) Malmgren, 1867.

#### Type species.

*Nereisdiversicolor* Müller, 1776.

#### WoRMS URL.

https://www.marinespecies.org/polychaeta/aphia.php?p=taxdetails&id=146968.

#### Sources.

Sato and Nakashima (2003).

#### Diagnosis.

Neurochaetae dorsal fascicle simple chaetae (fused falcigers) present; palpophore massive subconical, flattened (palpostyle is minute by comparison) (minimal diagnosis; secondary diagnosis not attained).

#### Description.

Palpophore massive subconical, flattened (palpostyle is minute by comparison). Palpophore surface with a single transverse groove (palpophores well developed). Tentacular belt greater than length of chaetiger 1.

Jaws with dentate cutting edge.

Maxillary ring of pharynx with papillae absent. Area I conical paragnaths present; II conical paragnaths present; III conical paragnaths present; III rod-like paragnaths absent; IV paragnaths present; IV conical paragnaths present; IV rod-like paragnaths absent. Oral ring papillae absent. Oral ring paragnaths present; with Areas V, VI, and VII-VIII discrete; on Areas V and VI form distinct groups. Area V conical paragnaths absent. Area VI paragnaths present; paragnaths arranged in a roughly circular group, or in lines or arcs; conical paragnaths present; smooth bars absent. Area VII-VIII paragnaths present; conical paragnaths present; conical paragnaths arranged in one or more irregular lines forming a continuous band; conical paragnaths similar in size, or irregular mix of large and small paragnaths in a single band; rod-shaped paragnaths absent.

Dorsal notopodial ligule not markedly elongate on posterior chaetigers; not markedly broader on posterior chaetigers; not markedly reduced on posterior chaetigers. Prechaetal notopodial lobe present; smaller than dorsal notopodial ligule on anterior chaetigers, usually reduced or absent posteriorly; restricted to a limited number of anterior chaetigers. Notopodial acicular process absent. Dorsal cirrus not sub-terminally attached to dorsal notopodial ligule on posterior chaetigers; not terminally attached to dorsal notopodial ligule on posterior chaetigers; not terminally attached throughout all chaetigers.

Neuropodial postchaetal lobe absent, or present; projecting beyond end of the acicular ligule; restricted to anterior chaetigers; digitiform. Ventral neuropodial ligule of anterior chaetigers present. Ventral neuropodial ligule of anterior chaetigers approx. as long as acicular neuropodial ligule. Ventral neuropodial ligule on posterior chaetigers present. Ventral neuropodial ligule on posterior chaetigers similar to length of acicular neuropodial ligule.

Notoaciculae on chaetigers 1 and 2 absent. Notochaetae: homogomph spinigers present. Neurochaetae dorsal fascicle: heterogomph spinigers absent; homogomph spinigers present; heterogomph falcigers in anterior chaetigers present; on posterior chaetigers present; blades serrated; simple chaetae (fused falcigers) present. Neurochaetae ventral fascicle: heterogomph spinigers present or absent; homogomph spinigers absent; homogomph falcigers present or absent; heterogomph falcigers present; anterior chaetigers heterogomph falcigers with long blades absent; anterior chaetigers heterogomph falcigers with extra-long blades present; anterior chaetigers heterogomph falcigers with short blades absent; posterior chaetigers heterogomph falcigers with long blades absent; posterior chaetigers heterogomph falcigers with extra-long blades present; posterior chaetigers heterogomph falcigers with short blades absent; heterogomph falcigers blade lacking distinct tendon on terminal tooth.

Anal cirri form cirriform or conical.

#### Remarks.

*Hediste* species are characterised by the “simple” neuropodial falcigers (with fused articulation) which are present in posterior chaetigers and by having paragnaths on both rings of the pharynx. The most recent taxonomic treatments are Sato and Nakashima (2003) and Teixeira et al. (2022a) who described a total of four new species, broadened the generic diagnosis, and demonstrated the power of morphometric methods to discriminate cryptic species.

*Hediste* is a genus of estuarine nereidids which occur in the northern hemisphere. Seven species of *Hediste* are currently described but despite recent publications, the most widespread species, *H.diversicolor* (O.F. Müller, 1776) still contains cryptic species not yet described (Tosuji et al. 2018; Teixeira et al. 2022a). A key to Asian species of *Hediste* is provided by Sato and Nakashima (2003) and Teixeira et al. (2022a) provide a key to European species.

### ﻿ Imajimainereis

Taxon classificationAnimaliaPhyllodocidaNereididae

de León-González & Solis-Weiss, 2000

D636ADD0-7EEE-5BA6-8616-B66E794475AB

#### Type species.

*Imajimainereispacifica* de León-González & Solís-Weiss, 2000.

#### WoRMS URL.

https://www.marinespecies.org/polychaeta/aphia.php?p=taxdetails&id=325837.

#### Sources.

de León-González and Solís-Weiss (2000).

#### Diagnosis.

Oral ring paragnaths present; oral ring papillae present; neurochaetae dorsal fascicle heterogomph spinigers present (minimal diagnosis). Neurochaetae dorsal fascicle heterogomph falcigers in anterior chaetigers absent; Area VI papillae absent; palpophore barrel-shaped, approximately equal width from base to palpostyle (not overly large compared with palpostyle) (secondary diagnosis).

#### Description.

Palpophore barrel-shaped, approximately equal width from base to palpostyle (not overly large compared with palpostyle). Tentacular belt greater than length of chaetiger 1.

Jaws with dentate cutting edge.

Maxillary ring of pharynx with papillae absent. Area I conical paragnaths present; II conical paragnaths present; III conical paragnaths present; III rod-like paragnaths absent; IV paragnaths present; IV conical paragnaths present; IV rod-like paragnaths absent. Oral ring papillae present. Oral ring papillae arrangement solitary. Area V papillae absent; VI papillae absent; VII-VIII papillae present. Oral ring paragnaths present; with Areas V, VI, and VII-VIII discrete; on Areas V and VI form distinct groups. Area V conical paragnaths absent. Area VI paragnaths present; paragnaths arranged in a roughly circular group; conical paragnaths present; smooth bars absent. Areas VII-VIII paragnaths present; conical paragnaths present; conical paragnaths arranged in one or more irregular lines forming a continuous band; conical paragnaths similar in size, or irregular mix of large and small paragnaths in a single band; rod-shaped paragnaths absent.

Dorsal notopodial ligule not markedly elongate on posterior chaetigers; not markedly broader on posterior chaetigers; not markedly reduced on posterior chaetigers. Prechaetal notopodial lobe present; smaller than dorsal notopodial ligule on anterior chaetigers, usually reduced or absent posteriorly. Dorsal cirrus not sub-terminally attached to dorsal notopodial ligule on posterior chaetigers; not terminally attached to dorsal notopodial ligule on posterior chaetigers; not terminally attached throughout all chaetigers.

Neuropodial postchaetal lobe present. Ventral neuropodial ligule of anterior chaetigers present. Ventral neuropodial ligule of anterior chaetigers approx. as long as acicular neuropodial ligule. Ventral neuropodial ligule on posterior chaetigers present. Ventral neuropodial ligule on posterior chaetigers similar to length of acicular neuropodial ligule.

Notoaciculae on chaetigers 1 and 2 absent. Notochaetae: homogomph spinigers present. Neurochaetae dorsal fascicle: heterogomph spinigers present; homogomph spinigers present; heterogomph falcigers in anterior chaetigers absent; on posterior chaetigers present; blades serrated; blades with teeth only slightly longer proximally than distally. Neurochaetae ventral fascicle: heterogomph spinigers present; homogomph spinigers present; heterogomph falcigers present; anterior chaetigers heterogomph falcigers with long blades absent; anterior chaetigers heterogomph falcigers with extra-long blades absent; anterior chaetigers heterogomph falcigers with short blades absent; posterior chaetigers heterogomph falcigers with long blades absent; posterior chaetigers heterogomph falcigers with extra-long blades present; posterior chaetigers heterogomph falcigers with short blades absent.

#### Remarks.

*Imajimainereis* contains a single species, *I.pacifica* de León-González & Solís-Weiss, 2000, which differs from all other Nereididae by having both papillae and paragnaths on the oral ring and neurochaetae including heterogomph spinigers.

*Imajimainereis* is recorded from the Gulf of California, eastern Pacific Ocean (de León-González and Solís-Weiss 2000).

### ﻿ Kainonereis

Taxon classificationAnimaliaPhyllodocidaNereididae

Chamberlin, 1919

AD44BE40-C6FE-5E06-A0D1-9D418EAF87C1

#### Type species.

*Kainonereisalata* Chamberlin, 1919.

#### WoRMS URL.

https://www.marinespecies.org/polychaeta/aphia.php?p=taxdetails&id=324852.

#### Sources.

Conde-Vela et al. (2018); Chamberlin (1919).

#### Diagnosis.

Dorsal cirrophores of chaetigers 5–7 of epitokes modified into flattened elytriform discs (minimal diagnosis). Notochaetae homogomph falcigers present; maxillary ring paragnaths absent; oral ring paragnaths absent; dorsal notopodial ligule not markedly reduced on posterior chaetigers (secondary diagnosis).

#### Description.

Palps anteriorly directed, or ventrally directed. Palpophore barrel-shaped, approximately equal width from base to palpostyle (not overly large compared with palpostyle). Prostomium the antennae are separate but sometimes basally fused in male epitokes. Tentacular cirri articulated.

Jaws with dentate cutting edge.

Maxillary ring of pharynx with papillae absent. Maxillary ring paragnaths absent. Oral ring paragnaths absent.

Dorsal notopodial ligule commences chaetiger 3, or chaetiger 4 (from chaetiger 4 in males, 3 in females); not markedly elongate on posterior chaetigers; not markedly reduced on posterior chaetigers. Prechaetal notopodial lobe present (subconical to digitate in atokes, rounded in epitokes). Dorsal cirrus not sub-terminally attached to dorsal notopodial ligule on posterior chaetigers.

Neuropodial prechaetal lobe absent. Neuropodial postchaetal lobe present; present throughout all chaetigers. Ventral neuropodial ligule of anterior chaetigers present. Ventral neuropodial ligule of anterior chaetigers approx. as long as acicular neuropodial ligule. Ventral neuropodial ligule on posterior chaetigers present.

Notoaciculae on chaetigers 1 and 2 absent. Notochaetae: homogomph spinigers present; homogomph falcigers present (in males, on anterior chaetigers); homogomph falcigers with multidentate blade with two or more small lateral teeth, first and subsequent lateral teeth much smaller than terminal tooth present. Neurochaetae dorsal fascicle: heterogomph spinigers absent; homogomph spinigers present; heterogomph falcigers in anterior chaetigers present. Neurochaetae ventral fascicle: heterogomph spinigers present; homogomph spinigers absent; heterogomph falcigers present; falcigers blade bowed, with convex margin.

Pygidium bilobate. Anal cirri form cirriform or conical.

#### Epitokes.

Dorsal cirrophores of chaetigers 5–7 of epitokes modified into flattened elytriform discs. Natatory epitokal modifications in males commence chaetiger 15. Natatory epitokal modifications in females commence chaetiger 15.

#### Remarks.

*Kainonereis* was originally described for an epitokous specimen in which dorsal cirri of chaetigers 5–7 were expanded into elytra-like structures. Unless epitokes are available *Kainonereis* is not separable from *Nicon* and *Websterinereis* (this does not imply that *Kainonereis* is invalid). A revision by Conde-Vela et al. (2018) redefined the genus and included atokous characters; five species are now recognised and epitokes can be identified using the key of Conde-Vela et al. (2018).

### ﻿ Kinberginereis

Taxon classificationAnimaliaPhyllodocidaNereididae

Pettibone, 1971

A548C2F2-63AF-5B86-936E-156FC2B5648D

#### Type species.

Nereis (Leptonereis) inermis Hoagland, 1920.

#### WoRMS URL.

https://www.marinespecies.org/polychaeta/aphia.php?p=taxdetails&id=843657.

#### Sources.

Pettibone (1971); Bakken et al. (2022).

#### Diagnosis.

Neurochaetae ventral fascicle heterogomph spinigers in anterior chaetigers with blades coarsely serrated proximally; oral ring papillae present; maxillary ring of pharynx with papillae absent (minimal diagnosis). Prostomium anterior margin indented; prechaetal notopodial lobe approximately equal to length of dorsal notopodial ligule at least on anterior chaetigers (thus notopodium of three similar sized ligules/lobes); antennae form cirriform (usually extending to or past palpophore); maxillary ring paragnaths absent (secondary diagnosis).

#### Description.

Palpophore barrel-shaped, approximately equal width from base to palpostyle (not overly large compared with palpostyle) (elongate). Prostomium anterior margin indented.

Jaws with dentate cutting edge, 20 teeth.

Maxillary ring of pharynx with papillae absent. Maxillary ring paragnaths absent. Oral ring papillae present. Oral ring papillae number two in total; arrangement solitary. Area V papillae absent; VI papillae present, one papilla (a single fleshy nob on each side); VII-VIII papillae absent. Oral ring paragnaths absent.

Dorsal notopodial ligule present; markedly reduced on posterior chaetigers. Prechaetal notopodial lobe present; approximately equal to length of dorsal notopodial ligule at least on anterior chaetigers (thus notopodium of three similar sized ligules/lobes); present on all chaetigers. Dorsal cirrus not sub-terminally attached to dorsal notopodial ligule on posterior chaetigers.

Neuropodial prechaetal lobe present. Neuropodial postchaetal lobe present; projecting beyond end of the acicular ligule; flattened. Ventral neuropodial ligule of anterior chaetigers present. Ventral neuropodial ligule of anterior chaetigers approx. as long as acicular neuropodial ligule. Ventral neuropodial ligule on posterior chaetigers present. Ventral neuropodial ligule on posterior chaetigers similar to length of acicular neuropodial ligule.

Notoaciculae on chaetigers 1 and 2 present. Notochaetae: homogomph spinigers present. Neurochaetae dorsal fascicle: heterogomph spinigers absent; homogomph spinigers present; heterogomph falcigers in anterior chaetigers absent; on posterior chaetigers absent. Neurochaetae ventral fascicle: heterogomph spinigers present; spinigers in anterior chaetigers with blades coarsely serrated proximally; homogomph spinigers absent; heterogomph falcigers absent.

#### Remarks.

The most recent taxonomic treatment of *Kinberginereis* is the original description of Pettibone (1971). *Kinberginereis* is most similar to *Kainonereis*, atokes differing only in that *Kinberginereis* has an indented prostomium but the prostomium of *Kainonereis* is entire. Epitokes of *Kainonereis* are distinctive in having elytriform expansion of dorsal cirrophores of chaetigers 5–7 but epitokes of *Kinberginereis* are unknown.

*Kinberginereis* includes a single species, *K.inermis* (Hoagland, 1920) described from a single specimen from shallow water in the Philippines; the only subsequent reports are unverified occurrence records now in the Smithsonian National Museum of Natural History from the Gulf of Mexico.

### ﻿ Laeonereis

Taxon classificationAnimaliaPhyllodocidaNereididae

Hartman, 1945

8C7A0D76-6047-5EB7-B63D-A6B04E21F426

#### Type species.

*Nereisculveri* Webster, 1879.

#### WoRMS URL.

https://www.marinespecies.org/polychaeta/aphia.php?p=taxdetails&id=181592.

#### Sources.

Pettibone (1971); Conde-Vela (2021).

#### Diagnosis.

Maxillary ring of pharynx with papillae in tufts (minimal diagnosis). Dorsal notopodial ligule commences chaetiger 1; neurochaetae ventral fascicle homogomph falcigers on posterior chaetigers present (secondary diagnosis).

#### Description.

Palpophore barrel-shaped, approximately equal width from base to palpostyle (not overly large compared with palpostyle). Prostomium anterior margin indented; longitudinal groove present.

Jaws with dentate cutting edge.

Maxillary ring of pharynx with papillae present; in tufts. Maxillary ring paragnaths absent. Oral ring papillae present. Oral ring papillae arrangement solitary. Area V papillae absent; VI papillae present; VII-VIII papillae present or absent (may be absent in juveniles). Oral ring paragnaths absent. Papillae triangular or conical on Area VI, rounded on Areas VII-VIII.

Dorsal notopodial ligule present; commences chaetiger 1; not markedly elongate on posterior chaetigers; not markedly broader on posterior chaetigers; not markedly reduced on posterior chaetigers. Prechaetal notopodial lobe present; smaller than dorsal notopodial ligule on anterior chaetigers, usually reduced or absent posteriorly; restricted to a limited number of anterior chaetigers. Notopodial acicular process absent. Dorsal cirrus not sub-terminally attached to dorsal notopodial ligule on posterior chaetigers; not terminally attached to dorsal notopodial ligule on posterior chaetigers; not terminally attached throughout all chaetigers.

Neuropodial prechaetal lobe absent. Neuropodial postchaetal lobe present; projecting beyond end of the acicular ligule; restricted to anterior chaetigers. Ventral neuropodial ligule of anterior chaetigers present. Ventral neuropodial ligule of anterior chaetigers approx. as long as acicular neuropodial ligule. Ventral neuropodial ligule on posterior chaetigers present. Ventral neuropodial ligule on posterior chaetigers similar to length of acicular neuropodial ligule.

Notoaciculae on chaetigers 1 and 2 absent. Notochaetae: homogomph spinigers present. Neurochaetae dorsal fascicle: heterogomph spinigers absent; homogomph spinigers present; heterogomph falcigers in anterior chaetigers absent; on posterior chaetigers absent; homogomph falcigers in anterior chaetigers present, or absent; on posterior chaetigers present, or absent. Neurochaetae ventral fascicle: heterogomph spinigers absent; homogomph spinigers present; heterogomph falcigers absent; homogomph falcigers on posterior chaetigers present (with long blades).

Pygidium funnel-shaped, crenulated, or multi-incised, with ventral incision. Anal cirri cirriform or conical.

#### Remarks.

The description and diagnosis of *Laeonereis* here is derived from the revision of Conde-Vela (2021) which is only slightly modified from that of Pettibone (1971) who recognised a single species of *Laeonereis*. Simultaneously with the publication of the morphological revision of *Laeonereis* by Conde-Vela (2021), Sampieri et al. (2021) published a molecular study of *Laeonereis* (but with no morphological component) and revealed seven or nine molecular OTUs. It is tantalising that Sampieri et al. (2021) and Conde-Vela (2021) discovered very similar species-level diversity from within the same geographic range but frustrating that neither apparently was aware of the others’ research.

Currently eight species of *Laeonereis* are recognised, largely confined to the Atlantic coasts of North and South America; they can be identified using the key of Conde-Vela (2021).

### ﻿ Leonnates

Taxon classificationAnimaliaPhyllodocidaNereididae

Kinberg, 1865

C3644EC1-EA46-55DC-A616-A86BEEAC1EA8

Nereis (Leonnates) auctt.
Laevispinereis He & Wu, 1989.

#### Type species.

*Leonnatesindicus* Kinberg, 1865.

#### WoRMS URL.

https://www.marinespecies.org/polychaeta/aphia.php?p=taxdetails&id=129374.

#### Sources.

Qiu and Qian (2000); Villalobos-Guerrero et al. (2022a).

#### Diagnosis.

Oral ring papillae present; maxillary ring paragnaths present; oral ring paragnaths absent; ventral neuropodial ligule on posterior chaetigers similar to length of acicular neuropodial ligule; neurochaetae dorsal fascicle homogomph spinigers present (minimal diagnosis; secondary diagnosis not attained).

#### Description.

Palpophore barrel-shaped, approximately equal width from base to palpostyle (not overly large compared with palpostyle), or massive subconical, flattened (palpostyle is minute by comparison). Palpophore surface with a single transverse groove (palpophores well developed). Prostomium anterior margin entire or indented (indented only in *L.persicus* and *L.stephensoni*); longitudinal groove present; anterior region sub-quadrangular or sub-rectangular. Tentacular belt greater than length of chaetiger 1.

Oesophageal caeca absent.

Jaws with smooth or slightly crenulate cutting edge or with dentate cutting edge.

Maxillary ring of pharynx with papillae present, or absent; solitary. Maxillary ring paragnaths present. Area I conical paragnaths present, or absent; II conical paragnaths present; III conical paragnaths present, or absent; III rod-like paragnaths absent; IV paragnaths present; IV conical paragnaths present; IV smooth bar-like paragnaths present, or absent; IV rod-like paragnaths absent. Oral ring papillae present. Oral ring papillae arrangement solitary. Area V papillae present, or absent; VI papillae present; VII-VIII papillae present. Oral ring paragnaths absent.

Dorsal notopodial ligule not markedly elongate on posterior chaetigers; not markedly broader on posterior chaetigers; not markedly reduced on posterior chaetigers. Prechaetal notopodial lobe present; smaller than dorsal notopodial ligule on anterior chaetigers, usually reduced or absent posteriorly, or approximately equal to length of dorsal notopodial ligule at least on anterior chaetigers (thus notopodium of three similarly sized ligules/lobes); present on all chaetigers, or restricted to a limited number of anterior chaetigers. Notopodial acicular process absent. Dorsal cirrus not sub-terminally attached to dorsal notopodial ligule on posterior chaetigers; not terminally attached to dorsal notopodial ligule on posterior chaetigers; not terminally attached throughout all chaetigers.

Neuropodial prechaetal lobe absent. Neuropodial postchaetal lobe present; projecting beyond end of the acicular ligule; present throughout all chaetigers or restricted to anterior chaetigers; digitiform. Ventral neuropodial ligule of anterior chaetigers present. Ventral neuropodial ligule of anterior chaetigers approx. as long as acicular neuropodial ligule. Ventral neuropodial ligule on posterior chaetigers present. Ventral neuropodial ligule on posterior chaetigers similar to length of acicular neuropodial ligule.

Notoaciculae on chaetigers 1 and 2 absent. Notochaetae: homogomph spinigers present; homogomph falcigers present, or absent. Neurochaetae dorsal fascicle: heterogomph spinigers present, or absent (present in *L.fujianensis*); homogomph spinigers present; sesquigomph spinigers present, or absent (present in *L.fujianensis*); sesquigomph falcigers present, or absent; heterogomph falcigers in anterior chaetigers present, or absent; on posterior chaetigers present, or absent; homogomph falcigers in anterior chaetigers present, or absent; on posterior chaetigers present, or absent. Neurochaetae ventral fascicle: sesquigomph falcigers present, or absent; blade with a single distal tooth; heterogomph spinigers present, or absent; homogomph spinigers present, or absent; sesquigomph spinigers present, or absent; heterogomph falcigers present, or absent; falcigers blade bowed, with convex margin; heterogomph falcigers blade lacking distinct tendon on terminal tooth; homogomph falcigers in anterior chaetigers present, or absent; on posterior chaetigers present, or absent. Ventral fascicle neuropodial falcigers apparently vary considerably between species.

Anal cirri form cirriform or conical.

#### Remarks.

The current description and diagnosis follow Qiu and Qian (2000) and emendments by Villalobos-Guerrero et al. (2022a). Original descriptions sometimes report the presence of sesquigomph falcigers and spinigers but as noted by Qiu and Qian (2000) some of these interpretations are inconsistent, with the same chaetal forms being given different names by some authors. However Qiu and Qian (2000) also do not interpret these terms consistently: they do not use the term sesquigomph yet their figures 3B and 3E (*Leonnatesindicus* Kinberg, 1865), 5E (*L.nierstraszi* Horst, 1924), 7B (*L.decipiens* Fauvel, 1929), 9D (*L.persicus* Wesenberg-Lund, 1949, and 14D (*L.crinitus* Hutchings & Reid, 1991, albeit damaged) all show sesquigomph articulation as accepted by other authors, e.g. Villalobos-Guerrero et al. (2022a: fig. 12i, j; *Parasetiairritabilis* (Webster, 1879)), Bakken et al. (2022: figs 7.13.3.3.3: C, 7.13.3.3.4: C), de León-González and Salazar-Vallejo (2003: fig. 1E, F) (*Leonnatescrosnieri* de León-González & Salazar-Vallejo, 2003).

*Leonnates* includes 13 species with the greatest diversity in the tropical Indo-Pacific. Qiu and Qian (2000) provide a key to the seven species known at that time.

### ﻿ Leptonereis

Taxon classificationAnimaliaPhyllodocidaNereididae

Kinberg, 1865

BD8300F9-84E4-5EEF-A4DA-55C68B821E5B

Nereis (Leptonereis) auctt.

#### Type species.

*Leptonereislaevis* Kinberg, 1865.

#### WoRMS URL.

https://www.marinespecies.org/polychaeta/aphia.php?p=taxdetails&id=152401.

#### Sources.

Hartman 1945; Pettibone 1971.

#### Diagnosis.

Dorsal cirrus terminally attached to dorsal notopodial ligule on posterior chaetigers; maxillary ring paragnaths absent (minimal diagnosis). Dorsal notopodial ligule markedly broader on posterior chaetigers; prostomium anterior margin entire; maxillary ring of pharynx with P-bar paragnaths absent (secondary diagnosis).

#### Description.

Palpophore barrel-shaped, approximately equal width from base to palpostyle (not overly large compared with palpostyle).

Maxillary ring of pharynx with papillae absent. Maxillary ring paragnaths absent. Oral ring papillae absent. Oral ring paragnaths absent.

Dorsal notopodial ligule present; markedly elongate on posterior chaetigers; markedly broader on posterior chaetigers; not markedly reduced on posterior chaetigers. Dorsal cirrus not sub-terminally attached to dorsal notopodial ligule on posterior chaetigers; terminally attached to dorsal notopodial ligule on posterior chaetigers; not terminally attached throughout all chaetigers.

Neuropodial postchaetal lobe absent. Ventral neuropodial ligule of anterior chaetigers present. Ventral neuropodial ligule of anterior chaetigers approx. as long as acicular neuropodial ligule. Ventral neuropodial ligule on posterior chaetigers present. Ventral neuropodial ligule on posterior chaetigers similar to length of acicular neuropodial ligule.

Notochaetae: homogomph spinigers present. Neurochaetae homogomph and heterogomph spinigers and heterogomph falcigers, but their distribution in dorsal and ventral fascicles is unknown.

#### Remarks.

The description of *Leptonereis* given here follows the most recent treatment (Pettibone 1971) which in turn was based on new descriptions and figures of the type by Kinberg (1910) and Hartman (1945). However, many characters including articulation of the chaetae remain unverified.

*Leptonereis* includes a single species, *L.laevis* Kinberg, 1865, based on a single specimen from Guayaquil, Ecuador and now recorded from tropical east Pacific coasts of North and South America.

### ﻿ Lycastonereis

Taxon classificationAnimaliaPhyllodocidaNereididae

Rao, 1981

48DE0D96-E343-570A-9B2A-FA32B74972CD

#### Type species.

*Lycastonereisindica* Rao, 1981.

#### WoRMS URL.

https://www.marinespecies.org/polychaeta/aphia.php?p=taxdetails&id=324857.

#### Sources.

Rao (1981); Conde-Vela (2019a).

#### Diagnosis.

Tentacular cirri three pairs; palpophore surface with a single transverse groove (palpophores well developed) (minimal diagnosis). Neurochaetae dorsal fascicle homogomph falcigers on posterior chaetigers present; maxillary ring paragnaths absent; prostomium anterior margin entire (secondary diagnosis).

#### Description.

Palpophore barrel-shaped, approximately equal width from base to palpostyle (not overly large compared with palpostyle). Palpophore surface with a single transverse groove (palpophores well developed). Prostomium anterior margin entire. Tentacular belt greater than length of chaetiger 1. Tentacular cirri three pairs.

Jaws with dentate cutting edge.

Maxillary ring of pharynx with papillae present. Maxillary ring paragnaths absent. Oral ring papillae present. Oral ring papillae arrangement solitary. Area V papillae absent; VI papillae present; VII-VIII papillae present, arranged in a single row. Oral ring paragnaths absent.

Dorsal notopodial ligule markedly reduced or absent on posterior chaetigers. Prechaetal notopodial lobe present; restricted to a limited number of anterior chaetigers.

Neuropodial prechaetal lobe absent. Neuropodial postchaetal lobe present; not projecting beyond end of the acicular ligule; digitiform. Ventral neuropodial ligule of anterior chaetigers present. Ventral neuropodial ligule of anterior chaetigers approx. as long as acicular neuropodial ligule. Ventral neuropodial ligule on posterior chaetigers present. Ventral neuropodial ligule on posterior chaetigers short, up to half length of acicular neuropodial ligule.

Notoaciculae on chaetigers 1 and 2 absent. Notochaetae: homogomph spinigers present. Neurochaetae dorsal fascicle: heterogomph spinigers absent; homogomph spinigers present; dorsal fascicle heterogomph falcigers in anterior chaetigers absent; on posterior chaetigers absent; dorsal fascicle homogomph falcigers in anterior chaetigers present; on posterior chaetigers present; ventral fascicle heterogomph spinigers absent; homogomph spinigers present; sesquigomph spinigers present. Neurochaetae ventral fascicle: heterogomph falcigers absent; homogomph falcigers in anterior chaetigers present; on posterior chaetigers present.

#### Remarks.

This easily diagnosed genus from estuaries in India includes a single species. Conde-Vela (2019a) provides a redescription based on non-type material which is topotypic and shares the unusual morphological characters of the original description including the presence of only three pairs of tentacular cirri. Conde-Vela (2019a) also clarified the surname of the author, which is Rao, not ‘Nageswara-Rao’ or ‘Nageswara Rao’ as stated in much of the literature. Although the double surname is a more precise authority name, we follow Conde-Vela (2019a) because Rao appears to be an uncommon name among polychaete taxon authors and thus not easily confused.

### ﻿ Micronereides

Taxon classificationAnimaliaPhyllodocidaNereididae

Day, 1963

0FD2B90F-6BE2-5215-9ADB-53D8CB09F980

#### Type species.

*Micronereidescapensis* Day, 1963.

#### WoRMS URL.

https://www.marinespecies.org/polychaeta/aphia.php?p=taxdetails&id=324861.

#### Sources.

Day (1963).

#### Diagnosis.

Tentacular belt represented by two distinct segments each carrying a pair of tentacular cirri (minimal diagnosis). Dorsal notopodial ligule absent; neurochaetae ventral fascicle sesquigomph spinigers present (secondary diagnosis).

#### Description.

Palpophore barrel-shaped, approximately equal width from base to palpostyle (not overly large compared with palpostyle). Eyes absent. Prostomium with eyes, if not absent, indistinct and likely to be missed. Tentacular belt equal to or less than length of chaetiger 1; represented by two distinct segments each carrying a pair of tentacular cirri.

Jaws with dentate cutting edge, seven teeth.

Maxillary ring of pharynx with papillae absent. Maxillary ring paragnaths absent. Oral ring papillae absent. Oral ring paragnaths absent.

Dorsal notopodial ligule absent. Prechaetal notopodial lobe absent. Notopodial acicular process absent.

Neuropodial prechaetal lobe absent. Neuropodial postchaetal lobe absent. Ventral neuropodial ligule of anterior chaetigers present. Ventral neuropodial ligule of anterior chaetigers short, up to half length of acicular neuropodial ligule. Ventral neuropodial ligule on posterior chaetigers present. Ventral neuropodial ligule on posterior chaetigers similar to length of acicular neuropodial ligule. Dorsal notopodial ligule elongate, exceeding length of dorsal cirrus.

Notochaetae: homogomph spinigers present. Neurochaetae dorsal fascicle: heterogomph spinigers absent; homogomph spinigers present; heterogomph falcigers in anterior chaetigers absent; on posterior chaetigers absent; ventral fascicle heterogomph spinigers absent; homogomph spinigers present; sesquigomph spinigers present (possibly, chaetae need re-examination).

#### Remarks.

*Micronereides* was erected to contain *M.capensis* Day, 1963 a small nereidid lacking pharyngeal papillae or paragnaths and in which tentacular cirri arise from two distinct anterior segments (unique among Nereididae). A revised diagnosis was provided by Banse (1977) including the observation that accessory ventral cirri are present on anterior segments thus placing the genus in Gymnonereidinae.

The genus is still only known from a single species, *M.capensis*, recorded from shelf depths in the South Atlantic Ocean.

### ﻿ Micronereis

Taxon classificationAnimaliaPhyllodocidaNereididae

Claparède, 1863

B54F0B95-D187-5F51-B03F-00845FCB5AD9

Notophycus Knox & Cameron, 1970.
Phyllodocella Fauchald & Belman, 1972.
Quadricirra Banse, 1977.

#### Type species.

*Micronereisvariegata* Claparède, 1863.

#### WoRMS URL.

https://www.marinespecies.org/polychaeta/aphia.php?p=taxdetails&id=129375.

#### Sources.

Paxton (1983).

#### Diagnosis.

Palpostyles absent (palps undivided, minute) (minimal diagnosis). Maxillary ring of pharynx undivided (secondary diagnosis).

#### Description.

Antennae absent. Palps ventrally directed. Palpophore surface with a single transverse groove (palpophores well developed); palpostyles absent (palps undivided, minute).

Jaws with dentate cutting edge.

Maxillary ring of pharynx undivided.

Maxillary ring of pharynx with papillae absent; undivided maxillary ring with two paragnaths in total. Oral ring papillae absent. Oral ring paragnaths present; on Areas V and VI not recognisably distinct. Crown-shaped oral ring paragnaths present. Area V conical paragnaths absent.

Dorsal notopodial ligule absent. Prechaetal notopodial lobe absent. Notopodial acicular process absent. Acicular notopodial ligule absent.

Neuropodial postchaetal lobe absent. Ventral neuropodial ligule of anterior chaetigers absent. Ventral neuropodial ligule on posterior chaetigers absent.

Notoaciculae on chaetigers 1 and 2 absent (only confirmed as yet for *M.bansei*). Notochaetae: homogomph spinigers present; homogomph falcigers present or absent; homogomph falcigers with multidentate blade with two or more small lateral teeth, first and subsequent lateral teeth much smaller than terminal tooth present. Neurochaetae dorsal fascicle: heterogomph spinigers absent; homogomph spinigers present; heterogomph falcigers in anterior chaetigers absent; homogomph falcigers in anterior chaetigers present; on posterior chaetigers present. Neurochaetae ventral fascicle: heterogomph spinigers absent; homogomph spinigers present; heterogomph falcigers absent.

Anal cirri form cirriform or conical.

#### Remarks.

*Micronereis* is a genus of small nereidids generally associated with algal turfs in intertidal and shallow (to ~ 30 m) marine waters. *Micronereis* species differ from other Nereididae in lacking antennae and having a pharynx that is not fully eversible. They are sexually dimorphic and males have distinctive neurochaetae that function as copulatory hooks not found in other Nereididae.

*Micronereis* currently includes ten accepted species which collectively have a wide global distribution. Paxton (1983) revised the genus and included a key to males but three more species have since been recognised.

### ﻿ Namalycastis

Taxon classificationAnimaliaPhyllodocidaNereididae

Hartman, 1959

C39C0DA3-0F57-5783-89CE-D206253F0F3C

#### Type species.

*Lycastisabiuma* Grube, 1872.

#### WoRMS URL.

https://www.marinespecies.org/polychaeta/aphia.php?p=taxdetails&id=129376.

#### Sources.

Glasby (1999).

#### Diagnosis.

Palpophore surface without grooves or striae (palps short, compact); antennae form subconical (shorter than palpophore) (minimal diagnosis). Prostomium anterior margin indented (secondary diagnosis).

#### Description.

Palpophore barrel-shaped, approximately equal width from base to palpostyle (not overly large compared with palpostyle). Palpophore surface without grooves or striae (palps short, compact) or with a single transverse groove (palpophores well developed); palpostyles spherical. Prostomium anterior margin indented; longitudinal groove present. Tentacular belt equal to or less than length of chaetiger 1.

Jaws with dentate cutting edge.

Maxillary ring of pharynx with papillae absent. Maxillary ring paragnaths absent. Oral ring papillae absent. Oral ring paragnaths absent.

Notopodium strongly reduced, without distinct lobes or ligules. Dorsal cirrus arising from basal cirrophore (weakly developed; only on anterior chaetigers).

Neuropodial postchaetal lobe absent. Ventral neuropodial ligule of anterior chaetigers absent. Ventral neuropodial ligule on posterior chaetigers absent.

Notoaciculae on chaetigers 1 and 2 present. Notochaetae: homogomph spinigers absent; sesquigomph spinigers present, or absent. Neurochaetae dorsal fascicle: heterogomph spinigers present (rarely), or absent; homogomph spinigers absent; sesquigomph spinigers present; heterogomph falcigers in anterior chaetigers present, or absent (rarely); on posterior chaetigers present, or absent; blades smooth, or serrated; blades with teeth only slightly longer proximally than distally. Neurochaetae ventral fascicle: heterogomph spinigers present; spinigers in anterior chaetigers with blades evenly serrated throughout, or coarsely serrated proximally (rarely); on posterior chaetigers with blades finely serrated proximally, or coarsely serrated proximally; homogomph spinigers absent; heterogomph falcigers present, or absent (rarely); anterior chaetigers heterogomph falcigers with long blades absent; anterior chaetigers heterogomph falcigers with extra-long blades present; anterior chaetigers heterogomph falcigers with short blades absent; posterior chaetigers heterogomph falcigers with long blades absent; posterior chaetigers heterogomph falcigers with extra-long blades present; posterior chaetigers heterogomph falcigers with short blades absent.

Pygidium with three incisions marking lateral and dorsal lobes. Anal cirri form cirriform or conical, or flattened, resembling posterior dorsal cirri.

#### Reproductive characters.

Oocyte spherical.

#### Remarks.

*Namalycastis* currently includes 33 accepted species recorded mainly from intertidal and supralittoral areas, including freshwater, of the tropics and subtropics. Together with sister-group *Namanereis*, they are one of only a few polychaetes to be found in association with riparian vegetation. Because they have an unadorned pharynx and a simplified parapodia, distinguishing species relies heavily on differences in chaetae, form of sensory organs of the head, and pigmentation patterns in living specimens. The modern concept of the subfamily and genus was introduced by Hartman (1959) and later reviewed by Glasby (1999), who included a key to all known species at the time. Since Glasby (1999) there have been five species described: *Namalycastiscaetensis* Alves & Santos, 2016, *Namalycastisglasbyi* Fernando & Rajasekaran, 2007, *Namalycastisjaya* Magesh, Kvist & Glasby, 2012, *Namalycastisocculta* Conde-Vela, 2013 and *Namalycastisrhodochorde* Glasby, Miura, Nishi & Junardi, 2007; however, *Namalycastisocculta* Conde-Vela, 2013 is now accepted as *Namanereisocculta* (Conde-Vela, 2013). Magesh et al. (2013) provided a key to Indian species and Conde-Vela (2013) provided a key to Caribbean species.

### ﻿ Namanereis

Taxon classificationAnimaliaPhyllodocidaNereididae

Chamberlin, 1919

E4E1CB01-7AE5-5147-9A46-E3FB005E109E

Cryptonereis Gibbs, 1971.
Lycastella Feuerborn, 1932.
Lycastilla Solís-Weiss & Espinasa, 1991.
Lycastoides Jakubova, 1930.
Lycastopsis Augener, 1922.

#### Type species.

*Lycastisquadraticeps* Blanchard in Gay, 1849.

#### WoRMS URL.

https://www.marinespecies.org/polychaeta/aphia.php?p=taxdetails&id=129377.

#### Sources.

Glasby (1999).

#### Diagnosis.

Palpophore surface without grooves or striae (palps short, compact); prostomium anterior margin entire (minimal diagnosis). Palpostyles spherical; dorsal cirrus simple, lacking basal cirrophore (secondary diagnosis).

#### Description.

Antennae present, or absent (rarely). Palpophore barrel-shaped, approximately equal width from base to palpostyle (not overly large compared with palpostyle). Palpophore surface without grooves or striae (most species; palps short, compact) or with a single transverse groove (palpophores well developed); palpostyles spherical. Eyes present, or absent. Tentacular belt equal to or less than length of chaetiger 1. Tentacular cirri four pairs, or three pairs.

Jaws forms with a crenulate cutting edge have 2 teeth proximally, with smooth or slightly crenulate cutting edge or with dentate cutting edge.

Maxillary ring of pharynx with papillae absent. Maxillary ring paragnaths absent. Oral ring papillae absent. Oral ring paragnaths absent.

Notopodium strongly reduced, without distinct lobes or ligules.

Neuropodial postchaetal lobe absent. Ventral neuropodial ligule of anterior chaetigers absent. Ventral neuropodial ligule on posterior chaetigers absent.

Notoaciculae on chaetigers 1 and 2 present. Notochaetae: homogomph spinigers absent; sesquigomph spinigers present (rarely), or absent. Neurochaetae dorsal fascicle: heterogomph spinigers present (rarely), or absent; homogomph spinigers absent; sesquigomph spinigers present, or absent; heterogomph falcigers in anterior chaetigers present; on posterior chaetigers present; blades serrated; blades with teeth only slightly longer proximally than distally, or much longer proximally than distally. Neurochaetae ventral fascicle: heterogomph spinigers present (rarely), or absent; spinigers in anterior chaetigers with blades evenly serrated throughout; on posterior chaetigers with blades finely serrated proximally; homogomph spinigers absent; heterogomph falcigers present (some forms with very long blades = pseudospinigers); anterior chaetigers heterogomph falcigers with long blades present; anterior chaetigers heterogomph falcigers with extra-long blades present; anterior chaetigers heterogomph falcigers with short blades absent; posterior chaetigers heterogomph falcigers with long blades present, or absent; posterior chaetigers heterogomph falcigers with extra-long blades present, or absent; posterior chaetigers heterogomph falcigers with short blades absent.

Pygidium with three incisions marking lateral and dorsal lobes. Anal cirri form cirriform or conical, or short, stout and appearing as an extension of the pygidium.

#### Reproductive characters.

Oocyte spherical (rarely), or ovoid.

#### Remarks.

*Namanereis* currently includes 27 accepted species recorded mainly from intertidal, supralittoral and terrestrial areas, including the freshwaters of streams, caves, and underground aquifers. Although mainly found in the tropics and subtropics one species, *Namanereisquadraticeps* (Blanchard in Gay 1849), has a circum-subantarctic/temperate distribution. Together with sister-group *Namalycastis*, they are one of only a few polychaetes to be found in association with riparian vegetation. Because they have an unadorned pharynx and a simplified parapodia, distinguishing species relies heavily on differences in chaetae and form of sensory organs of the head. The modern concept of the subfamily and genus was introduced by Hartman (1959) and reviewed by Glasby (1999), the latter who included a key to all known species at the time. Since Glasby (1999) there have been seven species described: *Namanereiscanariarum* Núñez, Glasby & Naranjo, 2020, *Namanereischristopheri* Conde-Vela, 2017, *Namanereisgesae* Fiege & Van Damme, 2002, *Namanereisllanetensis* Núñez, Glasby & Naranjo, 2020, *Namanereisocculta* (Conde-Vela, 2013), *Namanereispilbarensis* Glasby, Fiege & Van Damme, 2014, and *Namanereissocotrensis* Glasby, Fiege & Van Damme, 2014, making this genus one of the most studied in the last 20 or so years. As noted by Alves et al. (2018) in a morphological phylogenetic study of the subfamily, *Lycastoidesalticola* Johnson, 1903 is also part of the *Namanereis* clade, but the species cannot take the name *Namanereis*, as *Lycastoides* is the senior genus (Read and Fauchald 2023). Conde-Vela (2017) provides an updated key to *Namanereis* species of the World.

### ﻿ Neanthes

Taxon classificationAnimaliaPhyllodocidaNereididae

Kinberg, 1865

059DAF52-0A47-5FDC-B789-80748E36170E

Nereis (Neanthes) auctt.
Nereis (Neanthioides) Rioja, 1918.
Praxithea Malmgren, 1867.

#### Type species.

*Neanthesvaalii* Kinberg, 1865.

#### WoRMS URL.

https://www.marinespecies.org/polychaeta/aphia.php?p=taxdetails&id=129378.

#### Sources.

Bakken et al. (2022).

#### Diagnosis.

Maxillary ring paragnaths present; neurochaetae dorsal fascicle heterogomph falcigers in anterior chaetigers present; dorsal notopodial ligule not markedly broader on posterior chaetigers; oral ring papillae absent; notochaetae homogomph falcigers absent; notochaetae sesquigomph falcigers absent; neurochaetae dorsal fascicle simple chaetae (fused falcigers) absent; Area VI smooth bars absent; notoaciculae on chaetigers 1 and 2 absent (minimal diagnosis; secondary diagnosis not attained).

#### Description.

Palpophore barrel-shaped, approximately equal width from base to palpostyle (not overly large compared with palpostyle), or massive subconical, flattened (palpostyle is minute by comparison) (rarely). Palpophore surface without grooves or striae or with a single transverse groove (palpophores well developed) or with several oblique grooves or striae (palpophores well developed). Eyes present, or absent. Tentacular belt greater than length of chaetiger 1.

Oesophageal caeca present, or absent.

Jaws with dentate cutting edge.

Maxillary ring of pharynx with papillae absent. Maxillary ring paragnaths present. Area I conical paragnaths present; II conical paragnaths present, or absent; III conical paragnaths present, or absent; III rod-like paragnaths absent; IV paragnaths present, or absent; IV conical paragnaths present, or absent; IV smooth bar-like paragnaths present, or absent; IV rod-like paragnaths absent. Oral ring papillae absent. Oral ring paragnaths present, or absent; with Areas V, VI, and VII-VIII discrete, or comprising a continuous ring dorsally and ventrally, discrete groups not recognisable; on Areas V and VI form distinct groups, or not recognisably distinct. Area V conical paragnaths present, or absent. Area VI paragnaths present, or absent; paragnaths arranged in a roughly circular group, or in lines or arcs; conical paragnaths present; smooth bars absent. Areas VII-VIII paragnaths present, or absent; conical paragnaths present; conical paragnaths arranged in one or more irregular lines forming a continuous band; conical paragnaths similar in size, or irregular mix of large and small paragnaths in a single band, or differentiated, with a separate band of minute paragnaths also present; rod-shaped paragnaths absent.

Dorsal notopodial ligule markedly elongate on anterior chaetigers, or not markedly elongate on anterior chaetigers; markedly elongate on posterior chaetigers, or not markedly elongate on posterior chaetigers; not markedly broader on posterior chaetigers; markedly reduced on posterior chaetigers, or not markedly reduced on posterior chaetigers. Prechaetal notopodial lobe present, or absent; smaller than dorsal notopodial ligule on anterior chaetigers, usually reduced or absent posteriorly, or approximately equal to length of dorsal notopodial ligule at least on anterior chaetigers (thus notopodium of three similar sized ligules/lobes); present on all chaetigers, or restricted to a limited number of anterior chaetigers. Notopodial acicular process present, or absent; reducing in size posteriorly, last present on chaetiger 5–25. Dorsal cirrus sub-terminally attached to dorsal margin of dorsal notopodial ligule on posterior chaetigers, or not sub-terminally attached to dorsal notopodial ligule on posterior chaetigers; not terminally attached to dorsal notopodial ligule on posterior chaetigers; not terminally attached throughout all chaetigers.

Neuropodial prechaetal lobe absent. Neuropodial postchaetal lobe absent, or present; projecting beyond end of the acicular ligule, or not projecting beyond end of the acicular ligule; present throughout all chaetigers, or restricted to anterior chaetigers; digitiform. Ventral neuropodial ligule of anterior chaetigers present. Ventral neuropodial ligule of anterior chaetigers approx. as long as acicular neuropodial ligule, or short, up to half length of acicular neuropodial ligule. Ventral neuropodial ligule on posterior chaetigers present, or absent. Ventral neuropodial ligule on posterior chaetigers similar to length of acicular neuropodial ligule, or short, up to half length of acicular neuropodial ligule.

Notoaciculae on chaetigers 1 and 2 absent. Notochaetae: homogomph spinigers present. Neurochaetae dorsal fascicle: heterogomph spinigers present, or absent; homogomph spinigers present; heterogomph falcigers in anterior chaetigers present; on posterior chaetigers present, or absent; blades serrated. Neurochaetae ventral fascicle: heterogomph spinigers present, or absent; homogomph spinigers present, or absent; heterogomph falcigers present; anterior chaetigers heterogomph falcigers with long blades present, or absent; anterior chaetigers heterogomph falcigers with extra-long blades present, or absent; anterior chaetigers heterogomph falcigers with short blades present, or absent; posterior chaetigers heterogomph falcigers with long blades present, or absent; posterior chaetigers heterogomph falcigers with extra-long blades present, or absent; posterior chaetigers heterogomph falcigers with short blades present, or absent; heterogomph falcigers blade lacking distinct tendon on terminal tooth.

Anal cirri form cirriform or conical.

#### Remarks.

*Neanthes*, even after removing some species to *Alitta* and *Pseudonereis*, was found by Bakken and Wilson (2005) to contain morphologically dissimilar species. Our diagnosis here compounds the problem since the description is expanded to include species with variable palpophore morphology (*N.gisserana* (Horst, 1924) and *N.glandicincta* (Southern, 1921)) and with elongate dorsal notopodial lobe on all chaetigers (*N.articulata* Knox, 1960, *N.crucifera* (Grube, 1878), and *N.mossambica* Day, 1957) or only on posterior chaetigers (*N.mancorae* Berkeley & Berkeley, 1961 and *N.noodti* Hartmann-Schröder, 1962). Furthermore, *Neanthes* includes a subset of species having well-developed prechaetal notopodial lobes, giving the notopodia a tri-lobed appearance, which differs from the majority of bilobed species (Bakken 2006). However, the genus must still comprise several unrelated groups. *Neanthes* currently includes 88 species. There are no comprehensive keys or identification guides but there are several tabular comparisons of subsets of species, for example Asian species (Hsueh 2019a; Villalobos-Guerrero and Idris 2021) and deep-sea species (Shimabukuro et al. 2017).

### ﻿ Nectoneanthes

Taxon classificationAnimaliaPhyllodocidaNereididae

Imajima, 1972

4F412C26-EBAB-5BBF-B46E-A33CC671C29B

#### Type species.

Nereis (Alitta) oxypoda Marenzeller, 1879.

#### WoRMS URL.

https://www.marinespecies.org/polychaeta/aphia.php?p=taxdetails&id=324862.

#### Sources.

Sato (2013).

#### Diagnosis.

Dorsal notopodial ligule markedly broader on posterior chaetigers; notochaetae sesquigomph spinigers present (minimal diagnosis). Palpophore massive subconical, flattened (palpostyle is minute by comparison); neurochaetae dorsal fascicle heterogomph spinigers present (secondary diagnosis).

#### Description.

Palpophore massive subconical, flattened (palpostyle is minute by comparison).

Jaws with dentate cutting edge.

Maxillary ring of pharynx with papillae absent. Area I conical paragnaths present; II conical paragnaths present; III conical paragnaths present; III rod-like paragnaths absent; IV paragnaths present; IV conical paragnaths present; IV rod-like paragnaths absent. Oral ring paragnaths present; with Areas V, VI and VII-VIII discrete; on Areas V and VI form distinct groups. Area V conical paragnaths present. Area VI paragnaths present; paragnaths arranged in a roughly circular group; conical paragnaths present; smooth bars absent. Areas VII-VIII paragnaths present; conical paragnaths present; conical paragnaths arranged in one or more irregular lines forming a continuous band; conical paragnaths similar in size, or irregular mix of large and small paragnaths in a single band; rod-shaped paragnaths absent.

Dorsal notopodial ligule markedly elongate on posterior chaetigers; markedly broader on posterior chaetigers. Prechaetal notopodial lobe present; approximately equal to length of dorsal notopodial ligule at least on anterior chaetigers (thus notopodium of three similar sized ligules/lobes); present on all chaetigers. Dorsal cirrus sub-terminally attached to dorsal margin of dorsal notopodial ligule on posterior chaetigers; not terminally attached to dorsal notopodial ligule on anterior or posterior chaetigers.

Neuropodial prechaetal lobe absent. Neuropodial postchaetal lobe present; projecting beyond end of the acicular ligule; present throughout all chaetigers; digitiform. Ventral neuropodial ligule of anterior chaetigers present. Ventral neuropodial ligule of anterior chaetigers approx. as long as acicular neuropodial ligule. Ventral neuropodial ligule on posterior chaetigers present. Ventral neuropodial ligule on posterior chaetigers similar to length of acicular neuropodial ligule. Notopodial dorsal ligule with prominent ovoid lobe medial to the dorsal cirrus in middle and posterior parapodia.

Notoaciculae on chaetigers 1 and 2 present. Notochaetae: homogomph spinigers present; sesquigomph spinigers present. Neurochaetae dorsal fascicle: heterogomph spinigers present; homogomph spinigers present; sesquigomph spinigers present. Neurochaetae ventral fascicle: heterogomph spinigers present; homogomph spinigers present; heterogomph falcigers present (in small specimens); falcigers blade tapering, with straight margin; anterior chaetigers heterogomph falcigers with long blades absent; anterior chaetigers heterogomph falcigers with extra-long blades present; anterior chaetigers heterogomph falcigers with short blades absent; posterior chaetigers heterogomph falcigers with long blades absent; posterior chaetigers heterogomph falcigers with extra-long blades present; posterior chaetigers heterogomph falcigers with short blades absent.

Anal cirri form cirriform or conical.

#### Remarks.

*Nectoneanthes* was treated as a synonym of *Neanthes* by Wilson (1988) and Bakken and Wilson (2005). Sato (2013) showed Wilson (1988) to be incorrect in treating *Nectoneanthesoxypoda* (Marenzeller, 1879) as an epitokal form and resurrected *Nectoneanthes* and described a second species for the genus. The description by Sato (2013) is followed here.

Sato (2013) provided a key to the two species of *Nectoneanthes*; both species occur on the north-west Pacific coast, with *N.oxypoda* also recorded by Sato (2013) from southern Australia and the Persian Gulf.

### ﻿ Nereis

Taxon classificationAnimaliaPhyllodocidaNereididae

Linnaeus, 1758

06161371-38D6-527E-A638-D12FF23784E8

Heteronereis Örsted, 1843.
Johnstonia Quatrefages, 1850.
Lycoris Lamarck, 1818.
Naumachius Kinberg, 1865.
Nereis (Nereis) auctt.
Thoosa Kinberg, 1865.

#### Type species.

*Nereispelagica* Linnaeus, 1758.

#### WoRMS URL.

https://www.marinespecies.org/polychaeta/aphia.php?p=taxdetails&id=129379.

#### Sources.

Bakken et al. (2022).

#### Diagnosis.

Notochaetae homogomph falcigers present; maxillary ring paragnaths present; Area II rod-like paragnaths absent; dorsal notopodial ligule not markedly elongate on posterior chaetigers; antennae present; oral ring papillae absent (minimal diagnosis; secondary diagnosis not attained).

#### Description.

Palpophore barrel-shaped, approximately equal width from base to palpostyle (not overly large compared with palpostyle). Palpophore surface with a single transverse groove (palpophores well developed). Eyes present, or absent. Tentacular belt greater than length of chaetiger 1.

Oesophageal caeca present, or absent.

Jaws with smooth or slightly crenulate cutting edge or with dentate cutting edge.

Maxillary ring of pharynx with papillae absent. Maxillary ring paragnaths present. Area I conical paragnaths present, or absent; II conical paragnaths present, or absent; III conical paragnaths present, or absent; III rod-like paragnaths absent; IV paragnaths present; IV conical paragnaths present; IV smooth bar-like paragnaths present, or absent; IV rod-like paragnaths absent. Oral ring papillae absent. Oral ring paragnaths present, or absent; Areas V, VI, and VII-VIII discrete, forming distinct groups. Area V conical paragnaths present, or absent; arranged in a triangle, or in a longitudinal line. Area VI paragnaths present, or absent; paragnaths arranged in a roughly circular group; conical paragnaths present; smooth bars absent. Areas VII-VIII paragnaths present, or absent; conical paragnaths present; conical paragnaths arranged in one or more irregular lines forming a continuous band; conical paragnaths similar in size, or irregular mix of large and small paragnaths in a single band; rod-shaped paragnaths absent.

Dorsal notopodial ligule not markedly elongate on posterior chaetigers; not markedly broader on posterior chaetigers; markedly reduced on posterior chaetigers, or not markedly reduced on posterior chaetigers. Prechaetal notopodial lobe present, or absent; smaller than dorsal notopodial ligule on anterior chaetigers, usually reduced or absent posteriorly; restricted to a limited number of anterior chaetigers. Notopodial acicular process absent. Dorsal cirrus not sub-terminally attached to dorsal notopodial ligule on posterior chaetigers; not terminally attached to dorsal notopodial ligule on posterior chaetigers; not terminally attached throughout all chaetigers.

Neuropodial prechaetal lobe absent. Neuropodial postchaetal lobe absent. Ventral neuropodial ligule of anterior chaetigers present. Ventral neuropodial ligule of anterior chaetigers approx. as long as acicular neuropodial ligule. Ventral neuropodial ligule on posterior chaetigers present. Ventral neuropodial ligule on posterior chaetigers similar to length of acicular neuropodial ligule, or short, up to half length of acicular neuropodial ligule.

Notoaciculae on chaetigers 1 and 2 absent. Notochaetae: homogomph spinigers present; homogomph falcigers present. Neurochaetae dorsal fascicle: heterogomph spinigers absent; homogomph spinigers present; heterogomph falcigers in anterior chaetigers present, or absent; on posterior chaetigers present; blades serrated; blades with teeth only slightly longer proximally than distally. Neurochaetae ventral fascicle: heterogomph spinigers present, or absent; spinigers in anterior chaetigers with blades evenly serrated throughout; on posterior chaetigers with blades finely serrated proximally; homogomph spinigers absent; heterogomph falcigers present, or absent; anterior chaetigers heterogomph falcigers with long blades present, or absent; anterior chaetigers heterogomph falcigers with extra-long blades present, or absent; anterior chaetigers heterogomph falcigers with short blades absent; posterior chaetigers heterogomph falcigers with long blades present, or absent; posterior chaetigers heterogomph falcigers with extra-long blades present, or absent; posterior chaetigers heterogomph falcigers with short blades present, or absent; heterogomph falcigers blade lacking distinct tendon on terminal tooth.

Anal cirri form cirriform or conical.

#### Remarks.

*Nereis* is the type taxon of the family Nereididae. It is a large assemblage of species, currently with 226 species attributed to the genus, many that probably belong in other genera. There are no revisions of the genus, or parts of it, or any that delineate species into informal subgroups. The description here follows Bakken et al. (2022). Some species are treated as part of revisions of single species or of several similar species (e.g., Salazar-Vallejo et al. 2021), or in treatments of species belonging to the genus in a regional perspective (e.g., Hsueh 2020).

Species of *Nereis* have been found from the littoral zone to abyssal areas, and from a wide range of habitats.

No complete identification guide to species is available but several useful keys of restricted scope have been published: Ramírez-Hernández et al. (2015) has a key to 22 species occurring in the Grand Caribbean, Hsueh (2020) includes a key to 32 species reported from East Asia, and Salazar-Vallejo et al. (2021) has a key to the 11 species previously confused with *N.falsa* de Quatrefages, 1866.

### ﻿ Nicon

Taxon classificationAnimaliaPhyllodocidaNereididae

Kinberg, 1865

C0A85013-959C-5B78-B1A0-AD0552B97E0A

#### Type species.

*Niconpictus* Kinberg, 1865.

#### WoRMS URL.

https://www.marinespecies.org/polychaeta/aphia.php?p=taxdetails&id=173735.

#### Sources.

Pettibone (1971).

#### Diagnosis.

Maxillary ring paragnaths and papillae absent; neuropodial postchaetal lobe present; dorsal notopodial ligule commences chaetiger 3; dorsal notopodial ligule not markedly reduced on posterior chaetigers; oral ring paragnaths absent; notochaetae homogomph falcigers absent (minimal diagnosis; secondary diagnosis not attained).

#### Description.

Palpophore barrel-shaped, approximately equal width from base to palpostyle (not overly large compared with palpostyle). Palpophore surface with a single transverse groove (palpophores well developed). Eyes present, or absent. Tentacular belt greater than length of chaetiger 1.

Jaws with dentate cutting edge.

Maxillary ring of pharynx with papillae and paragnaths absent. Oral ring papillae and paragnaths absent.

Dorsal notopodial ligule present, or absent; not markedly elongate on posterior chaetigers; not markedly broader on posterior chaetigers; not markedly reduced on posterior chaetigers. Prechaetal notopodial lobe present, or absent; smaller than dorsal notopodial ligule on anterior chaetigers, usually reduced or absent posteriorly; restricted to a limited number of anterior chaetigers. Notopodial acicular process absent. Dorsal cirrus not sub-terminally attached to dorsal notopodial ligule on posterior chaetigers; not terminally attached to dorsal notopodial ligule on posterior chaetigers; not terminally attached throughout all chaetigers.

Neuropodial prechaetal lobe absent. Neuropodial postchaetal lobe present; projecting beyond end of the acicular ligule; present throughout all chaetigers, or restricted to anterior chaetigers; digitiform. Ventral neuropodial ligule of anterior chaetigers present. Ventral neuropodial ligule of anterior chaetigers approx. as long as acicular neuropodial ligule. Ventral neuropodial ligule on posterior chaetigers present. Ventral neuropodial ligule on posterior chaetigers similar to length of acicular neuropodial ligule.

Notoaciculae on chaetigers 1 and 2 absent. Notochaetae: homogomph spinigers present. Neurochaetae dorsal fascicle: heterogomph spinigers present, or absent; homogomph spinigers present; heterogomph falcigers in anterior chaetigers present, or absent; on posterior chaetigers present, or absent; blades serrated; simple chaetae (fused falcigers) present, or absent; homogomph falcigers in anterior chaetigers present, or absent. Neurochaetae ventral fascicle: sesquigomph falcigers present, or absent; heterogomph spinigers present, or absent; homogomph spinigers present, or absent; heterogomph falcigers present; anterior chaetigers heterogomph falcigers with long blades absent; anterior chaetigers heterogomph falcigers with extra-long blades present; anterior chaetigers heterogomph falcigers with short blades absent; posterior chaetigers heterogomph falcigers with long blades present, or absent; posterior chaetigers heterogomph falcigers with extra-long blades present, or absent; posterior chaetigers heterogomph falcigers with short blades absent; heterogomph falcigers blade with recurved terminal tooth and distinct tendon, or lacking distinct tendon on terminal tooth; homogomph falcigers in anterior chaetigers present, or absent.

Pygidium bilobate. Anal cirri form cirriform or conical.

#### Remarks.

*Nicon* is not a species-rich genus: of the 700+ species of Nereididae, only ten belong to the genus *Nicon* and those ten species are from diverse habitats and widespread regions (Read and Fauchald 2023). *Nicon* species exhibit more morphologically diversity than seen in many genera of Nereididae and with only a single species included in the most recent study (Wang et al. 2021), both their phylogenetic placement within Nereididae and their monophyly are doubtful.

The two most recent studies describing new *Nicon* species, de León-González and Trovant (2013) and Wang et al. (2021), both also provided keys to the then-known species.

### ﻿ Olganereis

Taxon classificationAnimaliaPhyllodocidaNereididae

Hartmann-Schröder, 1977

E43244D1-78BA-54D0-B351-D007CAE1C1A5

#### Type species.

*Ceratocephalaedmondsi* Hartman, 1954.

#### WoRMS URL.

https://www.marinespecies.org/polychaeta/aphia.php?p=taxdetails&id=324865.

#### Sources.

Hartmann-Schröder (1977).

#### Diagnosis.

Maxillary ring of pharynx with papillae present; dorsal notopodial ligule markedly reduced on posterior chaetigers; ventral neuropodial ligule of anterior chaetigers present (minimal diagnosis). Ventral neuropodial ligule of anterior chaetigers short, up to half length of acicular neuropodial ligule; oral ring papillae present; prostomium anterior margin entire; Area V papillae absent (secondary diagnosis).

#### Description.

Palpophore barrel-shaped, approximately equal width from base to palpostyle (not overly large compared with palpostyle). Tentacular belt greater than length of chaetiger 1.

Jaws with dentate cutting edge.

Maxillary ring of pharynx with papillae present; solitary. Maxillary ring paragnaths absent. Oral ring papillae present. Oral ring papillae arrangement solitary. Area V papillae absent; VI papillae present; VII-VIII papillae present. Oral ring paragnaths absent.

Dorsal notopodial ligule present; commences chaetiger 3; not markedly elongate on posterior chaetigers; not markedly broader on posterior chaetigers; markedly reduced on posterior chaetigers.

Neuropodial prechaetal lobe present. Ventral neuropodial ligule of anterior chaetigers present. Ventral neuropodial ligule of anterior chaetigers short, up to half length of acicular neuropodial ligule. Ventral neuropodial ligule on posterior chaetigers present. Ventral neuropodial ligule on posterior chaetigers similar to length of acicular neuropodial ligule.

Notochaetae: homogomph spinigers present. Neurochaetae dorsal fascicle: heterogomph spinigers absent; homogomph spinigers present; heterogomph falcigers in anterior chaetigers present; on posterior chaetigers present; blades serrated; blades with teeth only slightly longer proximally than distally. Neurochaetae ventral fascicle: heterogomph spinigers present; spinigers in anterior chaetigers with blades evenly serrated throughout; on posterior chaetigers with blades finely serrated proximally; homogomph spinigers absent; heterogomph falcigers present; falcigers blade bowed, with convex margin; heterogomph falcigers blade with recurved terminal tooth and distinct tendon.

Anal cirri form cirriform or conical.

#### Remarks.

*Ceratocephalaedmondsi* Hartman, 1954 (misspelling for *Ceratocephale*) was separated into the new genus *Olganereis* by Hartmann-Schröder (1977) because of the lack of accessory ventral cirri and presence of papillae on both oral and maxillary rings of the pharynx (characters present in *Ceratocephale* species).

*Olganereis* is monotypic and the sole species *O.edmondsi* (Hartman, 1954) occurs in estuaries in southern Australia. The only other Australian nereidid with papillae on both rings of the pharynx is *Dendronereidesheteropoda* Southern, 1921 from tropical estuaries and in which the dorsal notopodial ligule is divided into 'branchial' filaments.

### ﻿ Paraleonnates

Taxon classificationAnimaliaPhyllodocidaNereididae

Khlebovich & Wu, 1962

2AF17D19-1FA0-5B81-BE0E-F9C5A8A432D0

Ganganereis Misra, 1999.
Periserrula Paik, 1977.

#### Type species.

*Paraleonnatesuschakovi* Chlebovitsch & Wu, 1962.

#### WoRMS URL.

https://www.marinespecies.org/polychaeta/aphia.php?p=taxdetails&id=324866.

#### Sources.

Hong et al. (2012).

#### Diagnosis.

Neurochaetae dorsal fascicle homogomph spinigers absent; oral ring papillae present; prostomium anterior margin entire (minimal diagnosis; secondary diagnosis not attained).

#### Description.

Palpophore barrel-shaped, approximately equal width from base to palpostyle (not overly large compared with palpostyle). Palpophore surface with a single transverse groove (palpophores well developed).

Jaws with dentate cutting edge.

Maxillary ring of pharynx with papillae absent. Maxillary ring paragnaths present. Area I conical paragnaths present, or absent; II conical paragnaths present; III conical paragnaths present; III rod-like paragnaths absent; IV paragnaths present; IV conical paragnaths present; IV rod-like paragnaths absent. Oral ring papillae present. Oral ring papillae arrangement solitary. Area V papillae present, or absent; VI papillae present; VII-VIII papillae present. Oral ring paragnaths absent.

Dorsal notopodial ligule not markedly elongate on posterior chaetigers; not markedly broader on posterior chaetigers; not markedly reduced on posterior chaetigers. Prechaetal notopodial lobe present; approximately equal to length of dorsal notopodial ligule at least on anterior chaetigers (thus notopodium of three similar sized ligules/lobes). Dorsal cirrus not sub-terminally attached to dorsal notopodial ligule on posterior chaetigers; not terminally attached to dorsal notopodial ligule on posterior chaetigers; not terminally attached throughout all chaetigers.

Neuropodial postchaetal lobe present; projecting beyond end of the acicular ligule. Ventral neuropodial ligule of anterior chaetigers present. Ventral neuropodial ligule of anterior chaetigers approx. as long as acicular neuropodial ligule. Ventral neuropodial ligule on posterior chaetigers present. Ventral neuropodial ligule on posterior chaetigers similar to length of acicular neuropodial ligule.

Notochaetae: homogomph spinigers present. Neurochaetae dorsal fascicle: heterogomph spinigers present; homogomph spinigers absent. Neurochaetae ventral fascicle: heterogomph spinigers present; homogomph spinigers absent; heterogomph falcigers present, or absent; heterogomph falcigers blade lacking distinct tendon on terminal tooth; homogomph falcigers in anterior chaetigers present, or absent; on posterior chaetigers present, or absent.

Anal cirri form cirriform or conical.

#### Remarks.

The most recent taxonomic treatments of *Paraleonnates* are those of Hong et al. (2012) and Conde-Vela (2019a) which form the basis of the description and diagnosis provided here. *Paraleonnates* is a genus of four species which occur in shallow muddy habitats, typically estuaries and mangroves, in the Indo-Pacific.

Conde-Vela (2019a) provides a key to three species of *Paraleonnates* but omits *Paraleonnatestenuipalpa* (Pflugfelder, 1933) which had been moved to *Paraleonnates* by Glasby et al. (2009); however, Glasby et al. (2009: 15) also note that *P.tenuipalpa* may be a senior synonym of *Paraleonnatesbolus* (Hutchings & Reid, 1991).

### ﻿ Parasetia

Taxon classificationAnimaliaPhyllodocidaNereididae

Villalobos-Guerrero, Conde-Vela & Sato, 2022

3FAA02AD-6AE1-5EC6-8533-F6B145884B80

#### Type species.

*Nereisirritabilis* Webster, 1879.

#### WoRMS URL.

https://www.marinespecies.org/polychaeta/aphia.php?p=taxdetails&id=1600661.

#### Sources.

Villalobos-Guerrero et al. (2022a).

#### Diagnosis.

Neurochaetae dorsal fascicle sesquigomph falcigers present; palpophore massive subconical, flattened (palpostyle is minute by comparison); oral ring papillae absent (minimal diagnosis). Neurochaetae ventral fascicle sesquigomph falcigers present; neuropodial postchaetal lobe not projecting beyond end of the acicular ligule (secondary diagnosis).

#### Description.

Palpophore massive subconical, flattened (palpostyle is minute by comparison). Prostomium longitudinal groove present; anterior region entire, hemispherical, longitudinal groove present; prostomial posterior region as long as anterior region. Tentacular belt greater than length of chaetiger 1.

Oesophageal caeca absent.

Jaws with smooth or slightly crenulate cutting edge.

Everted pharynx a truncate cone, tapering, greatest width at margin of tentacular belt.

Maxillary ring paragnaths present. Area I conical paragnaths absent; II conical paragnaths present; III conical paragnaths present; IV paragnaths present; IV conical paragnaths present. Oral ring paragnaths absent.

Dorsal notopodial ligule not markedly reduced on posterior chaetigers. Prechaetal notopodial lobe present; restricted to a limited number of anterior chaetigers. Notopodial acicular process present, or absent.

Neuropodial prechaetal lobe absent. Neuropodial postchaetal lobe present; not projecting beyond end of the acicular ligule; restricted to anterior chaetigers; digitiform. Ventral neuropodial ligule of anterior chaetigers present. Ventral neuropodial ligule of anterior chaetigers approx. as long as acicular neuropodial ligule. Ventral neuropodial ligule on posterior chaetigers present. Ventral neuropodial ligule on posterior chaetigers similar to length of acicular neuropodial ligule.

Notoaciculae on chaetigers 1 and 2 absent. Notochaetae: homogomph spinigers present. Neurochaetae dorsal fascicle: heterogomph spinigers absent; homogomph spinigers present; sesquigomph falcigers present; heterogomph falcigers in anterior chaetigers absent; on posterior chaetigers absent. Neurochaetae ventral fascicle: sesquigomph falcigers present; heterogomph spinigers present; homogomph spinigers absent; heterogomph falcigers absent; falcigers blade bowed, with convex margin.

Anal cirri form cirriform or conical.

#### Remarks.

*Parasetia* was established by Villalobos-Guerrero et al. (2022a) for *Nereisirritabilis* Webster, 1878, removed from *Composetia* due to absence of oesophageal caeca and other morphological differences from a redefined *Composetia*. *Parasetiairritabilis* (Webster, 1878) occurs on the Atlantic coast of North America down to a depth of ~ 50 m.

### ﻿ Perinereis

Taxon classificationAnimaliaPhyllodocidaNereididae

Kinberg, 1865

085C72C3-6E60-56C1-9E89-8E6F7C7C3512

Arete Kinberg, 1865.
Gnatholycastis Ehlers, 1920.
Lipephile Malmgren, 1867.
Nereis (Lipephile) Malmgren, 1867.
Nereis (Perinereis) auctt.

#### Type species.

*Perinereisnovaehollandiae* Kinberg, 1865.

#### WoRMS URL.

https://www.marinespecies.org/polychaeta/aphia.php?p=taxdetails&id=129380.

#### Sources.

Bakken et al. (2022); de León-González and Goethel (2013).

#### Diagnosis.

Palpophore massive subconical, flattened (palpostyle is minute by comparison); oral ring paragnaths present; Area VI smooth bars present; antennae present (minimal diagnosis; secondary diagnosis not attained).

#### Description.

Palpophore massive subconical, flattened (palpostyle is minute by comparison). Palpophore surface with a single transverse groove (palpophores well developed). Tentacular belt greater than length of chaetiger 1.

Jaws with smooth or slightly crenulate cutting edge or with dentate cutting edge.

Maxillary ring of pharynx with papillae absent. Maxillary ring paragnaths present. Area I conical paragnaths present or absent; II conical paragnaths present or absent; III conical paragnaths present; III rod-like paragnaths absent; IV paragnaths present; IV conical paragnaths present or absent; IV smooth bar-like paragnaths present, or absent; IV rod-like paragnaths absent. Oral ring papillae absent. Oral ring paragnaths present; with Areas V, VI, and VII-VIII discrete; on Areas V and VI form distinct groups. Area V conical paragnaths present, or absent; arranged in a triangle, or in a longitudinal line. Area VI paragnaths present, arranged in lines or arcs; conical paragnaths present, or absent; smooth bars present. Areas VII-VIII paragnaths present, or absent; conical paragnaths present; conical paragnaths arranged in one or more irregular lines forming a continuous band; conical paragnaths similar in size, or irregular mix of large and small paragnaths in a single band; rod-shaped paragnaths absent.

Dorsal notopodial ligule markedly elongate on posterior chaetigers, or not markedly elongate on posterior chaetigers; not markedly broader on posterior chaetigers; not markedly reduced on posterior chaetigers. Prechaetal notopodial lobe present, or absent; smaller than dorsal notopodial ligule on anterior chaetigers, usually reduced or absent posteriorly; restricted to a limited number of anterior chaetigers. Dorsal cirrus sub-terminally attached to dorsal margin of dorsal notopodial ligule on posterior chaetigers, or not sub-terminally attached to dorsal notopodial ligule on posterior chaetigers; not terminally attached to dorsal notopodial ligule on posterior chaetigers; not terminally attached throughout all chaetigers.

Neuropodial prechaetal lobe absent. Neuropodial postchaetal lobe absent, or present; projecting beyond end of the acicular ligule; present throughout all chaetigers, or restricted to anterior chaetigers. Ventral neuropodial ligule of anterior chaetigers present. Ventral neuropodial ligule of anterior chaetigers approx. as long as acicular neuropodial ligule. Ventral neuropodial ligule on posterior chaetigers present. Ventral neuropodial ligule on posterior chaetigers similar to length of acicular neuropodial ligule, or short, up to half length of acicular neuropodial ligule.

Notoaciculae on chaetigers 1 and 2 absent. Notochaetae: homogomph spinigers present. Neurochaetae dorsal fascicle: heterogomph spinigers absent; homogomph spinigers present; heterogomph falcigers in anterior chaetigers present; on posterior chaetigers present; blades serrated; blades with teeth only slightly longer proximally than distally, or much longer proximally than distally. Neurochaetae ventral fascicle: heterogomph spinigers present, or absent; spinigers in anterior chaetigers with blades evenly serrated throughout, or coarsely serrated proximally; on posterior chaetigers with blades finely serrated proximally; homogomph spinigers absent; heterogomph falcigers present; anterior chaetigers heterogomph falcigers with long blades present, or absent; anterior chaetigers heterogomph falcigers with extra-long blades present, or absent; anterior chaetigers heterogomph falcigers with short blades present, or absent; posterior chaetigers heterogomph falcigers with long blades present, or absent; posterior chaetigers heterogomph falcigers with extra-long blades present, or absent; posterior chaetigers heterogomph falcigers with short blades present, or absent; heterogomph falcigers blade lacking distinct tendon on terminal tooth.

Anal cirri form cirriform or conical.

#### Remarks.

The genus *Perinereis* contains 97 species and is thus one of the most species-rich in the family (Elgetany et al. 2022). Species of *Perinereis* are recorded from global locations but are most common in shallow water, particularly where algae occur. There is much morphological diversity within the genus, notably in respect of the form of Area VI paragnaths and of notopodial lobes, so for practical identification purposes informal groupings of species have been proposed (Hutchings et al. 1991). One such grouping, the *Perinereisnuntia* species complex, has been the subject of several recent studies combing morphological and molecular evidence and is probably not monophyletic (Tosuji et al. 2019; Villalobos-Guerrero 2019; Elgetany et al. 2022). The monophyly of the other informal groupings remains untested. Glasby (2015) provides a key to Nereididae from tropical eastern Australia and Villalobos-Guerrero (2019) presents a key to 20 species then known in the *Perinereisnuntia* species complex. Most other keys to species of *Perinereis* are now of limited use since they predate the most recent 20 or so papers which add significantly to knowledge of diversity within the genus.

### ﻿ Platynereis

Taxon classificationAnimaliaPhyllodocidaNereididae

Kinberg, 1865

A780375D-20EC-5099-8D67-DA2BC664E929

Iphinereis Malmgren, 1865.
Pisenoe Kinberg, 1866.
Leontis Malmgren, 1867.
Nectonereis Verrill, 1873.
Uncinereis Chamberlin, 1919.
Nereis (Platynereis) auctt.

#### Type species.

*Platynereismagalhaensis* Kinberg, 1865.

#### WoRMS URL.

https://www.marinespecies.org/polychaeta/aphia.php?p=taxdetails&id=129381.

#### Sources.

Bakken et al. (2022); Read (2007).

#### Diagnosis.

Areas VII-VIII rod-shaped paragnaths present (minimal diagnosis). Notochaetae homogomph falcigers with terminal tendon present (secondary diagnosis).

#### Description.

Palps anteriorly directed, or ventrally directed. Palpophore barrel-shaped, approximately equal width from base to palpostyle (not overly large compared with palpostyle). Palpophore surface with a single transverse groove (palpophores well developed). Tentacular belt greater than length of chaetiger 1.

Jaws with dentate cutting edge.

Maxillary ring of pharynx with papillae absent. Maxillary ring paragnaths present. Area II rod-like paragnaths present; III conical paragnaths absent; III rod-like paragnaths present; IV paragnaths present; IV conical paragnaths absent; IV rod-like paragnaths present. Oral ring papillae present, or absent. Oral ring paragnaths present; on Area V and VI form distinct groups. Area V conical paragnaths absent. Area VI paragnaths present; conical paragnaths absent; rod-shaped paragnaths present. Areas VII-VIII paragnaths present; conical paragnaths absent; conical paragnaths arranged in isolated patches, or in one or more irregular lines forming a continuous band; rod-shaped paragnaths present.

Dorsal notopodial ligule not markedly elongate on posterior chaetigers; not markedly broader on posterior chaetigers; not markedly reduced on posterior chaetigers. Dorsal cirrus sub-terminally attached to dorsal margin of dorsal notopodial ligule on posterior chaetigers; not terminally attached to dorsal notopodial ligule on posterior chaetigers; not terminally attached throughout all chaetigers.

Neuropodial postchaetal lobe absent. Ventral neuropodial ligule of anterior chaetigers present. Ventral neuropodial ligule of anterior chaetigers approx. as long as acicular neuropodial ligule. Ventral neuropodial ligule on posterior chaetigers present. Ventral neuropodial ligule on posterior chaetigers similar to length of acicular neuropodial ligule.

Notoaciculae on chaetigers 1 and 2 absent. Notochaetae: homogomph spinigers present; homogomph falcigers present; with terminal tendon present; articulation fused on some chaetigers (present as a simple chaeta), or with blade free throughout. Neurochaetae dorsal fascicle: heterogomph spinigers absent; homogomph spinigers present; heterogomph falcigers in anterior chaetigers present; on posterior chaetigers present; blades serrated; blades with teeth only slightly longer proximally than distally. Neurochaetae ventral fascicle: heterogomph spinigers present; spinigers in anterior chaetigers with blades evenly serrated throughout; on posterior chaetigers with blades finely serrated proximally; homogomph spinigers absent; heterogomph falcigers present; anterior chaetigers heterogomph falcigers with long blades absent; anterior chaetigers heterogomph falcigers with extra-long blades present; anterior chaetigers heterogomph falcigers with short blades absent; heterogomph falcigers blade lacking distinct tendon on terminal tooth.

Anal cirri form cirriform or conical.

#### Remarks.

Species belonging to *Platynereis* are easily recognised by the small rod-like paragnaths in tight rows on the pharynx; 36 species are recognised. No revisions have been published, but regional studies using molecular data to revise species to resolve the complex taxonomy, and taxonomic history of this genus have been appearing (Wäge et al. 2017; Kara et al. 2020; Teixeira et al. 2022b). Morphological characters from reproductive specimens (epitokes) may be important to distinguish species (Read 2007). Species of *Platynereis* are found in tropical, temperate and sub-Arctic waters, primarily in shallow water among algae.

### ﻿ Potamonereis

Taxon classificationAnimaliaPhyllodocidaNereididae

Villalobos-Guerrero, Conde-Vela & Sato, 2022

2EC55C49-D806-53CF-A576-A0B0617479BE

#### Type species.

*Composetiakumensis* Sato, 2020.

#### WoRMS URL.

https://www.marinespecies.org/polychaeta/aphia.php?p=taxdetails&id=1600677.

#### Sources.

Villalobos-Guerrero et al. (2022a).

#### Diagnosis.

Maxillary ring paragnaths present; neuropodial postchaetal lobe not projecting beyond end of the acicular ligule; notoaciculae on chaetigers 1 and 2 present; prostomium anterior margin entire (minimal diagnosis; secondary diagnosis not attained).

#### Description.

Palpophore barrel-shaped, approximately equal width from base to palpostyle (not overly large compared with palpostyle). Prostomium longitudinal groove present; anterior region entire, hemispherical, longitudinal groove present; prostomial posterior region shorter than anterior region. Tentacular belt greater than length of chaetiger 1.

Oesophageal caeca absent.

Jaws with dentate cutting edge.

Everted pharynx a truncate cone, tapering, greatest width at margin of tentacular belt.

Maxillary ring paragnaths present. Area I conical paragnaths present, or absent; II conical paragnaths present; III conical paragnaths present; III conical paragnaths isolated lateral groups absent; IV paragnaths present; IV conical paragnaths present. Oral ring paragnaths absent.

Prechaetal notopodial lobe absent. Dorsal cirrus not sub-terminally attached to dorsal notopodial ligule on posterior chaetigers.

Neuropodial prechaetal lobe absent. Neuropodial postchaetal lobe present; not projecting beyond end of the acicular ligule; restricted to anterior chaetigers; digitiform. Ventral neuropodial ligule of anterior chaetigers present.

Notoaciculae on chaetigers 1 and 2 present. Notochaetae: homogomph spinigers present. Neurochaetae dorsal fascicle: heterogomph spinigers present, or absent; homogomph spinigers present; heterogomph falcigers in anterior chaetigers present; on posterior chaetigers present. Neurochaetae ventral fascicle: heterogomph spinigers present; homogomph spinigers absent; heterogomph falcigers present; falcigers blade tapering, with straight margin.

Anal cirri form cirriform or conical.

#### Remarks.

*Potamonereis* was established by Villalobos-Guerrero et al. (2022a) for two former species of *Composetia* in which oesophageal caeca are absent and which have a truncate-conical pharynx, and other morphological differences (at the same time those authors redefined *Composetia*). Both species of *Potamonereis* occur in the North-west Pacific in Japanese estuaries.

### ﻿ Pseudonereis

Taxon classificationAnimaliaPhyllodocidaNereididae

Kinberg, 1865

CB56277D-22CA-5D26-8542-5AED85FC6614

Phyllonereis Hansen, 1882.

#### Type species.

*Pseudonereisgallapagensis* Kinberg, 1865.

#### WoRMS URL.

https://www.marinespecies.org/polychaeta/aphia.php?p=taxdetails&id=129382.

#### Sources.

Conde-Vela (2018); Villalobos-Guerrero and Idris (2020).

#### Diagnosis.

Maxillary ring of pharynx with P-bar paragnaths present, usually in regular comb-like rows (minimal diagnosis). Area VI shield-shaped bars present (secondary diagnosis).

#### Description.

Palpophore barrel-shaped, approximately equal width from base to palpostyle (not overly large compared with palpostyle). Palpophore surface with a single transverse groove (palpophores well developed). Tentacular belt greater than length of chaetiger 1.

Jaws with dentate cutting edge.

Maxillary ring of pharynx with papillae absent. Maxillary ring paragnaths present; of pharynx with P-bar paragnaths present, usually in regular comb-like rows. Area I conical paragnaths present; II conical paragnaths present; III conical paragnaths present; III rod-like paragnaths absent; IV paragnaths present; IV conical paragnaths present; IV rod-like paragnaths absent. Oral ring papillae absent. Oral ring paragnaths present; with Areas V, VI and VII-VIII discrete; on Area V and VI form distinct groups. Area V conical paragnaths present, or absent. Area VI paragnaths present; paragnaths arranged in lines or arcs; conical paragnaths present, or absent; smooth bars present, or absent; shield-shaped bars present. Areas VII-VIII paragnaths present; conical paragnaths present; conical paragnaths arranged in one or more irregular lines forming a continuous band; conical paragnaths similar in size, or irregular mix of large and small paragnaths in a single band; P-bar paragnaths absent, or present.

Dorsal notopodial ligule markedly elongate on posterior chaetigers; markedly broader on posterior chaetigers; not markedly reduced on posterior chaetigers. Prechaetal notopodial lobe present, or absent. Notopodial acicular process absent. Dorsal cirrus not sub-terminally attached to dorsal notopodial ligule on posterior chaetigers; terminally attached to dorsal notopodial ligule on posterior chaetigers; not terminally attached throughout all chaetigers.

Neuropodial prechaetal lobe absent. Neuropodial postchaetal lobe absent, or present; projecting beyond end of the acicular ligule; restricted to anterior chaetigers; digitiform, or flattened. Ventral neuropodial ligule of anterior chaetigers present. Ventral neuropodial ligule on posterior chaetigers present. Ventral neuropodial ligule on posterior chaetigers similar to length of acicular neuropodial ligule, or short, up to half length of acicular neuropodial ligule.

Notoaciculae on chaetigers 1 and 2 absent. Notochaetae: homogomph spinigers present; homogomph falcigers present, or absent. Neurochaetae dorsal fascicle: heterogomph spinigers present, or absent; homogomph spinigers present, or absent; heterogomph falcigers in anterior chaetigers present; on posterior chaetigers present; blades serrated; blades with teeth only slightly longer proximally than distally. Neurochaetae ventral fascicle: homogomph spinigers absent; heterogomph falcigers present; heterogomph falcigers blade lacking distinct tendon on terminal tooth.

Anal cirri form cirriform or conical.

#### Remarks.

*Pseudonereis* species are characterised by presence of both P-bars and comb-like rows of paragnaths in Areas II-IV (Villalobos-Guerrero and Idris 2020). The genus was found to be a monophyletic group and could be diagnosed from morphological characters (Bakken and Wilson 2005; Bakken 2007). More species have been described in recent years, and the genus description has been emended (Glasby 2015; Conde-Vela 2018; Villalobos-Guerrero and Idris 2020). The description used here follows Villalobos-Guerrero and Idris (2020). Kara et al. (2018) investigated relationships between several species using molecular data.

Species in this genus are primarily found in tropical and subtropical waters, in shallow depths. Following the last work including revised species, the genus includes 19 species (Villalobos-Guerrero and Idris 2020).

### ﻿ Rullierinereis

Taxon classificationAnimaliaPhyllodocidaNereididae

Pettibone, 1971

F12EB6C0-4AF2-5B97-8987-009087F546CA

Profundilycastis Hartmann-Schröder, 1977.

#### Type species.

*Leptonereiszebra* Rullier, 1963.

#### WoRMS URL.

https://www.marinespecies.org/polychaeta/aphia.php?p=taxdetails&id=129383.

#### Sources.

Pettibone (1971); Tanaka and Sato (2017).

#### Diagnosis.

Notochaetae homogomph falcigers present; maxillary ring paragnaths absent; neurochaetae ventral fascicle heterogomph spinigers absent (minimal diagnosis; secondary diagnosis not attained).

#### Description.

Palpophore barrel-shaped, approximately equal width from base to palpostyle (not overly large compared with palpostyle). Tentacular belt greater than length of chaetiger 1.

Jaws with dentate cutting edge.

Maxillary ring of pharynx with papillae absent. Maxillary ring paragnaths absent. Oral ring papillae present, or absent. Oral ring paragnaths absent.

Dorsal notopodial ligule present; commences chaetiger 3; not markedly elongate on posterior chaetigers (and may be fused with dorsal cirri); not markedly broader on posterior chaetigers; markedly reduced on posterior chaetigers. Dorsal cirrus not sub-terminally attached to dorsal notopodial ligule on posterior chaetigers; not terminally attached to dorsal notopodial ligule on posterior chaetigers; not terminally attached throughout all chaetigers.

Ventral neuropodial ligule of anterior chaetigers present. Ventral neuropodial ligule of anterior chaetigers approx. as long as acicular neuropodial ligule. Ventral neuropodial ligule on posterior chaetigers present. Ventral neuropodial ligule on posterior chaetigers similar to length of acicular neuropodial ligule.

Notochaetae: homogomph spinigers present; homogomph falcigers present (on posterior chaetigers). Neurochaetae dorsal fascicle: heterogomph spinigers absent; homogomph spinigers present; heterogomph falcigers in anterior chaetigers present; on posterior chaetigers present; blades serrated; blades with teeth only slightly longer proximally than distally. Neurochaetae ventral fascicle: heterogomph spinigers absent; homogomph spinigers absent; heterogomph falcigers present; anterior chaetigers heterogomph falcigers with long blades absent; anterior chaetigers heterogomph falcigers with extra-long blades present; anterior chaetigers heterogomph falcigers with short blades absent; posterior chaetigers heterogomph falcigers with long blades present; posterior chaetigers heterogomph falcigers with short blades present.

Anal cirri form cirriform or conical.

#### Remarks.

*Rullierinereis* is a genus with similarities to *Nicon* and *Typhlonereis* but differing in having notopodial homogomph falcigers (Tanaka and Sato 2017). Fifteen species of *Rullierinereis* are recognised and they occur widely around the world from shallow water to abyssal depths (4800 m). Tanaka and Sato (2017) revised the generic description. The only key to species is that of Pettibone (1971), but since that time nine additional species have been described.

### ﻿ Simplisetia

Taxon classificationAnimaliaPhyllodocidaNereididae

Hartmann-Schröder, 1985

71B0A9D9-6CCE-55CB-A33B-8A5C0828603A

Ceratonereis (Simplisetia) Hartmann-Schröder, 1985.

#### Type species.

Nereis (Ceratonereis) aequisetis Augener, 1913.

#### WoRMS URL.

https://www.marinespecies.org/polychaeta/aphia.php?p=taxdetails&id=324869.

#### Sources.

Hartmann-Schröder (1985); Bakken and Wilson (2005).

#### Diagnosis.

Neurochaetae dorsal fascicle simple chaetae (fused falcigers) present; palpophore barrel-shaped, approximately equal width from base to palpostyle (not overly large compared with palpostyle); maxillary ring paragnaths present (minimal diagnosis; secondary diagnosis not attained).

#### Description.

Palpophore barrel-shaped, approximately equal width from base to palpostyle (not overly large compared with palpostyle). Palpophore surface with a single transverse groove (palpophores well developed).

Jaws with dentate cutting edge.

Maxillary ring of pharynx with papillae absent. Maxillary ring paragnaths present. Area I conical paragnaths present, or absent; II conical paragnaths present; III conical paragnaths present; III rod-like paragnaths absent; IV paragnaths present; IV conical paragnaths present; IV rod-like paragnaths absent. Oral ring papillae absent. Oral ring paragnaths absent.

Dorsal notopodial ligule not markedly elongate on posterior chaetigers; not markedly broader on posterior chaetigers (rarely markedly reduced on posterior chaetigers). Prechaetal notopodial lobe present, or absent; smaller than dorsal notopodial ligule on anterior chaetigers, usually reduced or absent posteriorly; restricted to a limited number of anterior chaetigers. Notopodial acicular process present, or absent. Dorsal cirrus not sub-terminally attached to dorsal notopodial ligule on posterior chaetigers; not terminally attached to dorsal notopodial ligule on posterior chaetigers; not terminally attached throughout all chaetigers.

Neuropodial postchaetal lobe absent, or present; projecting beyond end of the acicular ligule; restricted to anterior chaetigers; digitiform. Ventral neuropodial ligule of anterior chaetigers present. Ventral neuropodial ligule of anterior chaetigers approx. as long as acicular neuropodial ligule. Ventral neuropodial ligule on posterior chaetigers present. Ventral neuropodial ligule on posterior chaetigers similar to length of acicular neuropodial ligule, or short, up to half length of acicular neuropodial ligule.

Notoaciculae on chaetigers 1 and 2 absent. Notochaetae: homogomph spinigers present. Neurochaetae dorsal fascicle: heterogomph spinigers present, or absent (only in *S.lizardensis*); homogomph spinigers present; heterogomph falcigers in anterior chaetigers present; on posterior chaetigers present; simple chaetae (fused falcigers) present. Neurochaetae ventral fascicle: heterogomph spinigers present; homogomph spinigers present (only in *Simplisetia* sp. from Phuket), or absent; heterogomph falcigers present; anterior chaetigers heterogomph falcigers with long blades absent; anterior chaetigers heterogomph falcigers with extra-long blades present; anterior chaetigers heterogomph falcigers with short blades absent; posterior chaetigers heterogomph falcigers with long blades present, or absent; posterior chaetigers heterogomph falcigers with extra-long blades present, or absent; posterior chaetigers heterogomph falcigers with short blades present, or absent; heterogomph falcigers blade lacking distinct tendon on terminal tooth.

Anal cirri form cirriform or conical.

#### Remarks.

*Simplisetia* is a genus of estuarine nereidids characterised by the presence of fused neuropodial falcigers in posterior chaetigers and absence of oral ring paragnaths. The fused falcigers are also present in another estuarine genus, *Hediste*, and the two genera may be related (Bakken and Wilson 2005) although they are easily separated by the presence of numerous oral ring paragnaths in *Hediste*.

*Simplisetia* currently includes ten species, seven of which occur in Australian estuaries. Significant differences occur among *Simplisetia* species in the form of the fused falcigers.

The interactive key of Wilson et al. (2003) allowed identification of the Australian species.

### ﻿ Sinonereis

Taxon classificationAnimaliaPhyllodocidaNereididae

Wu & Sun, 1979

560377C8-F34B-57E7-8431-AF6262081185

#### Type species.

*Sinonereisheteropoda* Wu & Sun, 1979.

#### WoRMS URL.

https://www.marinespecies.org/polychaeta/aphia.php?p=taxdetails&id=324879.

#### Sources.

Conde-Vela and Wu (2019).

#### Diagnosis.

Dorsal cirrophores of chaetigers 5–7 of epitokes modified into spherical globular structures (minimal diagnosis). Dorsal notopodial ligule commences chaetiger 4; dorsal notopodial ligule not markedly elongate on posterior chaetigers; notochaetae homogomph falcigers absent (secondary diagnosis).

#### Description.

Palpophore barrel-shaped, approximately equal width from base to palpostyle (not overly large compared with palpostyle).

Jaws with dentate cutting edge.

Maxillary ring of pharynx with papillae absent. Maxillary ring paragnaths absent. Oral ring papillae absent. Oral ring paragnaths absent.

Dorsal notopodial ligule present; commences chaetiger 4; not markedly reduced on posterior chaetigers. Prechaetal notopodial lobe present; present on all chaetigers. Notopodial acicular process absent. Dorsal cirrus not sub-terminally attached to dorsal notopodial ligule on posterior chaetigers.

Neuropodial prechaetal lobe absent. Neuropodial postchaetal lobe present; not projecting beyond end of the acicular ligule; present throughout all chaetigers; digitiform. Ventral neuropodial ligule of anterior chaetigers present. Ventral neuropodial ligule of anterior chaetigers approx. as long as acicular neuropodial ligule. Ventral neuropodial ligule on posterior chaetigers present. Ventral neuropodial ligule on posterior chaetigers similar to length of acicular neuropodial ligule.

Notoaciculae on chaetigers 1 and 2 absent. Notochaetae: homogomph spinigers present. Neurochaetae dorsal fascicle: heterogomph spinigers absent; homogomph spinigers present; heterogomph falcigers in anterior chaetigers present; on posterior chaetigers present. Neurochaetae ventral fascicle: heterogomph spinigers present; homogomph spinigers absent; heterogomph falcigers present; falcigers blade tapering, with straight margin.

Anal cirri form cirriform or conical.

#### Epitokes.

Dorsal cirrophores of chaetigers 5–7 of epitokes modified into spherical globular structures. Natatory epitokal modifications in males commence chaetiger 22. Females without natatory modifications.

#### Remarks.

*Sinonereis* Wu & Sun, 1979 is a monotypic genus originally based on a single epitokous specimen. An emended description including atokous characters was provided by Conde-Vela and Wu (2019).

### ﻿ Solomononereis

Taxon classificationAnimaliaPhyllodocidaNereididae

Gibbs, 1971

B069DACD-C9CC-5050-B536-4D41B71D7E34

#### Type species.

*Solomononereismarauensis* Gibbs, 1971.

#### WoRMS URL.

https://www.marinespecies.org/polychaeta/aphia.php?p=taxdetails&id=324870.

#### Sources.

Nateewathana (1992); Bakken and Wilson (2005).

#### Diagnosis.

Area II rod-like paragnaths present; prostomium anterior margin indented (minimal diagnosis). Notochaetae homogomph falcigers present; dorsal notopodial ligule markedly reduced on posterior chaetigers (secondary diagnosis).

#### Description.

Palpophore barrel-shaped, approximately equal width from base to palpostyle (not overly large compared with palpostyle). Prostomium anterior margin indented. Tentacular belt greater than length of chaetiger 1.

Jaws with dentate cutting edge.

Maxillary ring of pharynx with papillae absent. Maxillary ring paragnaths present. Area I conical paragnaths present; II conical paragnaths absent; II rod-like paragnaths present; III conical paragnaths absent; III rod-like paragnaths present; IV paragnaths present; IV conical paragnaths absent; IV rod-like paragnaths present. Oral ring papillae absent. Oral ring paragnaths absent.

Dorsal notopodial ligule not markedly elongate on posterior chaetigers; not markedly broader on posterior chaetigers; markedly reduced on posterior chaetigers. Prechaetal notopodial lobe absent. Notopodial acicular process absent. Dorsal cirrus not sub-terminally attached to dorsal notopodial ligule on posterior chaetigers; not terminally attached to dorsal notopodial ligule on posterior chaetigers; not terminally attached throughout all chaetigers.

Neuropodial postchaetal lobe absent. Ventral neuropodial ligule of anterior chaetigers present. Ventral neuropodial ligule of anterior chaetigers approx. as long as acicular neuropodial ligule. Ventral neuropodial ligule on posterior chaetigers present. Ventral neuropodial ligule on posterior chaetigers short, up to half length of acicular neuropodial ligule.

Notoaciculae on chaetigers 1 and 2 present. Notochaetae: homogomph spinigers absent; sesquigomph spinigers present; homogomph falcigers present; homogomph falcigers with multidentate blade with 2 or more small lateral teeth, first and subsequent lateral teeth much smaller than terminal tooth present; sesquigomph falcigers present, or absent. Neurochaetae dorsal fascicle: heterogomph spinigers absent; homogomph spinigers absent; sesquigomph spinigers present; heterogomph falcigers in anterior chaetigers absent; on posterior chaetigers present; blades serrated. Neurochaetae ventral fascicle: heterogomph spinigers present; homogomph spinigers absent; heterogomph falcigers absent.

Anal cirri form cirriform or conical.

#### Remarks.

*Solomononereis* is a genus of Nereididae sharing some similarities to members of the larger genus *Ceratonereis*. *Solomononereis*, however, can be distinguished by the presence of rod-like paragnaths on the maxillary ring. *Solomononereis* contains two species, both occurring in the tropical Indo-Pacific to a depth of ~ 30 m. Nateewathana (1992) provides a tabular comparison enabling identification of the species.

### ﻿ Stenoninereis

Taxon classificationAnimaliaPhyllodocidaNereididae

Wesenberg-Lund, 1958

974DE7F1-99C0-5F83-8A37-6CE966A79210

#### Type species.

*Stenoninereismartini* Wesenberg-Lund, 1958.

#### WoRMS URL.

https://www.marinespecies.org/polychaeta/aphia.php?p=taxdetails&id=324872.

#### Sources.

Pettibone (1971); Conde-Vela (2019b).

#### Diagnosis.

Dorsal cirrus arising from basal cirrophore; ventral neuropodial ligule of anterior chaetigers absent; palpophore surface with a single transverse groove (palpophores well developed) (minimal diagnosis). Oral ring papillae absent; maxillary ring paragnaths absent; palpostyles subconical (secondary diagnosis).

#### Description.

Palpophore barrel-shaped, approximately equal width from base to palpostyle (not overly large compared with palpostyle). Prostomium anterior margin indented; longitudinal groove absent. Tentacular belt equal to or less than length of chaetiger 1.

Jaws with dentate cutting edge.

Maxillary ring of pharynx with papillae absent. Maxillary ring paragnaths absent. Oral ring papillae absent. Oral ring paragnaths absent.

Dorsal notopodial ligule present; commences chaetiger 3; markedly reduced on posterior chaetigers. Prechaetal notopodial lobe absent. Notopodial acicular process absent. Dorsal cirrus not sub-terminally attached to dorsal notopodial ligule on posterior chaetigers; arising from basal cirrophore; cirrophore of dorsal cirrus much longer than ventral notopodial ligule (and ciliated); cirrophore of dorsal cirrus cylindrical throughout.

Neuropodial prechaetal lobe absent. Neuropodial postchaetal lobe present; not projecting beyond end of the acicular ligule; present throughout all chaetigers; flattened (rounded). Ventral neuropodial ligule of anterior chaetigers absent. Ventral neuropodial ligule on posterior chaetigers absent.

Notoaciculae on chaetigers 1 and 2 present. Notochaetae: homogomph spinigers absent; sesquigomph spinigers present. Neurochaetae dorsal fascicle: heterogomph spinigers absent; homogomph spinigers absent; sesquigomph spinigers present; heterogomph falcigers in anterior chaetigers absent; on posterior chaetigers absent. Neurochaetae ventral fascicle: heterogomph spinigers present; homogomph spinigers absent; heterogomph falcigers present; falcigers blade tapering, with straight margin; anterior chaetigers heterogomph falcigers with long blades absent; anterior chaetigers heterogomph falcigers with extra-long blades present; anterior chaetigers heterogomph falcigers with short blades absent; posterior chaetigers heterogomph falcigers with long blades absent; posterior chaetigers heterogomph falcigers with extra-long blades present; posterior chaetigers heterogomph falcigers with short blades absent. Neuropodial heterogomph spinigers with blades basally serrate, coarse teeth, larger teeth longer than blade width, 2/3 of the blade edentate and subulate. Neuropodial heterogomph falcigers with very long blades, increasing their length from upper to lower positions in the same fascicle; falcigers with blades smooth.

Anal cirri form cirriform or conical.

#### Remarks.

*Stenoninereis* Wesenberg-Lund, 1958 was described for a single species, *S.martini* Wesenberg-Lund, 1958. The genus now includes four small species, all occurring in sinkholes in the Caribbean-Mexico-central America region. Species of *Stenoninereis* can be identified using the key of Conde-Vela (2019b).

### ﻿ Tambalagamia

Taxon classificationAnimaliaPhyllodocidaNereididae

Pillai, 1961

E4A27572-88EA-56FD-8C0A-F206C7991603

#### Type species.

*Tambalagamiafauveli* Pillai, 1961.

#### WoRMS URL.

https://www.marinespecies.org/polychaeta/aphia.php?p=taxdetails&id=324873.

#### Sources.

Shen and Wu (1993).

#### Diagnosis.

Transverse dorsal lamellae present; neurochaetae dorsal fascicle sesquigomph spinigers absent (minimal diagnosis). Dorsal cirrus arising from basal cirrophore; tentacular belt greater than length of chaetiger 1; notochaetae sesquigomph spinigers absent (secondary diagnosis).

#### Description.

Palpophore barrel-shaped, approximately equal width from base to palpostyle (not overly large compared with palpostyle). Prostomium anterior margin indented. Tentacular belt greater than length of chaetiger 1.

Jaws with smooth or slightly crenulate cutting edge.

Maxillary ring of pharynx with papillae absent. Maxillary ring paragnaths absent. Oral ring papillae present. Oral ring papillae arrangement solitary. Area V papillae present; VII-VIII papillae present. Oral ring paragnaths absent.

Transverse dorsal lamellae present.

Dorsal notopodial ligule present; commences chaetiger 1. Prechaetal notopodial lobe present. Dorsal cirrus arising from basal cirrophore; cirrophore of dorsal cirrus enlarged and vascularised; cirrophore of dorsal cirrus cylindrical throughout.

Ventral neuropodial ligule of anterior chaetigers present. Accessory ventral cirrus present.

Notoaciculae on chaetigers 1 and 2 absent. Notochaetae: homogomph spinigers present. Neurochaetae dorsal fascicle: heterogomph spinigers absent; homogomph spinigers present; heterogomph falcigers in anterior chaetigers absent; on posterior chaetigers absent. Neurochaetae ventral fascicle: heterogomph spinigers absent; homogomph spinigers present; heterogomph falcigers absent.

#### Remarks.

*Tambalagamia* Pillai, 1961 is similar to *Ceratocephale* and, especially to *Gymnonereis*, with double ventral cirri being shared characters. Pettibone (1970) and Hylleberg and Nateewathana (1988) considered *Tambalagamia* to be a junior synonym of *Gymnonereis*. We follow Bakken et al. (2022) and retain *Tambalagamia* as separate pending phylogenetic analysis with better taxon sampling of both genera. *Tambalagamia* currently includes three species which can be identified using the tabular comparison of Shen and Wu (1993).

### ﻿ Tylonereis

Taxon classificationAnimaliaPhyllodocidaNereididae

Fauvel, 1911

D2A469FC-05F5-5D2A-A397-E5D6C969B09D

#### Type species.

*Tylonereisbogoyawlenskyi* Fauvel, 1911.

#### WoRMS URL.

https://www.marinespecies.org/polychaeta/aphia.php?p=taxdetails&id=324874.

#### Sources.

Fauvel (1911).

#### Diagnosis.

Dorsal notopodial ligule markedly broader on posterior chaetigers; prostomium anterior margin indented (minimal diagnosis). Maxillary ring of pharynx with papillae present; dorsal notopodial ligule markedly elongate on posterior chaetigers (secondary diagnosis).

#### Description.

Palpophore barrel-shaped, approximately equal width from base to palpostyle (not overly large compared with palpostyle). Prostomium anterior margin indented.

Jaws with smooth or slightly crenulate cutting edge.

Maxillary ring of pharynx with papillae present; in tufts. Maxillary ring paragnaths absent. Oral ring papillae present. Oral ring papillae arrangement solitary. Area V papillae absent; VI one papillae present; VII-VIII papillae present (in a single row). Oral ring paragnaths absent. Maxillary ring papillae absent on Areas I and II, with double rows on Areas III and IV.

Dorsal notopodial ligule present; commences chaetiger 3; markedly elongate on posterior chaetigers; markedly broader on posterior chaetigers; not markedly reduced on posterior chaetigers. Dorsal cirrus not sub-terminally attached to dorsal notopodial ligule on posterior chaetigers; not terminally attached to dorsal notopodial ligule on posterior chaetigers; not terminally attached throughout all chaetigers.

Ventral neuropodial ligule of anterior chaetigers present. Ventral neuropodial ligule of anterior chaetigers approx. as long as acicular neuropodial ligule. Ventral neuropodial ligule on posterior chaetigers present. Ventral neuropodial ligule on posterior chaetigers similar to length of acicular neuropodial ligule. The neuropodial acicular ligule has three or four distinct lobes; homology of these is unclear, therefore these structures are not scored in this description.

Notoaciculae on chaetigers 1 and 2 absent. Notochaetae: homogomph spinigers present. Neurochaetae dorsal fascicle: heterogomph spinigers absent; homogomph spinigers present; heterogomph falcigers in anterior chaetigers absent; on posterior chaetigers absent. Neurochaetae ventral fascicle: heterogomph spinigers absent; homogomph spinigers present; heterogomph falcigers absent.

#### Remarks.

*Tylonereis* is one of several genera of Nereididae from tropical estuaries which have papillae on both maxillary and oral rings of the pharynx. The genus contains three species, all known from coastal lagoons and lakes of the tropical Indo-Pacific. Tan and Chou (1994) provide a key to species.

### ﻿ Tylorrhynchus

Taxon classificationAnimaliaPhyllodocidaNereididae

Grube, 1866

8F549577-311C-549F-8E5C-EC4B8603F712

#### Type species.

*Nereisheterocheta* Quatrefages, 1866.

#### WoRMS URL.

https://www.marinespecies.org/polychaeta/aphia.php?p=taxdetails&id=324876.

#### Sources.

Pettibone (1971).

#### Diagnosis.

Dorsal cirrus terminally attached throughout, so that dorsal notopodial ligule has appearance of a cirrophore for the dorsal cirrus (minimal diagnosis). Acicular notopodial ligule reduced, much shorter than neuropodial acicular ligule; maxillary ring of pharynx with papillae present (secondary diagnosis).

#### Description.

Palpophore barrel-shaped, approximately equal width from base to palpostyle (not overly large compared with palpostyle). Prostomium anterior margin indented. Tentacular belt greater than length of chaetiger 1.

Jaws with dentate cutting edge.

Maxillary ring of pharynx with papillae present. Maxillary ring paragnaths absent. Oral ring papillae present. Oral ring paragnaths absent.

Dorsal notopodial ligule absent. Prechaetal notopodial lobe absent. Notopodial acicular process absent. Acicular notopodial ligule reduced, much shorter than neuropodial acicular ligule. Dorsal cirrus length ~ 5× acicular notopodial ligule at chaetigers 10–20; not sub-terminally attached to dorsal notopodial ligule on posterior chaetigers; terminally attached throughout, so that dorsal notopodial ligule has appearance of a cirrophore for the dorsal cirrus.

Ventral neuropodial ligule of anterior chaetigers absent. Ventral neuropodial ligule on posterior chaetigers absent. The single notopodial ligule (Pettibone 1971: fig. 25b, c, e) apparently has the notoacicula and therefore cannot be the dorsal notopodial ligule. The homology of the two acicular neuropodial lobes with those of other nereidids is unclear; therefore, these structures are not scored in this description.

Notochaetae: homogomph spinigers absent; sesquigomph spinigers present. Neurochaetae dorsal fascicle: heterogomph spinigers absent; homogomph spinigers absent; sesquigomph spinigers present; heterogomph falcigers in anterior chaetigers present; on posterior chaetigers present; blades serrated. Neurochaetae ventral fascicle: heterogomph spinigers present; spinigers in anterior chaetigers with blades coarsely serrated proximally; on posterior chaetigers with blades coarsely serrated proximally; homogomph spinigers absent; heterogomph falcigers present; anterior chaetigers heterogomph falcigers with long blades absent; anterior chaetigers heterogomph falcigers with extra-long blades present; anterior chaetigers heterogomph falcigers with short blades absent; posterior chaetigers heterogomph falcigers with long blades absent; posterior chaetigers heterogomph falcigers with extra-long blades present; posterior chaetigers heterogomph falcigers with short blades absent.

#### Epitokes.

Epitokes formed by transformation of the anterior part of the body, with the posterior part cast off (Izuka 1903; Pettibone 1971).

#### Remarks.

*Tylorrhynchus* is known from two species, both of which occur in estuarine and fresh waters of the western Pacific Ocean (Japan, China) and the nearby north-east Pacific Ocean (Khlebovich 1996). Additional information on the biology and timing of swarming is provided by Hanafiah et al. (2006). The best taxonomic resources are Izuka (1903) and Pettibone (1971), at which time a single species was recognised. *Tylorrhynchus* is unlike other Nereididae genera in several ways including the absence of a ventral neuropodial ligule.

### ﻿ Typhlonereis

Taxon classificationAnimaliaPhyllodocidaNereididae

Hansen, 1879

58818AE2-C29D-51E2-80C0-75E4DCBD2B5E

#### Type species.

*Typhlonereisgracilis* Hansen, 1879.

#### WoRMS URL.

https://www.marinespecies.org/polychaeta/aphia.php?p=taxdetails&id=324877.

#### Sources.

Bakken (2003).

#### Diagnosis.

Notochaetae of chaetigers 3 and 4 absent (minimal diagnosis). Dorsal notopodial ligule commences chaetiger 5; dorsal notopodial ligule not markedly elongate on posterior chaetigers (secondary diagnosis).

#### Description.

Tentacular belt greater than length of chaetiger 1. Tentacular cirri extend to chaetiger 2 (longest cirrus).

Jaws with dentate cutting edge.

Maxillary ring of pharynx with papillae absent. Maxillary ring paragnaths absent. Oral ring papillae absent. Oral ring paragnaths absent.

Dorsal notopodial ligule present; commences chaetiger 5; not markedly elongate on posterior chaetigers; not markedly broader on posterior chaetigers; markedly reduced on posterior chaetigers. Prechaetal notopodial lobe absent. Notopodial acicular process absent. Dorsal cirrus length ~ 1× acicular notopodial ligule at chaetigers 10–20; sub-terminally attached to dorsal margin of dorsal notopodial ligule on posterior chaetigers; not terminally attached to dorsal notopodial ligule on posterior chaetigers; not terminally attached throughout all chaetigers.

Neuropodial prechaetal lobe absent. Neuropodial postchaetal lobe absent. Ventral neuropodial ligule of anterior chaetigers present. Ventral neuropodial ligule of anterior chaetigers approx. as long as acicular neuropodial ligule. Ventral neuropodial ligule on posterior chaetigers present. Ventral neuropodial ligule on posterior chaetigers similar to length of acicular neuropodial ligule.

Notoaciculae on chaetigers 1 and 2 absent. Notochaetae of chaetigers 3 and 4 absent. Notochaetae: homogomph spinigers present. Neurochaetae dorsal fascicle: heterogomph spinigers absent; homogomph spinigers present; heterogomph falcigers in anterior chaetigers absent; on posterior chaetigers absent. Neurochaetae ventral fascicle: heterogomph spinigers present; homogomph spinigers absent; heterogomph falcigers present; anterior chaetigers heterogomph falcigers with long blades absent; anterior chaetigers heterogomph falcigers with extra-long blades present; anterior chaetigers heterogomph falcigers with short blades absent; posterior chaetigers heterogomph falcigers with long blades absent; posterior chaetigers heterogomph falcigers with extra-long blades present; posterior chaetigers heterogomph falcigers with short blades absent.

#### Remarks.

*Typhlonereis* is known from a single species represented by six specimens from 2222 m in the far North-east Atlantic Ocean (Bakken 2003). The sole species, *Typhlonereisgracilis*, redescribed by Bakken (2003), is similar to *Nicon* and *Rullierinereis* and perhaps will be shown to belong instead in one of those genera.

### ﻿ Unanereis

Taxon classificationAnimaliaPhyllodocidaNereididae

Day, 1962

633E8236-DB9F-5400-872A-6C49C843E94E

#### Type species.

*Unanereismacgregori* Day, 1962.

#### WoRMS URL.

https://www.marinespecies.org/polychaeta/aphia.php?p=taxdetails&id=129384.

#### Sources.

Day (1962).

#### Diagnosis.

Dorsal cirrus terminally attached to dorsal notopodial ligule on posterior chaetigers; dorsal notopodial ligule not markedly broader on posterior chaetigers (minimal diagnosis). Notochaetae sesquigomph falcigers present; prostomium anterior margin entire (secondary diagnosis).

#### Description.

Antennae present (described as having a single antenna but this is here assumed to be a mistake; likely a developmental anomaly or simply missing). Tentacular belt greater than length of chaetiger 1.

Jaws with dentate cutting edge.

Maxillary ring of pharynx with papillae absent. Maxillary ring paragnaths present. Area II conical paragnaths present; III conical paragnaths present; III rod-like paragnaths absent; IV paragnaths present; IV conical paragnaths present; IV rod-like paragnaths absent. Oral ring papillae absent. Oral ring paragnaths absent.

Dorsal notopodial ligule markedly elongate on posterior chaetigers; not markedly broader on posterior chaetigers; not markedly reduced on posterior chaetigers. Dorsal cirrus not sub-terminally attached to dorsal notopodial ligule on posterior chaetigers; terminally attached to dorsal notopodial ligule on posterior chaetigers; not terminally attached throughout all chaetigers.

Neuropodial prechaetal lobe present; extending beyond postchaetal lobe (at least in anterior chaetigers). Neuropodial postchaetal lobe present; projecting beyond end of the acicular ligule; flattened. Ventral neuropodial ligule of anterior chaetigers present. Ventral neuropodial ligule of anterior chaetigers approx. as long as acicular neuropodial ligule. Ventral neuropodial ligule on posterior chaetigers present. Ventral neuropodial ligule on posterior chaetigers short, up to half length of acicular neuropodial ligule.

Notochaetae: homogomph spinigers present; sesquigomph falcigers present; blade distally bifid. Neurochaetae dorsal fascicle: heterogomph spinigers absent; homogomph spinigers present; heterogomph falcigers in anterior chaetigers present; on posterior chaetigers present. Neurochaetae ventral fascicle: heterogomph spinigers present; homogomph spinigers absent; heterogomph falcigers present; heterogomph falcigers blade terminally bifid.

#### Remarks.

*Unanereis* Day, 1962 was described from a single specimen found in the tube of a species of Terebellidae. A second species, *U.zghali* Ben Amor, 1980, from a vertical rocky substrate including dendrophyllid coral *Astroidescalycularis* and algae *Corallinamediterranea* but without an obvious host, has been described from the Mediterranean (Ben Amor 1980). As noted by several studies, the validity of *Unanereis* is doubtful and the genus may represent developmental anomalies in specimens belonging to *Ceratonereis* or *Solomononereis* (Bakken and Wilson 2005; Santos et al. 2005; Bakken et al. 2022).

### ﻿ Websterinereis

Taxon classificationAnimaliaPhyllodocidaNereididae

Pettibone, 1971

306A9DA3-69E0-59E7-B2B1-159E8FFB029B

#### Type species.

*Nereistridentata* Webster, 1879.

#### WoRMS URL.

https://www.marinespecies.org/polychaeta/aphia.php?p=taxdetails&id=129385.

#### Sources.

Pettibone (1971); de León-González and Balart (2016).

#### Diagnosis.

Oral ring papillae present; neurochaetae dorsal fascicle heterogomph falcigers in anterior chaetigers present; maxillary ring paragnaths absent; maxillary ring of pharynx with papillae absent; notochaetae homogomph falcigers absent; oral ring paragnaths absent (minimal diagnosis; secondary diagnosis not attained).

#### Description.

Palpophore barrel-shaped, approximately equal width from base to palpostyle (not overly large compared with palpostyle). Palpophore surface with a single transverse groove (palpophores well developed). Tentacular belt equal to or less than length of chaetiger 1, or greater than length of chaetiger 1.

Jaws with dentate cutting edge.

Maxillary ring of pharynx with papillae absent. Maxillary ring paragnaths absent. Oral ring papillae present. Oral ring papillae arrangement solitary. Area V papillae absent; VI papillae present; VII-VIII papillae present. Oral ring paragnaths absent.

Dorsal notopodial ligule present; commences chaetiger 3; not markedly elongate on posterior chaetigers; not markedly broader on posterior chaetigers; not markedly reduced on posterior chaetigers. Prechaetal notopodial lobe present; smaller than dorsal notopodial ligule on anterior chaetigers, usually reduced or absent posteriorly; restricted to a limited number of anterior chaetigers. Notopodial acicular process absent. Dorsal cirrus not sub-terminally attached to dorsal notopodial ligule on posterior chaetigers; not terminally attached to dorsal notopodial ligule on posterior chaetigers; not terminally attached throughout all chaetigers.

Neuropodial postchaetal lobe present; projecting beyond end of the acicular ligule; present throughout all chaetigers; digitiform. Ventral neuropodial ligule of anterior chaetigers present. Ventral neuropodial ligule of anterior chaetigers approx. as long as acicular neuropodial ligule. Ventral neuropodial ligule on posterior chaetigers present. Ventral neuropodial ligule on posterior chaetigers similar to length of acicular neuropodial ligule.

Notoaciculae on chaetigers 1 and 2 absent. Notochaetae: homogomph spinigers present. Neurochaetae dorsal fascicle: heterogomph spinigers absent; homogomph spinigers present; heterogomph falcigers in anterior chaetigers present; on posterior chaetigers present; blades serrated; blades with teeth only slightly longer proximally than distally. Neurochaetae ventral fascicle: heterogomph spinigers present; homogomph spinigers absent; heterogomph falcigers present; anterior chaetigers heterogomph falcigers with long blades absent; anterior chaetigers heterogomph falcigers with extra-long blades present; anterior chaetigers heterogomph falcigers with short blades absent; posterior chaetigers heterogomph falcigers with long blades present, or absent; posterior chaetigers heterogomph falcigers with extra-long blades absent; posterior chaetigers heterogomph falcigers with short blades present, or absent; heterogomph falcigers blade lacking distinct tendon on terminal tooth.

Anal cirri form cirriform or conical.

#### Remarks.

*Websterinereis* is similar to *Rullierinereis* in having the anterior margin of prostomium entire, the maxillary ring bare and papillae on the oral ring; *Websterinereis* differs from *Rullierinereis* in lacking notopodial homogomph falcigers. There are five species of *Websterinereis* which are known from the Atlantic, Indian, and Pacific Oceans, mostly from shallow water (less than ~ 130 m) except for *W.glauca*, which is recorded to a maximum depth of 3310 m. The genus was revised by de León-González and Balart (2016) who provide a key to species.

### ﻿ Wuinereis

Taxon classificationAnimaliaPhyllodocidaNereididae

Khlebovich, 1996

B6C3393C-54F5-5646-8ECF-8E9D5FEEE89D

#### Type species.

*Leonnatessimplex* Monro, 1939.

#### WoRMS URL.

https://www.marinespecies.org/polychaeta/aphia.php?p=taxdetails&id=1039985.

#### Sources.

Bakken et al. (2022).

#### Diagnosis.

Oral ring papillae present; maxillary ring paragnaths present; ventral neuropodial ligule on posterior chaetigers short, up to half length of acicular neuropodial ligule (minimal diagnosis; secondary diagnosis not attained).

#### Description.

Palpophore barrel-shaped, approximately equal width from base to palpostyle (not overly large compared with palpostyle). Tentacular belt greater than length of chaetiger 1.

Jaws with dentate cutting edge.

Maxillary ring of pharynx with papillae absent. Maxillary ring paragnaths present. Area I conical paragnaths present; II conical paragnaths present; III conical paragnaths present; III conical paragnaths isolated lateral groups absent; IV paragnaths present; IV conical paragnaths present. Oral ring papillae present. Oral ring papillae arrangement solitary. Area V papillae present; VI papillae present; VII-VIII papillae present; VII-VIII papillae arranged in a double row. Oral ring paragnaths absent.

Dorsal notopodial ligule not markedly reduced on posterior chaetigers.

Ventral neuropodial ligule of anterior chaetigers present. Ventral neuropodial ligule of anterior chaetigers approx. as long as acicular neuropodial ligule. Ventral neuropodial ligule on posterior chaetigers present. Ventral neuropodial ligule on posterior chaetigers short, up to half length of acicular neuropodial ligule.

Notoaciculae on chaetigers 1 and 2 absent. Notochaetae: homogomph spinigers present. Neurochaetae dorsal fascicle heterogomph spinigers absent; homogomph spinigers present; heterogomph falcigers in anterior chaetigers present; on posterior chaetigers present. Neurochaetae ventral fascicle: heterogomph spinigers present; homogomph spinigers absent; heterogomph falcigers present; falcigers blade tapering, with straight margin.

#### Remarks.

*Wuinereis* was established by Khlebovich (1996) for a single species formerly placed in *Leonnates*. The two genera (and also *Paraleonnates*) are similar in having only paragnaths on the maxillary ring and only papillae on the oral ring. *Wuinereis* can be separated by chaetal and parapodial characters as per the diagnosis given here. The sole species, *W.simplex* (Monro, 1939) is known only from Aldabra Atoll in the Indian Ocean.

## Supplementary Material

XML Treatment for Alitta

XML Treatment for Australonereis

XML Treatment for Ceratocephale

XML Treatment for Ceratonereis

XML Treatment for Cheilonereis

XML Treatment for Composetia

XML Treatment for Dendronereides

XML Treatment for Dendronereis

XML Treatment for Eunereis

XML Treatment for Gymnonereis

XML Treatment for Hediste

XML Treatment for Imajimainereis

XML Treatment for Kainonereis

XML Treatment for Kinberginereis

XML Treatment for Laeonereis

XML Treatment for Leonnates

XML Treatment for Leptonereis

XML Treatment for Lycastonereis

XML Treatment for Micronereides

XML Treatment for Micronereis

XML Treatment for Namalycastis

XML Treatment for Namanereis

XML Treatment for Neanthes

XML Treatment for Nectoneanthes

XML Treatment for Nereis

XML Treatment for Nicon

XML Treatment for Olganereis

XML Treatment for Paraleonnates

XML Treatment for Parasetia

XML Treatment for Perinereis

XML Treatment for Platynereis

XML Treatment for Potamonereis

XML Treatment for Pseudonereis

XML Treatment for Rullierinereis

XML Treatment for Simplisetia

XML Treatment for Sinonereis

XML Treatment for Solomononereis

XML Treatment for Stenoninereis

XML Treatment for Tambalagamia

XML Treatment for Tylonereis

XML Treatment for Tylorrhynchus

XML Treatment for Typhlonereis

XML Treatment for Unanereis

XML Treatment for Websterinereis

XML Treatment for Wuinereis

## ﻿Additional information

### Author ORCIDs

Robin S. Wilson https://orcid.org/0000-0002-9441-2131

Christopher J. Glasby https://orcid.org/0000-0002-9464-1938

Torkild Bakken https://orcid.org/0000-0002-5188-7305

### Data availability

All of the data that support the findings of this study are available in the main text or Supplementary Information.

## Supplementary materials

Supplementary material 1 Nereididae genera Nexus format Data type nex https://binary.pensoft.net/file/919519This dataset is made available under the Open Database License (http://opendatacommons.org/licenses/odbl/1.0/). The Open Database License (ODbL) is a license agreement intended to allow users to freely share, modify, and use this Dataset while maintaining this same freedom for others, provided that the original source and author(s) are credited.Robin S. Wilson, Christopher J. Glasby, Torkild Bakken

Supplementary material 2 Nereididae species Nexus format Data type nex https://binary.pensoft.net/file/919520This dataset is made available under the Open Database License (http://opendatacommons.org/licenses/odbl/1.0/). The Open Database License (ODbL) is a license agreement intended to allow users to freely share, modify, and use this Dataset while maintaining this same freedom for others, provided that the original source and author(s) are credited.Robin S. Wilson, Christopher J. Glasby, Torkild Bakken
